# Entrepreneurial Regions: Do Macro-Psychological Cultural Characteristics of Regions Help Solve the “Knowledge Paradox” of Economics?

**DOI:** 10.1371/journal.pone.0129332

**Published:** 2015-06-22

**Authors:** Martin Obschonka, Michael Stuetzer, Samuel D. Gosling, Peter J. Rentfrow, Michael E. Lamb, Jeff Potter, David B. Audretsch

**Affiliations:** 1 Department of Psychology, Saarland University, Saarbrücken, Germany; 2 Faculty of Economic Sciences and Media, Ilmenau University of Technology, Ilmenau, Germany; 3 Baden-Württemberg Cooperative State University, Mannheim, Germany; 4 Department of Psychology, University of Texas at Austin, Austin, Texas, United States of America; 5 School of Psychological Sciences, University of Melbourne, Parkville, VIC, Australia; 6 Department of Psychology, University of Cambridge, Cambridge, United Kingdom; 7 Atof Inc., Cambridge, Massachusetts, United States of America; 8 Institute of Developmental Strategies, Indiana University, Bloomington, Indiana, United States of America; University of Amsterdam, NETHERLANDS

## Abstract

In recent years, modern economies have shifted away from being based on physical capital and towards being based on new knowledge (e.g., new ideas and inventions). Consequently, contemporary economic theorizing and key public policies have been based on the assumption that resources for generating knowledge (e.g., education, diversity of industries) are essential for regional economic vitality. However, policy makers and scholars have discovered that, contrary to expectations, the mere presence of, and investments in, new knowledge does not guarantee a high level of regional economic performance (e.g., high entrepreneurship rates). To date, this “knowledge paradox” has resisted resolution. We take an interdisciplinary perspective to offer a new explanation, hypothesizing that “hidden” regional culture differences serve as a crucial factor that is missing from conventional economic analyses and public policy strategies. Focusing on entrepreneurial activity, we hypothesize that the statistical relation between knowledge resources and entrepreneurial vitality (i.e., high entrepreneurship rates) in a region will depend on “hidden” regional differences in entrepreneurial culture. To capture such “hidden” regional differences, we derive measures of entrepreneurship-prone culture from two large personality datasets from the United States (N = 935,858) and Great Britain (N = 417,217). In both countries, the findings were consistent with the knowledge-culture-interaction hypothesis. A series of nine additional robustness checks underscored the robustness of these results. Naturally, these purely correlational findings cannot provide direct evidence for causal processes, but the results nonetheless yield a remarkably consistent and robust picture in the two countries. In doing so, the findings raise the idea of regional culture serving as a new causal candidate, potentially driving the knowledge paradox; such an explanation would be consistent with research on the psychological characteristics of entrepreneurs.

## Introduction

Successful and highly performing local economies generally have one thing in common—strong and robust entrepreneurial activity [1, 2]. There is empirical evidence for a positive correlation between the regional presence of small firms and regional economic development [3, 4]. In particular, in the long run, entrepreneurial activity seems to be related to economic growth (for a review of the literature see [5–7] and for studies applying causal methods see [4, 8]). While scholars debate the different definitions, manifestations, and measurement of entrepreneurship [9–12], leaders in public policy are less nuanced: Entrepreneurship is generally about the startup of new firms and businesses.

Policy makers have “discovered” entrepreneurship—startup activity in the region—as a means for enhancing regional economic development. As Chatterji, Glaeser, and Kerr [13] (p. 1) explain, the success of entrepreneurial clusters in recent decades has challenged the traditional wisdom of smokestack chasing and now many policy makers state that they want their regions to be the next Silicon Valley. This has led to extensive efforts to seed local entrepreneurship, e.g. [14], with today’s politicians routinely announcing the launch of an entrepreneurial cluster in a hot industry. But how can regional entrepreneurship be fostered?

Economists have identified new knowledge (e.g., new ideas and inventions), generated through regional knowledge resources (e.g., human capital and a diverse industry mix), as one key factor contributing to regional variations in entrepreneurial activity [13, 15] because new knowledge creates key opportunities for entrepreneurs to start new firms. However, in what first became known as the “Swedish Paradox,” and subsequently as the “European Paradox”, policy makers and scholars discovered that the mere presence of, and investments in, new knowledge did not guarantee a high level of economic performance [16]. Something was missing.

In this paper we suggest that what has been missing can be found in a very different scholarly literature, psychology. Just as the discipline of economics has traditionally explained economic performance in terms of “hard” factors and inputs, ranging from natural resources, to physical capital, and more recently to knowledge and ideas, the discipline of psychology has focused on “soft” and more “hidden” factors such as culture. Such macro-psychological features of whole regions are receiving growing attention in contemporary psychological science [17–20]. Mapping cultural factors into a spatial context might shed light on the “knowledge paradox” of economics. Regional differences in entrepreneurial culture [1, 21–23] may be a key hidden factor moderating the relationship between knowledge and economic activity; specifically, people in some regions might live in a local culture that predisposes or at least enables them to act upon new knowledge and ideas they have by starting a new business [1]. Thus, in this paper we propose that the degree of entrepreneurship is linked not just to knowledge in the region but rather to the interaction between knowledge and culture. Accordingly, new knowledge will have a greater propensity to generate entrepreneurship in regions with a pronounced entrepreneurial culture where the predominant attitudes and norms reinforce individual’s decisions to act upon entrepreneurial opportunities [24, 25]. Thus, we follow the definition of entrepreneurship put forward by Shane and Venkataraman [10] as the nexus between enterprising individuals and the entrepreneurial opportunity they pursue.

Entrepreneurship research has long considered the potential role of cultural factors. In fact, many economists deem cultural factors a crucial driver of economic performance and development in general [21, 26] and entrepreneurship in particular, e.g., [22, 24, 25, 27–29]. We contribute to this research by employing a personality-based measure of entrepreneurial culture, looking at relatively small spatial units (e.g., cities), and focusing on the interplay between knowledge and culture.

We test our novel knowledge-culture interaction hypothesis in the context of two major economies–the United States (US) and Great Britain (GB). This design allows to test whether the results replicate across two countries and independent datasets. In the following, we first provide the background of our personality-based measure of entrepreneurial culture and then develop our basic hypothesis regarding the interplay between knowledge and an entrepreneurial culture in predicting regional entrepreneurship activity. We then continue by describing the methods, results, and discussion. A series of robustness checks is presented in the S1 Information.

### Quantifying Regional Differences in Entrepreneurial Culture

Culture can be seen as “the collective programming of the mind that distinguishes the members of one group or category of people from another” [30]. In the psychology literature, such collective mental characteristics of populations are assessed in different ways, including via prevalent values [30, 31] or personality characteristics [32, 33]. Prevalent values and personality traits are interacting parts of the local culture as suggested by the Five-Factor Theory of Personality [34] and Rentfrow, Gosling, and Potter’s theory of the emergence, persistence, and expression of geographic variation in personality characteristics [19]. According to these theories and related research, e.g., [35], aggregate-level personality characteristics get expressed via corresponding values and norms in the region. Hofstede and McCrae [35] stress that there is convincing empirical evidence suggesting that “culture-level traits can be legitimately operationalized as the mean of individual trait levels” (p. 79). Following this argument and earlier research on regional personality differences, e.g., [19], we aggregate individual-level personality scores at the regional level to obtain a basic measure of the local entrepreneurial culture. We thus follow the personality-based approach of quantifying regional differences in entrepreneurial culture [24, 28, 29, 36].

In the psychological literature, there are different models of an individual’s personality. Following existing research on individual and regional entrepreneurial personality characteristics [37], we draw from the Five-Factor Theory of Personality (the Big Five traits; [34]), which is the predominant personality model in contemporary psychological science. The Big Five approach is the most cross-culturally validated model of personality [38–41], making it particularly useful for our research comparing entrepreneurship in two separate countries.

Research from the emerging field of the geography of psychology has established that the prevalence of the Big Five traits varies across regions [18]. Moreover, both theorizing on regional personality differences [19] and research investigating the historical roots and trajectories of regional personality features [42] indicate that regional personality differences persist over time. For example, Voigtlaender and Voth show that anti-Semitism in medieval German regions can explain regional patterns of anti-semetic sentiments 500 years later [43]. Becker and Woessmann find that the spread of protestant values after Reformation impact literacy rate of the general populace in 19^th^ century Prussian regions, which in turn predicted economic performance of these regions [44]. Likewise, many regional entrepreneurship scholars argue that a regional entrepreneurial culture persists over time. The litmus test for this argument is the presence of persisting differences in regional entrepreneurship rates that is found in many Western market economies such as the US [45], the UK [46], Germany [47], the Netherlands [48], and Sweden [22]. A recent study by Fritsch and Wyrwich [27], comparing entrepreneurship rates in East Germany from the early 20^th^ century and today’s rates, demonstrates that an entrepreneurial culture can persist several decades and survive even massive social-economic turmoils (i.e., a World War, deep economic recessions, and a long phase of socialism). Most importantly, the correlation between past and contemporary entrepreneurship rates is still significant after controlling for structural economic characteristics that could serve as an alternative explanation for the re-emergence of entrepreneurship in East Germany. This finding suggests that an entrepreneurial culture is deeply rooted in the region [27].

Drawing from earlier research on regional entrepreneurial personality differences [37], we focus on a constellation of the Big Five traits to measure an entrepreneurial personality profile at the individual level and then aggregate it at the regional level. This procedure follows the logic of the person-oriented approach of assessing the individual’s personality [49, 50]. This approach stresses that the configuration of a person’s traits is more than simply the sum of the person’s single traits because the “individual functions as a totality” [50] (p. 463). Seminal theorizing argues that the entrepreneur is best characterized as a specific type, defined by a combination of personality traits [51, 52, 53]. Indeed, there is convincing evidence that an entrepreneurial constellation of the Big Five traits within the person reliably predicts entrepreneurial activity [37]. Such an intraindividual configuration is characterized by high levels of extraversion, conscientiousness, and openness, and lower scores of agreeableness and neuroticism.

It is clear that this personality profile and entrepreneurial behavior are related, but does having a certain personality make entrepreneurial behavior more likely or does the entrepreneurial behavior lead to a change in personality? Research from a number of different traditions clearly points to the causal primacy of personality in this relationship. First, developmental studies have established that the Big Five traits are relatively stable over the life course [54], which is explainable by their strong genetic basis [55]. Consistent with this biological perspective, genetic entrepreneurship studies have identified a link between genes, the Big Five, and entrepreneurial behavior [56]. Thus, the strong genetic base of the Big Five eases endogeneity concerns regarding the region-level relation between the personality profile and economic variables such as entrepreneurial behavior [57]. Second, research has shown that an entrepreneurial constellation of the Big Five traits within the person predicts not only entrepreneurial behavior but also conceptually underlying psychological variables (e.g., entrepreneurial intentions, attitudes, self-efficacy, risk-taking, self-identity, and control beliefs) and economic variables (e.g., entrepreneurial human and social capital) at the individual level (see [37, 58]). Third, evaluation research has shown that those founders scoring low in this entrepreneurial personality profile benefit most from public business advice (e.g., due to a lack of human and social capital, [59]). Fourth, longitudinal research investigating the early formative years of entrepreneurs has found that an entrepreneurial personality profile channels a person’s vocational development towards entrepreneurship in adulthood from early on (e.g., by stimulating early enterprising career interests or early entrepreneurial competencies in adolescence, [60]). This research further shows that the entrepreneurial personality profile, measured as early as in adolescence, is able to prospectively predict entrepreneurial behavior over the subsequent occupational career. Finally, research at the regional level suggests that regions in the US (US states), UK (government regions), and Germany (federal states) scoring higher in the entrepreneurial personality profile also consistently exhibit higher entrepreneurship rates [37]. Specifically, in-depth analyses at the US state-level indicate an interaction between the region’s entrepreneurial personality profile and supportive business conditions (e.g., with regard to venture capital financing, business incubators, labor supply, business costs, infrastructure, and the legal and regulatory environment); those US states exhibiting both a prevalent entrepreneurial culture, indicated by high scores in the entrepreneurial personality profile, and supportive business conditions attain the highest entrepreneurship rates. In summary, the preponderance of evidence suggests that it is personality that drives entrepreneurship, not the other way around.

### The Culture X Knowledge Interaction Hypothesis

Above we referred to Shane and Venkataraman’s view of entrepreneurship as the nexus between opportunities and enterprising individuals. These authors were building on Casson’s [61] definition of opportunities, which views entrepreneurial opportunities as “those situations in which new goods, services, raw materials, and organizing methods can be introduced and sold at greater than their cost of production” [10] (p. 220). Such opportunities often arise from a recombination of existing knowledge pieces to create new knowledge and means-ends relationships. Thus, a region with high knowledge resources might have the potential for more entrepreneurial activity compared to a region with limited knowledge resources [15].

Shane and Venkataraman [10], along with most other scholars of entrepreneurship, make it clear that having knowledge may be necessary for an entrepreneurial opportunity but it is not sufficient to constitute entrepreneurship [9]. Rather, along with the recognition or creation of such an entrepreneurial opportunity, entrepreneurship also requires action or behavior, because any such opportunity must be exploited or actualized in order to be considered *bona fide* entrepreneurship. Thus, we argue that the presence of new knowledge is not a sufficient condition for entrepreneurship. Regions must have a high entrepreneurial culture for people to start businesses based on the available opportunities. This argument builds on Denzau and North’s [62] concept of shared mental models. According to these authors, mental models can be understood as internal representations that are created by one’s cognitive systems. They aid interpretation of the environment and thus have a strong impact on individual decision making. Mental models are not stable. Instead, they are updated by new experiences. Such new experiences can strengthen and confirm initial cognitive representations of the world or they can lead to modifications of the mental model via learning. The shape of the individual mental model is thus shaped by contact with others in one’s environment.

No two individuals are confronted with the same set of experiences in life so different individuals’ mental models will differ. However, Denzau and North [62] argue that cultural heritage “…provides a means of reducing such divergence by encapsulating experience of past generations” [62] (p. 15). This heritage leads to the emergence of so-called shared mental models. These shared mental models are reflected in the values, beliefs, and traits of a community, which are transmitted over generations via socialization at school, by one’s parents, or by contacts with members of the community. These shared mental models will influence the way an individual interprets information, and his or her subsequent decisions [62].

The entrepreneurship culture of a region can be understood as such a shared mental model. Consider the following thought experiment of two regions with the same high endowment of knowledge: Region A has a pronounced entrepreneurship culture. Entrepreneurship is widely approved as a career choice and if a new business fails this is seen as a learning experience, which will increase an entrepreneur’s chances of future success. In region B, entrepreneurship is not viewed as an accepted way to earn a living. In fact, in region B, entrepreneurs might be seen as exploiting labor. Entrepreneurs would be isolated and treated as outsiders of the community. If an entrepreneur fails in region B, he or she might even face discrimination resulting in high financial and psychological costs. For example, failed entrepreneurs in Germany—arguably a country with a low entrepreneurial culture—face difficulties to re-enter entrepreneurship [63] and entrepreneurs in France are penalized at the labor market by receiving lower wages [64]. It is quite evident that in Region B people would think twice before engaging in entrepreneurship, while in Region A people would have less reservations to engage in entrepreneurship.

Summarizing the above, the regional presence of new knowledge seems to be a necessary but not a sufficient condition for entrepreneurship. It still needs entrepreneurial agency in the local population to exploit this knowledge. Hence, it might be the presence of regional entrepreneurship culture that makes people more likely to act entrepreneurially and pursue these knowledge-based opportunities. In regions that have both—high knowledge and high entrepreneurship culture—we should see higher entrepreneurship rates. Accordingly, the main hypothesis of this study holds that there will be a statistical interaction effect between regional knowledge and regional entrepreneurship culture on regional entrepreneurship rates. Specifically, the positive relationship between regional knowledge and regional entrepreneurship rates should be stronger in regions with a high entrepreneurship culture compared to regions with a low entrepreneurship culture.

## Methods

By means of the correlational data we utilize it is possible to determine the degree to which the interaction between knowledge and entrepreneurial culture statistically predicts regional entrepreneurship rates. Do those regions with high entrepreneurship rates indeed show a characteristic pattern of high knowledge *and* entrepreneurial culture levels? Nonetheless, we must stress that these correlational data cannot test for causality. Hence, our analyses should be understood as purely statistical predictions and not as direct evidence for causal effects; of course, the findings may, nonetheless, point to some interesting and potentially new causal candidates (in our case, regional culture).

To examine our interaction hypothesis, we combine and analyze economic and psychological data of regions in the US and GB. In the US, the regional level of analysis is 366 Metropolitan Statistical Areas (MSAs), and in GB it is 375 Local Authority Districts (LADs). In the following, we describe the main variables used in this study. Detailed information on the regional units and the datasets is presented in the Supporting Information Appendix (Section 1 in S1 Information). The SI also contains the descriptive statistics and correlations for all variables.

### Entrepreneurship Rates

Our main dependent variable, *entrepreneurship rate*, is defined by regional start-up rates (number of start-ups / 1,000 employees) in the US and in GB. Regional start-up rates are the most common measure of entrepreneurship at the regional level, e.g., [45, 47, 48], but we should note that it does not capture all entrepreneurship activities covered by Shane and Venkatarman’s [10] definition of entrepreneurship. Our measure of entrepreneurship is based on the *number of start-ups* in a region. Data on US start-ups come from the Statistics of U.S. Businesses (SUSB) provided by the US Census. SUSB covers all US business establishments that have employees [65]. Every business establishment with at least one employee is assigned with a unique identification number and thus can be followed over time. If an establishment hires an employee for the first time, it is assigned with an identification number and counted as a start-up.

In GB we use data from the Inter Departmental Business Register (IDBR). The IDBR is a structured list of UK businesses. IDBR builds on two main data sources—the Value Added Tax (VAT) system from Customs & Excise and Pay As You Earn (PAYE) from Inland Revenue. Supplementary inputs to the IDBR come from official statistics provided by Companies House and ONS business surveys but also from private business information providers. Start-ups are identified by comparing the active business population in two consecutive years—a business not being active in year t-1 but active in year t is regarded as a start-up in year t.

The number of start-ups in a region naturally depends on the region’s population size. We account for this by using entrepreneurship rates, which are computed by the number of start-ups divided by 1,000 employees in the region. Note that in both countries the creation of very small, micro businesses is not captured by the statistical offices. In the US, businesses without employees are not part of the SUSB and thus their creation is not counted as the birth of a new business. In the UK, registration for VAT is only compulsory if a business exceeds certain taxable turnover (in 2012 the threshold was £81,000), a limit smaller firms may not surpass. Thus, our measure of entrepreneurship also includes the creation of larger businesses, which are arguably more impactful in terms of job creation, innovation and economic growth [66]. Note also that such entrepreneurship rates (often called start-up rates) are commonly used to measure entrepreneurship [22, 67, 68] and are often regarded as superior to the rate of self-employment [69].

The choice of year for the entrepreneurship rates is governed by data availability. In the US, the most recent data allow the identification of the 2010 cohort of start-ups at the MSA level. In GB, we use the 2011 start-up data because many independent variables are only available from the Census, which was undertaken in 2011. On average the 2010 entrepreneurship rate in US MSAs is 4.2 start-ups per 1,000 employees with substantial regional variation (min = 2.1, max = 9.2, *SD* = 1.2). In GB, the 2011 entrepreneurship rate is 9.8 start-ups per 1,000 employees (min = 4.7, max = 22.5, *SD* = 2.9).

As a robustness check we considered an alternative measure of entrepreneurship that focuses on high-growth firms. This alternative measure addresses concerns that the start-up rate does not necessarily measure innovative Schumpeterian entrepreneurship [70]. In a recent article, Henrekson and Sanadaji [71] use the presence of Billionaire entrepreneurs from the Forbes *Magazine* as an indicator for Schumpeterian entrepreneurship in a cross-country setting. Unfortunately, this data set is not suitable for within country analyses because the Forbes Magazine lists only the actual residence of the Billionaire entrepreneurs but not the location of the entrepreneurial firms that made them rich. Nevertheless, we follow Henreckson and Sananadji [71] approach of using an unambiguous measure of high-impact entrepreneurship, which in the present paper is based on a list of firms with exceptional growth.

In the US we draw on the 100 fastest growing firms listed by the *Fortune Magazine* in 2014 [72]. To qualify for consideration, firms must meet some criteria regarding revenue ($50 Million) and net income ($10 Million), listing on a US stock exchange and market capitalization ($250 Million), and growth in revenue and earnings per share (larger than 20%) over the last three years. Firms that meet these criteria are then ranked by growth rates for earnings per share, total return, and revenues.

In GB we use a comparable list of 100 fastest growing firms as published by the *Sunday Times* and *Virgin* for 2013 (Fast Track 100) [73]. Qualification criteria for this list comprise annual sales (£250,000) in the base year 2009/2010, annual sales (£5 Million) in 2012/2013, operating profits (£500,000) in 2012/2013, and employment (more than 10) in 2012/2013. The list excludes firms with higher initial sales then £500 Million. Additionally, firms in the technology sector are excluded because the *Sunday Times* ranks the fastest growing firms in the technology sector in a separate list. The qualifying firms are then ranked by sales in growth over a 3-year period.

There are certainly some differences in the listing approaches but firms on both lists definitely fit the criterion of exceptional growth. The lists, thus, make a usable sample of firms with the highest growth across a wide range of industries. For our analysis we locate the headquarters of the firms that allowed us to determine the region (MSAs in the US and LADS in GB). Of the 100 fastest growing firms in the US, 86 were allocatable to an MSA. The remaining 14 firms are either not US based or have their headquarters in a rural area. In contrast to the US, all 100 fastest growing firms in GB were allocatable to an LAD. Both lists with regional allocation are available from the authors on request.

### Entrepreneurial Culture (Entrepreneurial Personality Profile)

In recent years, very big data sets have established the existence of robust regional variation in psychological characteristics; for example, characteristics such as personality and values differ systematically across regions within countries and covary predictably with the economic, social, and institutional parameters of a region [18, 19]. Of particular relevance to the present work, spatial differences in the personality traits that comprise an entrepreneurial personality profile within the single individual have been identified, providing an empirical window onto geographic variation in entrepreneurial culture of regions [37].

These new large psychological datasets now provide the first major opportunity to subject the knowledge-culture interaction hypothesis to an empirical test, by determining the role entrepreneurial culture, and thus the macro-psychological make-up of regions, plays in shaping the impact of knowledge resources on the entrepreneurial activity of regions [18, 21, 22]. We link the region’s entrepreneurial culture, assessed by means of data collected from the broad populace, to the region’s entrepreneurial activity because most entrepreneurs are drawn from local areas and research has demonstrated that the attitudes of the broad populace are the right ones to assess to understand entrepreneurial culture [4].

We used two large independent personality datasets from the US (*N* = 935,858) and GB (*N* = 417,217) that collected individual-level data on the Big Five traits, which is the predominant model of personality in contemporary psychological science [74]. In the US, we used personality data collected within the Gosling-Potter-Internet-Project between 2003 and 2009 [19] (The IRB of the University of Texas at Austin: The study/approval number is: 2004-10-0073. A waiver of informed consent was provided because the study was deemed to minimal risk and no identifying information is collected. Thus it qualifies as an exempt study. The IRB approved this consent procedure #2004-10-0073).

In GB, we used personality data collected between 2009 and 2011 with a large Internet-based survey designed and administered in collaboration with the British Broadcasting Corporation (BBC) (The Psychology Research Ethics Committee of the University of Cambridge approved the research and procedure for obtaining consent in October 2007). Volunteers were told that the survey was designed to assess personality and that by clicking on the link to proceed to the survey they were giving their consent to participate. Informed consent was not requested from the next of kin, caretakers, or guardians on behalf of minors or children because only individuals 18 and older were eligible to participate. Initiating the survey was used as a record of participant consent. This study is part of the BBC LAB UK project (http://www.bbc.co.uk/labuk/), which is a public research project that follows strict ethical standards.

In both countries under study, the Big Five Inventory (BFI; [60]), which consists of 44 short statements designed to assess the prototypical traits defining each of the Five Factor Model dimensions, was used to assess personality. Using a 5-point Likert-type rating scale with endpoints at 1 (*Disagree strongly*) and 5 (*Agree strongly*), respondents indicated the extent to which they agreed with each statement. Analyses of the BFI scales revealed decent internal reliability (*α*s = .82, .76, .79, .79, and .77 in the US and *α*s = .86, .77, .83, .83, and .79 in GB, for Extraversion, Agreeableness, Conscientiousness, Neuroticism, and Openness, respectively).

Following earlier research [37, 75], the entrepreneurial personality profile at the individual level is based on Cronbach and Gleser’s [76] *D* ^2^ approach of quantifying the similarity between two profiles. The individual match between a person's empirical Big Five profile and the fixed reference profile with the extreme scores in each Big Five dimensions, defining the outer limits of the single Big Five traits within an entrepreneurial personality structure (i.e., highest possible value in E, C, O; lowest possible value in A, N). In the first step, each person’s squared differences between the reference values and their personal values on each of the five scales were computed. For instance, if a person scored 3 in neuroticism, the squared difference was 9 (because the reference value was 0). Second, the five squared differences were summed up for each person. Third, the algebraic sign of this sum was reversed (e.g., a value of 20 became -20). The resulting value served as the final variable of the entrepreneurial personality profile, which means that a higher value in this final score signals a stronger entrepreneurial personality structure. These individual scores on the profile were then aggregated to the regional level (average score) to achieve the regional value for the local *entrepreneurial culture*. This index of the entrepreneurial culture of regions had a mean of -20.29 (*SD* = 0.38) across the US regions, and of -20.79 (*SD* = 0.42) across the GB regions. As noted in Obschonka et al. [37], this index offers efficacy in empirical regional studies because it summarizes complex information on the single Big Five traits in one single index, which then is the basis for the aggregated measure of the local entrepreneurial culture. Nevertheless, while this fit measure is rich in information, it is still a relatively broad measure (e.g., it does not consider the individual shape of the empirical Big Five profile of a person but the general deviation from the fixed statistical reference profile). As such, it mirrors the heterogeneity of entrepreneurs and entrepreneurial activities in a given society as well as the broadness of the generally accepted definition of entrepreneurs and entrepreneurial activities in the contemporary entrepreneurship literature.

### Knowledge Resources

The economics literature points to two key features of knowledge resources—human capital and industrial structure. Human capital–how extensively and effectively people invest in their own capacity to generate life-time earnings–represents a population’s potential for productivity based on acquired higher education, job training, or other means of acquiring information. Such factors increase the ability of an individual to think critically, recognize and pursue new opportunities, and to develop new knowledge [77].

We measure *human capital* in the US with the share of the working-age population with a bachelor degree or above, e.g., [2]. Data on qualifications come from 2010 ACS 5yr estimates in the US. In GB, we measure human capital using the share of the working-age population in a region that has a Level-4 qualification or above according to the National Qualifications Framework [78]. A Level-4 qualification is equivalent to a certificate of higher education. The qualification data come from annual population survey in 2011 in GB. Such measures of human capital are widely used in regional economics and are robust predictors for entrepreneurship, e.g., [22, 79]. Beside human capital, seminal theories point to the key role of the regional *industrial structure* for regional economic growth. In particular, theories of agglomeration suggest that the industrial structure affects knowledge creation and innovation, e.g., [80–82]. One approach developed by Jacobs [83] emphasizes the benefits of a diverse industry structure for innovation. New knowledge is often just a recombination of existing knowledge pieces. Industries often rely on different knowledge stocks so a mix of industries in a region offers greater potential for knowledge spillovers and recombination than does a single-industry agglomeration in a region. Put differently, industrial diversity within a region increases the flow of ideas between different industries, fostering the creation of new knowledge through recombination of existing knowledge [3].

In measuring industry diversity, we follow prior research by using the inverse Hirschman-Hefindahl-Index (IHHI)

$$IHHI=\frac{1}{\sum_{i=1}^{N}s_{i}^{2}}$$

where s_i_ is the employment share in the i-th industry sector [84]. In both countries we rely on a broad industry classification scheme (1-digit level, e.g., information and communication industry, construction industry) excluding the agricultural sector and the public administration sector. Data regarding employment in industry sectors come from the 2010 ACS 5yr estimates in the US and the 2011 census in GB. The average IHHI is 7.2 (*SD* = 0.8) in US MSAs and 9.6 (*SD* = 0.7) in GB.

### Control Variables

Beside knowledge and entrepreneurial culture, other regional characteristics can influence entrepreneurial activity. In the regressions, we rely on a standard set of control variables typically used in regional entrepreneurship research.

First, *unemployment* can have positive and negative effects on entrepreneurship. On one hand, unemployment can foster entrepreneurship to the extent that people opt for starting-up a new firm in order to earn a living. Thus, the higher the unemployment is in a region, the more unemployed people might become entrepreneurs. On the other hand, unemployment can be a signal for unfavorable economic conditions, which can dampen incentives for new firm formation [67, 85].

In our regressions, we use the absolute level and the change of the unemployment over time as predictors. This is measured in the US with the mean of the unemployment rate between 2006 and 2010 and the percentage change of the unemployment rate in the same time span. In the US, we use data from 2010 ACS 5yr estimates. Note that in 2006, data on unemployment for one MSA (New Orleans-Metairie-Kenner, LA) were missing. The missing value was replaced by interpolation based on long-run trends in this MSA. In GB we rely on data from the model-based estimates of unemployment provided by ONS between 2007 and 2011. The descriptive statistics reveal that the average unemployment rate between 2006 and 2010 across the US MSAs was 6.7, which increased by 109% (*SD* = 52.2) in this time span. The respective numbers for GB between 2007 and 2011 are 4.9 (*SD* = 1.2) with a comparable upwards trend (average increase of 58%, *SD* = 18.9). The drastic increase in unemployment reflects the impact of the 2008–2009 Great Recession from which regional economies did not recover until 2010/2011.

Second, occupational-choice models argue that people become entrepreneurs if they have higher utility or earnings than in paid employment. Accordingly, high or rising regional purchasing power should matter for entrepreneurship because it signals high or rising demand for products and services. Measures of income or GDP have been shown to be robust predictors for entrepreneurship, e.g., [67, 79, 86].

In our models we use the *absolute level of income* and *change of income over time* as control variables. In the US, we rely on per-capita income data from the US Bureau of Economic Analysis. We compute the mean of the per-capita income between 2006 and 2010 as well as the percentage change of per-capita income during this time. Between 2006 and 2010 US citizens earned on average $35,455 per year in the 366 MSAs (*SD* = $6,538). During that time span, income increased by 7.5% (*SD* = 6.0). In GB, we use the 2007–2011 data from the annual survey of hours and earnings, which reports hourly gross pay and the hours worked per week. Multiplying both variables yields the average weekly earnings. Note that in 6 LADs (Derbyshire Dales, Wealden, Braintree, Daventry, South Northhamptonshire and City of London) income data for some years are not reported. These missing values were interpolated using long run trends in these LADs. Average weekly per capita income in GB LADs was £462 (*SD* = 75) in the years 2007–2011. Within the same time span, income increased by 7% (*SD* = 6.6).

Third, we considered *migration*. Many western societies face increased immigration with substantial economic consequences. Immigrants often face labor market discrimination [87], which can push them into entrepreneurship because they lack alternatives in paid employment. Additionally, a sizeable fraction of immigrants are skilled [88] which can—according to human capital theory—pull people into entrepreneurship. In our analyses we therefore included a control variable representing the regional share of immigrants.

Migration data for the US MSAs come from 2010 ACS 5yr estimates in the US. As a basic indicator for migration we use the percentage of the population who moved to the county from abroad in the year prior to the ACS survey. Using this definition, on average 0.6% of the population of an MSA recently immigrated from abroad (*SD* = 0.5). In GB, comparable questions regarding immigration were available for the Scottish 2011 census but not for the 2011 Census in England and Wales. Thus, we use data from the 2001 Census from which—as in the US—the percentage of immigrants in the year before the census can be computed. We are confident that migration destinations are relatively stable over time because migrants often choose destinations where other migrants already live. Using this data source, 0.5% of the population in the GB LADs recently migrated to the LAD from abroad (SD = 0.4).

Fourth, the *age group 25–44* was considered. Another important influence on regional entrepreneurship is the population’s age structure [89]. It is well known that individual’s tendency to engage in entrepreneurship is age dependent—reaching its peak in the period from the mid 20s to the early 40s [90–92]. People younger than 25 tend to lack skills from work experience and financial resources for entrepreneurship [93] while people older than 44 tend shy away from significant occupational changes [53, 94]. Following this logic we control for the share of the regional population aged 25–44.

Data on the age structure of the regional population stem from 2010 ACS 5yr estimates in the US and the 2011 Census in GB. In the 366 US MSAs the average population share of the 25–44 age group is 26% (*SD* = 2). We observe quite similar numbers in the GB LADs where the population share of the respective age group is 25% (*SD* = 3).

Fifth, we considered *population density and growth*. Population growth is used as a control because a growing population indicates growing demand for products and services, which might drive new entrepreneurial activity, e.g., [79]. Population density is used because it is an excellent control for the general economic prosperity of regions. It is highly correlated with many structural characteristics playing a role in regional entrepreneurship so entrepreneurship scholars regard it a catch-all variable that controls for a range of regional characteristics such as land prices, size of the labor market, and availability of infrastructure, e.g., [67, 16].

Data on the size of the population in regions come from the Census in the US and GB. Population growth is computed as the increase in population between 2000 and 2010 in the US in percent while we use the Census data in 2001 and 2011 in GB.

Finally, we controlled for patterns associated with *larger regions*. We use dummy variables indicating larger regions in both countries. In the US we include dummies for the set of MSAs in the West, Midwest and Northeast. In GB, we include dummies for LADs in England and Wales.

## Results

Here we present the regression results computed separately in the two countries. The dependent variable is the entrepreneurship rate as defined as the number of start-ups per 1,000 employees in a region. All independent variables and control variables (with the exception of the binary regional controls) were z-standardized to avoid multicollinearity. OLS-regression is the standard analytic method in regional entrepreneurship research, e.g., [26, 46–48, 67, 79, 95] so it is the primary method used here. The places (MSAs and LADs) differ in their population sizes resulting in fewer individual observations from less populated regions in the personality datasets. An unwelcome side effect of these size differences is that the average regional scores of the Big Five traits (from which we compute the entrepreneurial-culture indicator) are based on different number of participating individuals (see for details on this Section 1.2). Therefore, all regressions are weighted by the number of respondents per region in the personality data set; this procedure gives greater weight to regions with more observations and thus a more precise measurement of the regional traits [60].

**Fig 1:**
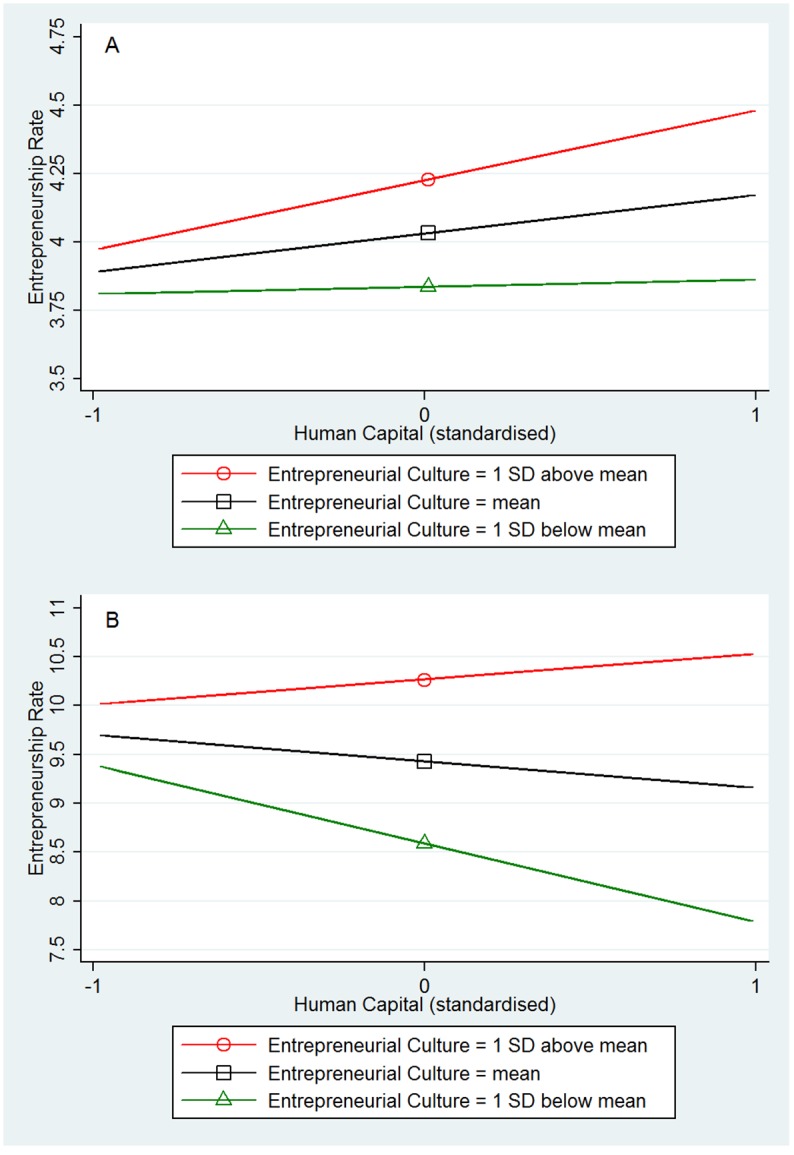
Interaction plots from OLS regression: Human capital X Entrepreneurial culture. (A) Fig 1A (top): US, N = 366. (B) Fig 1B (bottom): GB, N = 375.

**Fig 2:**
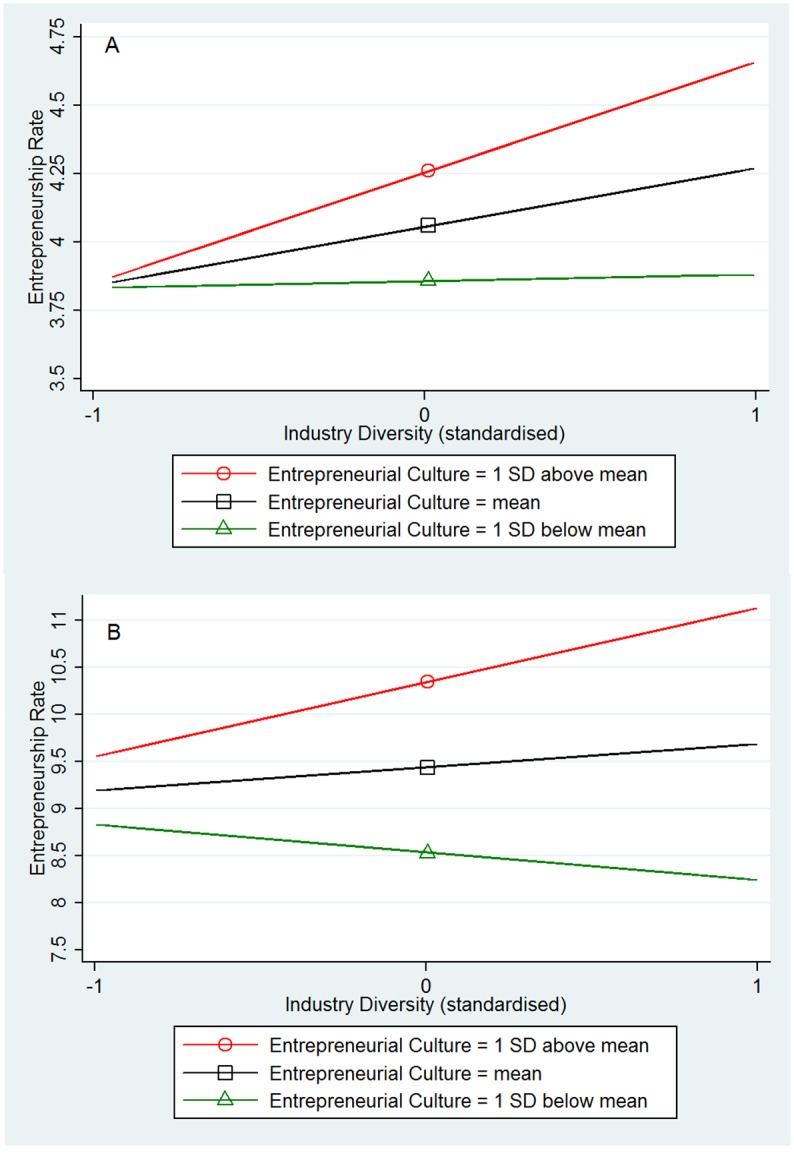
Interaction plots from OLS regression: Industry diversity X Entrepreneurial culture. (A) Fig 2A (top): US, N = 366. (B) Fig 2B (bottom): GB, N = 375.

Table 1 shows the main results from OLS regressions in US regions (Models 1–3) and GB regions (Models 4–6). The models have a high explanatory power by explaining more than 60% of the regions’ variation in entrepreneurship rates. All regressions are weighted by the number of respondents per region in the personality data set, cf., [4]. As shown in Model 1 for the US and Model 4 for GB, human capital had a significant main effect on entrepreneurship rates in the US (B = .19, β = .16, *P* = .016) but not in GB (B = -.24, β = -.06, *P* = .142). Also industry diversity had a positive main effect on entrepreneurship rates in the US (B = .34, β = .32, *P* = .000) but not in GB (B = .23, β = .05, *P* = .104). Entrepreneurial culture had a significant main effect on entrepreneurship rates in both countries (US: B = .22, β = .15, *P* = .000; GB: B = .92, β = .20, *P* = .000). In the next step we test for the hypothesized interaction effects. The interaction between the entrepreneurial culture and the knowledge-creation indexes (human capital: Model 2 for US and Model 5 for GB; industry diversity: Model 3 for US and Model 6 for GB) was significant in both countries (US human capital: B = .11, β = .08, *P* = .038; US industry diversity: B = .19, β = .18, *P* = .000; GB human capital: B = .53, β = .15, *P* = .000; GB industry diversity: B = .54, β = .13, *P* = .000). Consistent with predictions, in both countries the local entrepreneurship rate was highest when high human capital came together with an entrepreneurial culture (Fig 1) and when high industrial diversity came together with an entrepreneurial culture (Fig 2). In fact, the positive effects of human capital and industry diversity are substantially weaker or even vanish in regions where the entrepreneurial culture is weak.

**Table 1:** Start-up rate, human capital, industry diversity, entrepreneurial culture, and interactions.

	Dependent variable: Entrepreneurship rate					
	US			GB		
	Model 1	Model 2	Model 3	Model 4	Model 5	Model 6
Human capital	0.19*	0.14	0.18*	-0.24	-0.27	-0.16
	[0.16]	[0.12]	[0.15]	[-0.06]	[-0.07]	[-0.04]
	(0.08)	(0.08)	(0.08)	(0.17)	(0.16)	(0.16)
Industry diversity	0.34**	0.33**	0.21**	0.23	0.38**	0.25
	[0.32]	[0.32]	[0.20]	[0.05]	[0.08]	[0.05]
	(0.06)	(0.06)	(0.06)	(0.14)	(0.14)	(0.13)
Entrepreneurial culture	0.22**	0.20**	0.20**	0.92**	0.84**	0.90**
	[0.15]	[0.13]	[0.14]	[0.20]	[0.18]	[0.19]
	(0.06)	(0.06)	(0.06)	(0.16)	(0.16)	(0.15)
Interaction: Human capital X		0.11*			0.53**	
Entrepreneurial culture		[0.08]			[0.15]	
		(0.05)			(0.11)	
Interaction: Industry diversity X			0.19**			0.54**
Entrepreneurial culture			[0.18]			[0.13]
			(0.05)			(0.10)
Additional controls	Yes	Yes	Yes	Yes	Yes	Yes
Constant	4.20**	4.18**	4.23**	9.15**	9.06**	8.94**
	(0.07)	(0.07)	(0.07)	(0.37)	(0.36)	(0.36)
Observations	366	366	366	375	375	375
Adjusted R2	0.630	0.634	0.646	0.846	0.855	0.858
F test	45.42**	43.08**	45.49**	159.2**	159.1**	162.6**
AIC	731.5	729	716	1477	1455	1448

The dependent variable is entrepreneurship rate, measured in # start-ups / 1,000 employees in a region. The independent variables are human capital, industry diversity and entrepreneurial culture based on personality data of current residence. Additional control variables include unemployment rate and its change over time, per capita income and its change over time, population density and growth, the share of recently migrated people, the population share of the age group 25–44 and geographic dummy variables (e.g., Region Midwest in the US and Wales in the GB). All variables are z-standardised. Models 1–3 report OLS regressions for the US and Models 4–6 for GB. OLS regressions are weighted by the number of observations per region in the personality data file giving more weight to regions with more observations and a more precise measurement of the entrepreneurial culture variable. Displaying unstandardized coefficients, standardised coefficients in brackets and standard errors in parentheses.

**,* = 1%, 5% significance level.

**Fig 3:**
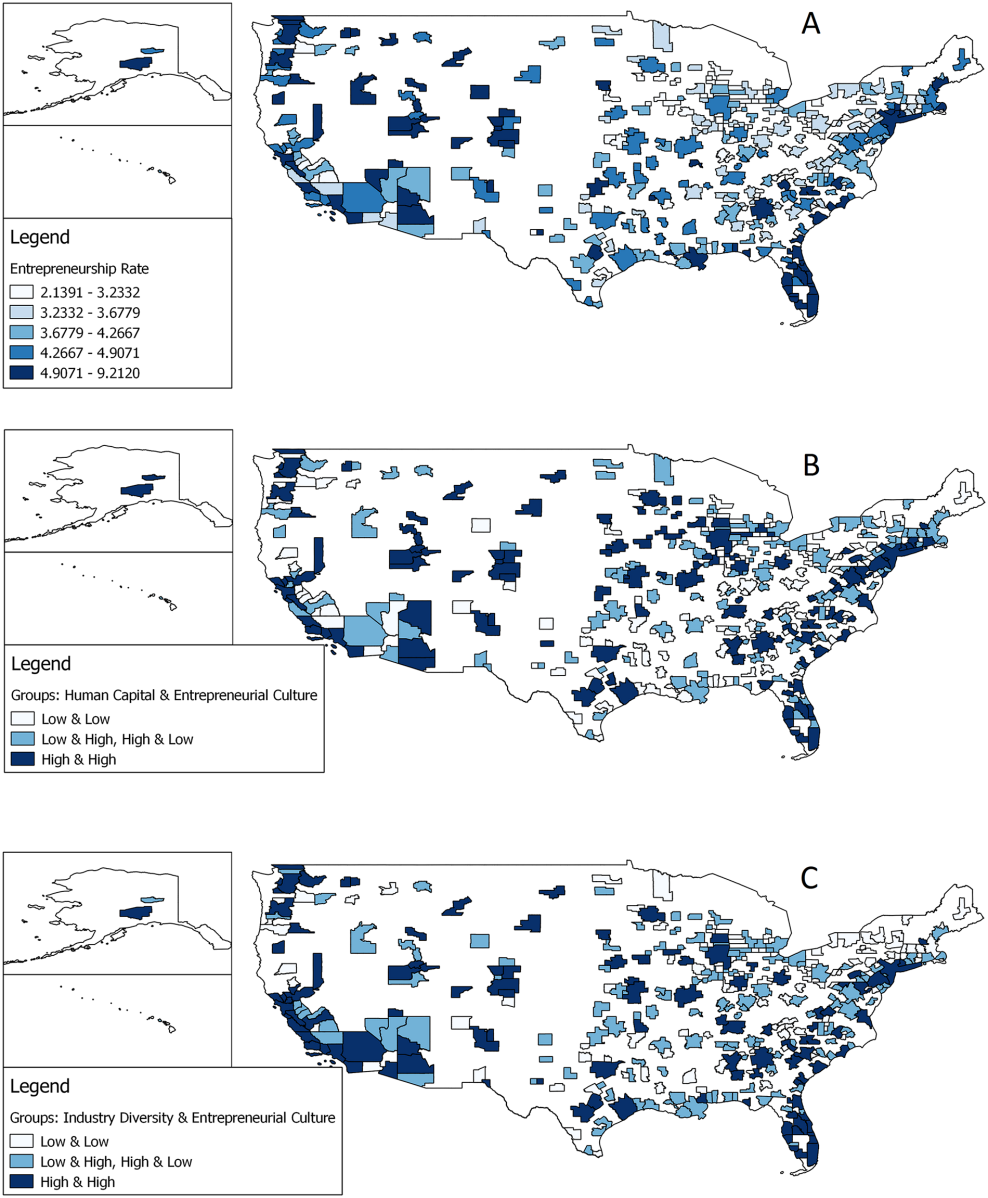
Maps of entrepreneurship rates and the interaction between human capital, industry diversity, and entrepreneurial culture of US regions (N = 366). (A)Fig 3A (top): Entrepreneurship rate in US regions. (B) Fig 3B (middle): Interaction groups between human capital and entrepreneurial culture in US regions. (C) Fig 3C (bottom): Interaction groups between industry diversity and entrepreneurial culture in US regions. Fig 3B should be interpreted as follows: Both variables, human capital and the entrepreneurial culture were splitted at the median. Regions in bright have below median values in human capital and the entrepreneurial culture. Regions in light blue are above median in either human capital or the entrepreneurial culture. Regions in dark blue have above the median values in human capital and entrepreneurial culture. Fig 3C is interpreted in the same way as Fig 3B while interaction groups are created for the variables industry diversity and entrepreneurial culture. The shapefile underlying these maps was kindly provided US Census geography. It contains Ordnance Survey data.

**Fig 4:**
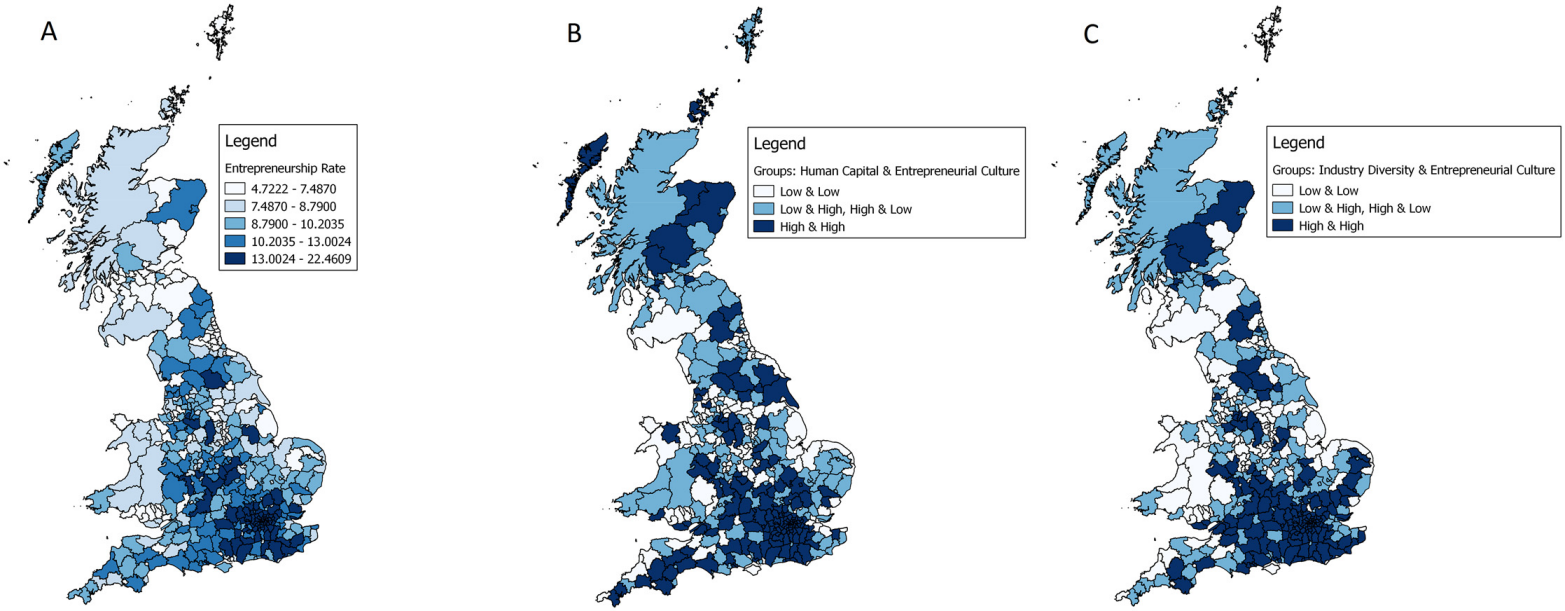
Maps of entrepreneurship rates and the interaction between human capital, industry diversity, and entrepreneurial culture of GB regions (N = 375). (A) Fig 4A (left): Entrepreneurship rate in GB regions. (B) Fig 4B (middle): Interaction groups between human capital and entrepreneurial culture in GB regions. (C) Fig 4C (right): Interaction groups between industry diversity and entrepreneurial culture in GB regions. Fig 4B should be interpreted as follows: Both variables, human capital and the entrepreneurial culture were splitted at the median. Regions in bright have below median values in human capital and the entrepreneurial culture. Regions in light blue are above median in either human capital or the entrepreneurial culture. Regions in dark blue have above the median values in human capital and entrepreneurial culture. Fig 4C is interpreted in the same way as Fig 4B while interaction groups are created for the variables industry diversity and entrepreneurial culture. The shapefile underlying these maps was kindly provided ONS Geography. It contains Ordnance Survey data: Crown copyright and database right 2015.

Fig 3 (US) and 4 (GB) compare maps of the entrepreneurship rates (Figs 3A and 4A), of the interaction between human capital and entrepreneurial culture (Figs 3B and 4B), and of the interaction between industry diversity and entrepreneurial culture (Figs 3C and 4C). In both countries regions with a high/high pattern (high knowledge and high entrepreneurial culture) enjoy comparatively higher entrepreneurship rates. In the US these are regions in Florida, along the Pacific Coast and the Rocky Mountain Regions. The wealthiest region in the US, San Jose–home of Silicon Valley, not only exhibits relatively high entrepreneurship rates but also high levels in both knowledge (human capital, industry diversity) and entrepreneurial culture. In GB the picture is somewhat more nuanced, but London and regions in the South East stand out. In contrast, regions in the US and GB with a combination of low knowledge and a low entrepreneurial culture exhibit low entrepreneurship rates (e.g., many regions in the South and Midwest regions in the US and many regions in Wales, Scotland and the East of England).

Our results show that regions in the US with high levels of knowledge (1 *SD* above mean in human capital and industry diversity) and a high entrepreneurial culture (1 *SD* above mean) have an entrepreneurship rate (startup rate), which is on average 18% higher than in comparable regions with high levels of knowledge but a low entrepreneurial culture (1 *SD* below mean). In GB these differences are even more pronounced. Regions in GB with high levels of knowledge and high entrepreneurial culture have on average a 35% higher entrepreneurship rate compared to regions with high levels of knowledge but low entrepreneurial culture.

We conducted a series of nine robustness checks that consider a) alternative personality-based measures of the local culture, b) alternative spatial levels, c) migration patterns, d) representativeness issues regarding age and gender, and e) alternative explanations of the findings (e.g., whether the region’s employment share in creative occupations confounds our results, [96]). The additional results are presented and discussed in the Supporting Information Appendix (Section 2, Tables A5-A21 in S1 Information). Taken together, these additional tests provide a remarkably consistent picture that supports the statistical validity and robustness of our main findings (i.e., the interaction between knowledge and entrepreneurial culture in the prediction of regional entrepreneurship rates).

Although the start-up rate is a widely used and accepted indicator for general entrepreneurial activity [21, 68]. An alternative measure focuses on high-impact firms [70], which has generally been measured as enterprises with exceptional growth [97]. For example, Henreckson and Sanandaji [71] use the presence of billionaire entrepreneurs in a cross-country analysis of entrepreneurial activity. For our within-country analyses we use a conceptually related measure: the 100 fastest-growing US firms as listed by *Fortune Magazine* [72] and a comparable list of 100 firms with the fastest growth published by the *Sunday Times* and *Virgin* for GB (Fast Track 100). More than 75% of the US-based Fortune 100 firms and 66% of the GB-based Fast Track 100 firms are located in regions with above-median levels for both of the measures of knowledge and entrepreneurial culture. In contrast, less than 5% of the Fortune 100 and less than 15% of the Fast Track 100 firms are from regions with below median levels for both the measures of knowledge and entrepreneurial culture.

At least one Fortune 100 firm was located in around 22% of the regions exhibiting measures of knowledge and culture above the median. By contrast, at least one Fortune 100 firm was located in fewer than 4% of the regions exhibiting measures of knowledge and culture below the median (Section 2, Tables A18 and A19 in S1 Information). Chi2-tests confirm the statistical significance of this difference (χ^2^ = 32.5, *p* < 0.001 for human capital and culture, χ^2^ = 22.0, *p* < 0.001 for industry diversity and culture). A similar pattern was observed for the data from GB (Section 2, Tables A20 and A21 in S1 Information). At least one Fast Track 100 firm was located in around 20% of the regions exhibiting high measures of knowledge and culture. By contrast, this held for fewer than 10% of the regions exhibiting low levels of knowledge and culture. Again this difference is statistically significant (χ^2^ = 7.6, *p* < 0.05 for human capital and culture, χ^2^ = 7.9, *p* < 0.05 for industry diversity and culture). These additional analyses of firms with exceptional growth underscore the statistical robustness of the knowledge-culture interaction effect, even when considering an alternative measure of entrepreneurship.

## Discussion

Our analyses, which were undertaken in two independent samples (in the US and GB), attempted to bridge the two disparate disciplines of economics and psychology by testing the statistical interaction between knowledge and culture in regional entrepreneurship rates. Our correlational data do not permit causal conclusions but they nonetheless revealed a robust and consistent statistical interaction, one which is consistent with theory and research on the psychological characteristics of entrepreneurs. Moreover, the effect survived a wide variety of robustness checks. Hence, even these correlational results can provide candidates to be tested in future research.

Our results have several limitations. First, they are based on correlational data and cannot deliver strictly causal evidence. Second, we investigate only two knowledge resources, human capital and diversity of industries, and future studies could consider other measures of knowledge creation (e.g., investment in R&D, the prevalence of product and service innovations). Third, our study investigates two Western innovation-driven economies. It is unclear whether our results also apply to other economies that are not primarily innovation-driven.

To conclude, future research should continue to integrate “hidden” cultural aspects into economic models of regional performance. We hypothesize that neither knowledge creation nor the local culture alone are responsible for the entrepreneurial vitality, and subsequent economic prosperity, of a region. Rather, the success of a region in the contemporary globalized, innovation-driven economy may depend on the interplay between culture and knowledge creation. If the interaction effect survives causal tests, it would offer a new explanation for the “knowledge paradox” in economics [16]. We further hypothesize that this culture-based perspective implies that a substantial amount of existing new knowledge–the potential for economic prosperity–in regions with lower levels of entrepreneurial culture may remain unexploited. If so, then an entrepreneurial culture might be regarded as a boundary condition for the relationship between knowledge and entrepreneurship.

## Supporting Information

S1 Information Entrepreneurial regions: Do macro-psychological cultural characteristics of regions help solve the “knowledge paradox” of economics? (DOCX)
