# Supplementary material for: Entrepreneurial Regions: Do Macro-Psychological Cultural Characteristics of Regions Help Solve the “Knowledge Paradox” of Economics?
Source: PLoS One. 2015 Jun 22;10(6):e0129332. doi: 10.1371/journal.pone.0129332 (PMC4476658; doi:10.1371/journal.pone.0129332)
Supplement: S1 Information — (DOCX) [file pone.0129332.s001.docx]

**S1 Supporting Information: Entrepreneurial regions: Do macro-psychological cultural characteristics of regions help solve the “knowledge paradox” of economics?**

Martin Obschonka^*^, Michael Stuetzer, Samuel D. Gosling, Peter J. Rentfrow, Michael E. Lamb, Jeff Potter, David B. Audretsch

^*^Corresponding author: Email: martin.obschonka@uni-saarland.de

**Contents**

1. Materials and Methods

1.1Regions

- 1. Psychological Datasets

2. Regression Analyses and Robustness Checks

3. References

1. **Materials and Methods**
   1. **Regions**

For the analyses in the United States (US), Metropolitan Statistical Areas (MSAs) are utilized as the fundamental spatial unit. MSAs are defined by the U.S. Office of Management and Budget as urbanized areas with a population of 50,000 inhabitants or above. Typically an MSA consists of a central county with a high urban population share. Additional adjacent counties are included in the MSAs if they have substantial interaction with the central county measured by commuting flows. Note that using MSAs as a key spatial unit is standard practice in regional entrepreneurship research, e.g., [1,2]. There were 366 metropolitan statistical areas in the US (excluding Puerto Rico) at the end of December 2009.

In Great Britain (GB) there is no officially defined equivalent of MSAs. The closest match to the US definition of MSAs are UK travel-to-work areas, TTWAs [3]. Like in the US, TTWAs are constructed using commuting flows. Unfortunately, the UK National Statistical Office provides only a very limited set of data at the TTWA level. For this reason, we use Local Authority Districts (LADs) as the fundamental spatial unit for the analyses in GB. LADs are administrative units with a substantial degree of local government. LADs are the most fine-grained spatial unit for which entrepreneurship data are reported in GB and have been previously used in entrepreneurship studies, e.g., [4]. Due to missing data for some of our independent variables we excluded the Northern Ireland LADs and the Isle of Scilly from the sample. Additionally, we aggregated all Metropolitan Boroughs (also officially listed as LADs) that form the Greater London region by averaging the values of the variables in the Boroughs . With these adjustments, there were 375 LADs in GB.

It is important to stress these differences in the spatial level between both countries. In the US, we use MSAs which typically consist of several counties with a high degree of integration that together form a “functional region” or city. In contrast, in GB we rely on a set of administrative regions with considerable variation in terms of size and character (urban vs. non-urban). However, in a robustness check (see Section 2 below) we were able bridge the gap in the spatial unit by utilizing a smaller set of 56 English cities for which a best-fit lookup file allocating LADs to TTWAs was available from a UK based research group [5].

- 1. **Psychological datasets**

The measures for the entrepreneurial culture of regions in the US and GB are based on psychological traits of residential populations living in these regions. This section describes the datasets containing the personality trait data and explains the computational procedures for generating the entrepreneurial culture index.

**US**

Personality data on US residents were collected in the Gosling-Potter-Internet-Project [6]. The data were collected via a noncommercial Internet webpage, which can be reached via several channels (e.g., search enginges, unsolicited links on other webpages). US residents can voluntarily participate in this study by completing a questionnaire on socio-demographic variables, personality traits, and state of residence. As an incentive participants received a personality evaluation based on their responses. For this research we utilize data collected between 2003 (the first time respondents were asked to provide their postal code) and 2009.

One of the primary aims of the current investigation was to compute an indicator of an entrepreneurial culture, so we included only those participants who reported living in the US (excluding Puerto Rico), who had completed the questionnaire section on the Big Five traits, which we use to compute this indicator, and who were allocatable to an MSA by ZIP code in the US. This selection criterion resulted in a total sample of 935,858 respondents (63% female). The respondents’ mean age was 26 years (*SD* = 11 years). Regarding race, of those who indicated, 1% were American Indian and Alaska Native, 6% were Asian, 9% were Black, 0.5% Native Hawaiian and other Pacific Islanders, 75% where White and 8% had a different race. 76% of the respondents had a bachelor degree or higher.

Participants reported the ZIP code in which they lived at the time of survey completion. This information was used to determine the county of the respondents. There is no one-to-one link between the ZIP code and administrative regions because ZIP codes are a postal code system and often cross county boundaries. This problem is less severe at the MSA level because roughly 85% of the relevant ZIP codes can be unambigioulsy allocated to either one of the 366 MSAs or to the residual non-urban area. The few ambigious multi-MSA ZIP codes were allocated to the MSA with the higher population. In case of a ZIP code belonging partly to an MSA and non-urban area, the ZIP code was allocated to the respective MSA. The MSA sample sizes ranged between 151 participants from Elmira (NY) and 46,205 participants from New York-Northern New Jersey-Long Island (NY-NJ-PA) (mean = 2,557; median = 1,025).

Then next step consisted of evaluating the representativeness of the Personality sample by comparing the demographic characteristics of the Personality sample with data from the American Community Survey (2010 ACS 5yr estimates) and 2010 Census data at the MSA level. In most cases, we correlated the percentage of respondents in each demographic group from the Personality sample with the percentage of the population from that group within 6each MSA. The correlation between the number of respondents per MSA and the population of the MSA was 0.97. More populated MSAs have more respondents in the Personality data set showing that regions were not systematically over- or underrepresented. The correlation between the share of female respondents and female population share at the MSA level is 0.27. With regard to age, the correlations of the population share in specific age groups at the regional level are 0.12 (under 18 years), 0.72 (18-24 years), 0.34 (25-44 years), 0.43 (45-64 years) and 0.40 (over 65 years). Regarding ethnicity, the correlations for American Indian and Alaska Native, Asian, Black, Native Hawaiian and other Pacific Islanders, and White were 0.93, 0.95, 0.94, 0.97 and 0.91. The correlation between the respondent share with a bachelor degree or higher and the respective population share in the MSA is 0.41. Overall, these results suggest that the Personality sample is fairly representative of the local population regarding ethnicity, education and employment. However, the deviation in some age brackets and the gender imbalance might be a concern. We address this concern in Section 2.1 with a robustness check where we weight the individual respondents in the personality data set – which are used for the computation of the entrepreneurial culture indicator – by age and gender. The results of this robustness check did not differ from our main regressions.

**GB**

Analyses of GB regions were based on data from a large Internet-based survey designed and administered in collaboration with the British Broadcasting Corporation (BBC). Between November 2009 and April 2011, 588,014 individuals completed the “Big Personality Test” which consisted of eight sections covering demographics, education and work, personal relationships, personality and aspirations, health, and childhood experiences. For the present investigation, our analyses focused only on the personality measure.

The survey was advertised and promoted through various BBC websites, radio programs, and television shows. To complete the survey, respondents clicked on a link on the BBC’s *Lab UK* website. Before proceeding, respondents were asked to obtain a BBC ID if they did not already have one. This was used to invite participants to take part in future projects and was also used to prevent individuals from repeat responding (the survey could not be completed more than once with the same ID). After completing the survey, participants received customized feedback about their personalities based on their responses to the survey items.

We included only those participants who reported living in England, Wales, or Scotland, and, replicating the procedure used for US Personality sample, we included only those who had completed the questionnaire section on the Big Five traits and were allocatable to LADs in GB. This selection criterion resulted in a total sample of 417,217 respondents (64% female). The mean age of respondents was 36 years (*SD* = 14 years). Of those who indicated, 4% were Asian, 1% were Black, 2% were of mixed ethnicity, 90% were White and 1% indicated “Other.” In terms of employment and education, 4% of the participants were unemployed and 45% reported completing an undergraduate or postgraduate degree.

Participants reported the postcode and the city or region in which they lived at the time in which they completed the survey. To preserve the anonymity of participants, the BBC made available only the postcode sector (i.e., the complete postcode prefix up to the first letter of the postcode suffix). The postcode information was primarily used to determine the Local Authority District (LAD) in which participants lived. We used the 2008 LAD codes, which included 375 districts across England (aggregating the London Boroughs and excluding The Isles of Scilly), Wales, and Scotland. In cases where no postcode information was available we used data on the city or region in which the participants resided to allocate the respondents to LADs. The LAD sample sizes ranged between 76 participants from Teesdale and 59,733 participants from London (mean = 1,113; median = 777).

To evaluate the representativeness of the samples for the LADs, we compared the demographic characteristics of the LAD samples with 2011 LAD data from the Office of National Statistics and the Scottish Neighbourhood Statistics. The correlation between the number of respondents in a LAD and the population of the LAD is 0.99, indicating that no LAD was over or under-represented in the data. The correlation between the regional share of female participants in the Personality sample with the regional share of the female population is 0.19. Regarding age, the correlations for the age groups are 0.02 (under 18 years), 0.64 (18-24), 0.73 (25-44) 0.74 (45-64) and 0.73 (over 64). With regard to ethnicity, the correlations for Asian, Black, Mixed, and White ethnicities are 0.92, 0.85, 0.74, and .92, respectively. The correlation between unemployment of the Personality respondents and the ONS data is 0.53. For education, the correlation is 0.70. Taken together, these results suggest that the LAD samples are fairly representative of the local population regarding ethnicity, education and employment. As in the US, there are deviation in some age brackets and the gender composition, which might be a concern. We address this concern with a robustness check weighting the individual respondents in the Personality data set by age and gender. As in the US case, the robustness check did not yield different results then the main regressions (see Section 2.1).

- 1. **Economic data**

Detailed information about the sources and measurement procedures for the economic variables are presented in the main paper. Note that we use a comparable set of variables in both countries to achieve comparable results in both countries. Descriptive statistics and correlations for all variables are depicted in Tables A1 (US) and A2 (GB) in S1 Supporting Information.

1. **Regression Analyses and Robustness Checks**

**2.1 Regression analysis**

Table A3 in S1 Supporting Information replicates the results from Table 1 in the Main paper with all control variables displayed. Regarding controls (Model 1 for the US and Model 4 for GB), the change of the unemployment rate is positively correlated with entrepreneurship in the US. This suggests that higher unemployment can push people into entrepreneurship to earn a living. Per capita income is positively related to entrepreneurship rates in GB which can be interpreted as people reacting to signals of rising demand for products and services. Regions with a high share of immigrants have higher entrepreneurship rates in the US which is consistent with both the push and pull explanation why migrants opt for entrepreneurship than paid employment. Contradictory to our expectation, a high population share of the 25-44 age group predicts lower entrepreneurship rates in both the US and GB. Regions with a high population density and population growth enjoy higher entrepreneurship rates in both countries. Regarding our main independent variables, human capital and industry diversity had a positive main effect on entrepreneurship rates in the US but not in GB. Our measure of an entrepreneurial culture is positively related to entrepreneurship rates in the US and GB, suggesting that cultural factors indeed are an important driver of regional entrepreneurship.

In Model 2 (US) and Model 5 (GB) we introduce the interaction term between human capital and entrepreneurial culture into our model. In both countries, the interaction term is positive and significant. The second interaction term between industry diversity and entrepreneurial culture is introduced in Model 3 (US) and Model 6 (GB). Again, we find a positive and significant interaction effect in both countries. As discussed in great detail in the main text, this supports our knowledge-culture interaction hypothesis. Regional entrepreneurship rates are higher when high human capital as well as a high industry diversity come together with a high entrepreneurial culture in the region.

Our regression analysis is weighted by the number of respondents per region in the Personality data set in order to minimize the impact of the small sample sizes in measuring entrepreneurial culture. Table A4 in S1 Supporting Information also presents the results of the unweighted regressions. Of the four interaction terms between knowledge and entrepreneurial culture, three remain positive and statistically significant with the only exception being the interaction between human capital and culture in the US sample.

**2.2 Robustness checks**

We conducted several robustness checks. First, to check whether the results might be affected by the *D^2^* – profile similarity method [7] of assessing the individual entrepreneurial personality profile, we used a simple composite score by adding the mean scores for E, C and O and then subtracting mean scores of A and N for each individual. These individual scores where then again aggregated at the regional level. Re-estimating regressions with this alternative measure of the region’s entrepreneurial culture confirms the majority of the central effects (e.g., the interaction effects). These additional results are depicted in Table A5 in S1 Supporting Information.

Second, following earlier research on personality-based measures of entrepreneurial culture [8], we tested the entrepreneurial personality profile against two "neutral" profiles. In contrast to the entrepreneurial personality profile, these neutral profiles should yield non-meaningful results because there is no underlying theory speaking for an effect of such neutral profiles in our models. The first neutral profile was computed as a neutral *D^2^* profile building on each individual’s *D^2^* deviation from the *middle values of the Big Five Likert scales* (how strong a person's empirical Big Five profile deviates from a fixed reference profile characterized by the simple middle values of the Big Five scales (e.g., a scale between 1 and 5 has a middle value of 3). The results, which are displayed in Table A6 in S1 Supporting Information, show that this neutral profile indeed yields non-meaningful results. The coefficient of the neutral profile is not significant and the coefficients for the interactions between the neutral profile and the knowledge-creation indexes are either not significant or have another than expected direction. We also used a second version of the neutral profile, which utilizes the *mean values* of the empirical distribution of the Big Five traits as fixed reference values. The second neutral profile then represents each individual’s difference from the average *empirical* Big Five scores of the whole sample in each country (and not from the scale middle values like in the other "neutral" profile). These results are displayed in Table A7 in S1 Supporting Information. Again this neutral profile failed to deliver meaningful results. Consistent with prior research [8], these robustness checks testing “neutral” profiles support the validity of the entrepreneurial personality profile as a measure of the local entrepreneurial culture.

Third, to ensure that it is indeed the profile as a whole that is driving the effects, and not the single personality dimensions, we repeated our analyses testing the effects of the single Big Five personality dimensions instead of the profile. The individual traits indeed failed to yield consistent findings (see Table A8 in S1 Supporting Information), which again concurs with prior research [8]. Hence, it is indeed the configural constellation of personality traits that give the entrepreneurial profile its value.

Fourth, the US and GB regressions are based on a somewhat different spatial level. In the US we used MSAs, which typically consist of several counties with a high degree of socio-economic integration that combine to a city. In contrast, in GB we relied on a set of administrative regions that vary considerably in size and character (e.g., urban vs. non-urban). This difference in the spatial level is because no officially defined list of cities exists in GB and thus no economic data are available at such a spatial level. However, in a robustness check we were able to bridge the gap in the spatial level by utilizing a smaller set of 56 English cities for which data could be constructed (more details in Section 1.1). Running regressions on this set of English cities replicated the significant interaction between the knowledge-creation indexes and entrepreneurial culture (see Table A9 in S1 Supporting Information).

Fifth, an alternative explanation for the relevance of a region’s entrepreneurial culture for entrepreneurship rates is that people with an entrepreneurial constellation of traits may move to a region with high start-up rates to take advantage of favorable business conditions to start their business. If one had access to the personality traits of the same respondents before they made any occupational-related migration choices then this selective-migration explanation could be ruled out by showing that the entrepreneurial culture measure still predicts entrepreneurship rates. We lacked longitudinal data on the participants but we did have information on where respondents lived when they were young. We therefore repeated the analyses but this time based on respondents’ youth residence instead of their current residence – the entrepreneurial culture measure of a certain region was assessed by aggregating the individual scores in the entrepreneurial personality profile from those respondents that grew up in this region (irrespectively where they currently live). The results showed again a significant main effect of entrepreneurial culture and significant interaction effects with the knowledge-creation indexes (Table A10 in S1 Supporting Information), which support the initial interpretation of the results.

Sixth, another alternative explanation for the results might be that the interaction between the entrepreneurial culture and knowledge is driven by the share of actual entrepreneurs in the region and not by a prevalent local entrepreneurial culture. In other words, a region could have a high entrepreneurship profile score purely because a lot of entrepreneurs live there and not because the region has an entrepreneurial culture more broadly. However, when we repeated the analyses in GB excluding current entrepreneurs from the sample, the interaction effects were replicated (see Table A11 in S1 Supporting Information; note, occupation information was not available in the US dataset). These analyses support the idea that a region’s entrepreneurial culture is in the “air” rather than bound to the enterprising individuals.

The seventh robustness check extends the idea of the sixth robustness check – capturing the part of entrepreneurial culture that is in the air rather than bound to enterprising individuals – to occupations. Research suggests that personality traits affect occupational decisions (e.g., working in a coal mine vs. being a creative consultant) and that these occupations have specific characteristics such as low capital requirements and low minimum efficient firm sizes that influence individuals decision to be entrepreneurs or work as paid employees in these occupations [9]. To the degree that some occupations share work requirements with entrepreneurship (e.g., taking risks and being creative) the personality profiles of paid employees in certain occupations might be similar to those of entrepreneurs. If additionally these occupations spatially cluster, our observed correlation the culture score and entrepreneurship rates could be driven by the unobserved prevalence of these occupations in the region.

To dispel these concerns, we additionally control for the employment share in creative occupations which should have lower entry barriers for entrepreneurship and similarities in work requirements with entrepreneurship. In the US, the occupation data came from the Bureau of Labor Statistics. We created six different versions of the employment share in creative occupations. The first three are based on the coding of Florida’s [10] for the occupations belonging to the super creative core, the creative professionals and the sum of both henceforth referred to as creative class. The next three are based on an adapted list of occupations of these groups. This adaptation was suggested by McGranahan and Wojan [11] and tries to correct for the fact that some of the original occupations demanded less creativity than those originally thought included in the category. We included these regional employment shares in creative occupations separately into our regression model. To match the time dimensions of many other explanatory variables, we used the average of the 2006-2010 employment shares in the creative occupations. The results are displayed in Table A12 in S1 Supporting Information. Model 1 shows the results of our original regression (Main paper Table 1). Models 2-7 include the different versions of the employment shares in creative occupations. It is evident from these results that the coefficient of the entrepreneurial-culture variable remains significant and does not differ much in size compared to Model 1. This result suggests that the presence of creative occupations (with higher entrepreneurship tendencies) does not drive the relationship between entrepreneurial culture and entrepreneurship rates. Interestingly, the coefficient for the employment shares in creative occupations is negative (and in many cases even significant). The reason for this counterintuitive result is not because there is a negative relationship between creative occupations and entrepreneurial activity. The bivariate correlations of the 6 employment shares in creative occupations with the entrepreneurship rate ranges from +0.02 to +0.32. However there is a very high correlation between the employment shares in creative occupations and our human capital indicator (ranging from +0.51 to +0.73). This is because conceptually and empirically there is a close relationship between human capital and creativity (in the sense described by Florida [10]). Looking back at the examples of creative occupations in the preceding paragraphs reveals that all of them require extensive schooling. The high correlation between creative-class measures and human capital leads to multicollinearity problems in our regression – raising the Variance-inflation factor from 4.5 to above 6. This problem has been also identified by others, e.g., [12] and plagues research in this area.

The multicollinearity problem would be amplified if we additionally included the interaction terms between human capital and the entrepreneurial-personality profile in the regressions. This is because the interaction term is a product of the standardized raw variables and thus contains a lot of characteristics from the original human-capital variable. As a result, coefficient estimates can vary greatly if the regression model is slightly changed. Results of such models should thus be interpreted with great care. Nevertheless we ran these models whose results are displayed in Table A13 in S1 Supporting Information. Model 1 again is a replicate of the Model 1 in Table 1, while Models 2-7 additionally include the employment share in creative class occupations. We find that in most cases the coefficient of the interaction term between human capital and entrepreneurial culture remains positive and is still significant at the 5% or at the 10% level. Note that the interaction between industry diversity and entrepreneurial culture remains completely unchanged when controlling for creative class occupations. Thus we do not report these results here.

The above apparent problems in controlling for occupation-specific characteristics at the regional level can be bypassed in an additional GB analysis. The GB personality data set includes individual level information on personality traits, occupation, and entrepreneurial status (the information on entrepreneurial status is not available in the US personality data set) and thus offers the possibility of conducting such a robustness check at the individual level. Specifically, respondents were asked to select an occupational category (out of 23 possible categories) that best describes their occupation. As expected, there are pronounced differences in the tendency of being an entrepreneur across the different occupations (see Table A14 in S1 Supporting Information for an overview). Furthermore there is a moderate negative correlation (r = -0.26) between the average tendency of being an entrepreneur and the difference between the average entrepreneurial personality profile of the entrepreneur and the average entrepreneurial personality profile of employees across occupations. In other words in occupations with a high entrepreneurial tendency, the personality profile of the self-employed and those in paid employment differ less.

In this robustness check we rely on a subset of the respondents in the personality data set. As we are interested in whether a respondent is an entrepreneur versus a paid employee, we excluded respondents not active at the labour market (e.g., those who were still at school or university, already retired). We additionally excluded a small amount of cases (n=26,295) with missing data on the other key control variables (e.g., ethnicity, gender, education). This results in a sample of 247,232 individuals. Our dependent variable takes the value of 1 if the respondent is self-employed and 0 if the respondent is in paid employment. The key independent variable at the individual level is the entrepreneurial personality profile of the respondent, which is computed as described in the Main paper and in Obschonka et al. [8]. At the individual level, we control for age, gender, income (7 categories), ethnicity (White, Asian, Mixed, Black), and education (dummy variable; 1=respondent has an undergraduate or postgraduate degree, 0=otherwise).

The results of a standard logistic regression are displayed in Table A15 in S1 Supporting Information. Model 1 reports results without the 22 occupational dummy variables. The results suggest that the individual entrepreneurial personality profile is indeed associated with individual entrepreneurship. Those with a higher entrepreneurial personality profile have a higher likelihood to be an entrepreneur. Model 2 includes the 22 dummy variables for the above displayed occupations (the reference category is other occupations). These dummy variables capture all occupation-specific characteristics that can influence individual entrepreneurship and their inclusion sheds light whether the relationship between the individual personality traits weakens once occupation is controlled for. While all coefficients for the occupational dummies are significantly related to the entrepreneurship status, the size of the coefficient of the individual entrepreneurial personality profile drops only slightly. This result suggests that personality matters even after taking occupational characteristics into account.

In the next step we included the regional-level variables, which we use throughout the paper in our regional level regression, in the model. This analysis provides the opportunity to test whether the regional entrepreneurship culture measure is associated with individual entrepreneurship even after taking individual variation in personality into account. Recall that the entrepreneurial-culture measure of a region is simply the average of the individual entrepreneurial-personality profiles of all respondents living in the specific region. Such a test is important for two reasons. Firstly, as we argue in the paper, entrepreneurial culture is fundamentally a collective construct. If our cultural measure explains variation in individual entrepreneurship over and above the individual entrepreneurial personality profile, it strengthens our argumentation of the collective nature of our indicator of entrepreneurial culture. Secondly, entrepreneurship needs human agency. It is individuals and not regions who start businesses. Conducting regressions at the individual level with regional-level predictors considerably weakens the risk of falling prey to the ecological fallacy. Model 3 displays the results after including the regional-level variables. The regional entrepreneurship culture measure is significant. Moreover, the size of the marginal effect of the regional entrepreneurial culture measure is approximately half the size of the individual entrepreneurial-personality profile. Even interpreted conservatively, the results suggest that regional entrepreneurial culture matters and raises the likelihood of every inhabitant of a region to be an entrepreneur.

In Model 4 and 5 we test whether the proposed interaction between knowledge and entrepreneurial culture holds at the individual level. We have argued in the paper that a regional entrepreneurial culture makes individuals in such regions more likely to act upon new business opportunities. This is because individuals are more likely to act entrepreneurially if the regional traits, norms and values are in favor of entrepreneurship. Model 4 includes the interaction term between regional human capital and regional entrepreneurial culture and Model 5 includes the interaction between regional industry diversity and entrepreneurial culture. Although it is tempting to judge the interactions on the size and significance level of the interaction terms (both interaction terms are significant), we are aware that this can be misleading in non-linear models. Unlike standard OLS regressions, the “marginal effect of a change in both interacted variables is not equal to the marginal effect of changing just the interaction term” [13] (p. 154). Furthermore the significance of the interaction term cannot be judged base on the z-statistics. Norton and colleagues developed a more robust method to judge interaction terms. Unfortunately, the respective user programmed Stata “inteff” routine does not produce results in our sample. So we instead relied on the margins procedure in Stata to compute predicted probabilities at different values of regional knowledge and entrepreneurial culture. These predicted probabilities are displayed in Fig. A1 in S1 Supporting Information. Consistent with the hypothesis, the individual likelihood to be an entrepreneur is highest in regions with both high regional knowledge and regional entrepreneurial culture. The relationship between regional knowledge and individual entrepreneurship is much weaker in regions with a low entrepreneurial culture. Individuals living regions with high levels of knowledge and high entrepreneurial culture have on average an 8% higher likelihood of being an entrepreneur compared to regions with high levels of knowledge but low entrepreneurial culture. Additionally, at high levels of regional human capital and regional industry diversity the 95% confidence intervals of the predicted probabilities of being an entrepreneur do not overlap when comparing regions with high and low entrepreneurial culture (summarized in Table A16 in S1 Supporting Information). This result suggests that the interactions are significant. Note that this result is controlled for the individual human capital of a person as well as the individual entrepreneurial personality profile.

One drawback of the models reported above is the use of a standard logistic regression for the analysis. Our data have a nested structure – individuals living in certain regions. Multi-level designs are better equipped to handle this embeddedness [14]. Following others we therefore applied a random-effect logistic regression model [15-17] and allowed the intercept to vary across regions. The results of the repeated test of the interaction hypothesis with multi-level methods are displayed in Models 6 and 7 (Table A15 in S1 Supporting Information). Again, we computed predicted probabilities at different values of regional knowledge and entrepreneurial culture. The plot of these predicted probabilities does not differ from the plots in Fig. S1 in S1 Supporting Information (and are thus not included here). Again, at high levels of regional human capital and industry diversity the 95% confidence intervals of the predicted probabilities of being an entrepreneur do not overlap at high levels of entrepreneurial culture. Note that one could also allow the slope of the individual regression coefficients to vary across regions but this requires a huge amount of computational capacity in a data set with +200.000 observations, +10 individual level explanatory variables and +300 regions which considerably exceeded the capacity of our equipment.

Eighth, the two personality datasets are remarkably representative of the local population regarding population size, ethnicity, education, and employment, but they are less representative for age and gender. To address this potential bias we again computed the regional entrepreneurial culture indicators but this time we weighted the individual observations in the personality data set to match the age x gender distribution. Again the original effects were replicated (Table A17 in S1 Supporting Information).

Ninth and finally, start-up rates might not necessarily reflect high-impact entrepreneurship. To address this potential concern, we compare the regional distribution of the fastest growing firms in the US (Fortune 100) and GB (Fast Track 100). The majority of the results are reported in the Main paper. Here we present the analysis whether a region with above values in knowledge (human capital/industry diversity) and entrepreneurial culture have a higher likelihood of containing at least one of the Fortune 100 or Fast Track 100 firms then other regions. Tables A18 and A19 in S1 Supporting Information report the results for the US Fortune 100 firms. At least one Fortune 100 firm was located in around 22% of the regions exhibiting mean measures of knowledge and culture above the median. By contrast, at least one Fortune 100 firm was located in fewer than 4% of the regions exhibiting measures of knowledge and culture below the median. Chi2-tests confirm statistical significance of this difference (χ^2^ = 32.5, *p* < 0.001 for human capital and culture, χ^2^ = 22.0, *p* < 0.001 for industry diversity and culture). A similar pattern was observed for the data from Great Britain (Tables A20 and A21 in S1 Supporting Information). At least one Fast Track 100 firm was located in around one-fifth of the regions exhibiting high measures of knowledge and culture. By contrast, this held for fewer than 10% of the regions exhibiting low levels of knowledge and culture. Again this difference is statistical significant (χ^2^ = 7.6, *p* < 0.05 for human capital and culture, χ^2^ = 7.9, *p* < 0.05 for industry diversity and culture).

Note that this is again only correlational evidence. For example, there remains the possibility that these fast-growing firms were founded elsewhere and relocated to its contemporary location in search for example for qualified employees, venture capital or contacts to other entrepreneurial firms. While such relocation is not a typical behavior for firms we nevertheless made an effort to determine the region where the firm was founded. We contacted the high-growth firms and searched their web pages and additional sources. For the US sample, we were able to determine the original region for 25 of the 86 fast-growing firms. 21 out of those 25 fast-growing firms (84%) stayed in their region where they were founded. For GB, we were able to determine the origin for 33 of the 100 fast-growing firms. Again the vast majority (25 out of 33 firms = 85%) did not relocate but stayed in their region where they were founded. This additional analysis suggests that relocation of fast-growing firms does not drive our regression results and does not lead to spurious correlations.

1. **References**

1 Glaeser EL. Entrepreneurship and the city.2007. NBER Working Papers 13551.

2 Lee SY, Florida R, Acs Z. Creativity and entrepreneurship: a regional analysis of new firm formation. Reg Stud. 2004; 38(8): 879–891.

3 Coombes M, Bond S. Travel-to-Work-Areas: The 2007 Review. Technical Report, Office for National Statistics: 2007.

4 Mueller P, van Stel A, Storey D. The effect of new firm formation on regional development over time: The case of Great Britain, Small Bus Econ. 2008; 30(1): 59–71.

5 Parkinson M, Champion T, Simmie J, Turok I,Crookston M, Katz B et al. State of the English Cities. Technical Report ODPM: 2006, London.

6 Rentfrow PJ, Gosling, SD, Potter J. A theory of the emergence, persistence, and expression of geographic variation in psychological characteristics. Perspect Psychol Sci. 2008; 3(5): 339–369.

7 Loehlin JC, McCrae RR, Costa PT Jr, John OP. Heritabilities of common and measure-specific components of the Big Five personality factors. J Res Pers. 1998; 32(4): 431–453.

8 Obschonka M, Schmitt-Rodermund E, Silbereisen RK, Gosling SD, Potter J. The regional distribution and correlates of an entrepreneurship-prone personality profile in the United States, Germany and the United Kingdom: A socioecological perspective. J Pers Soc Psychol. 2013; 105(1): 104–122.

9 Fritsch M, Rusakova A. Personality traits, self-employment and professions. Jena: Economic Research Papers. 2010; 2010–2075.

10 Florida, R. L. The rise of the creative class. Revised paperback edition. New York: Basic Books: 2004.

11 McGranahan D, Wojan T. Recasting the creative class to examine growth processes in rural and urban communities. Reg Stud. 2006; 41(2), 197–216.

12 Boschma RA, Fritsch M. Creative class and regional growth: empirical evidence from seven european countries. Econ Geogr. 2009; 85(4): 391–423.

13 Norton EC, Wang H, Ai C. Computing interaction effects and standard errors in logit and probit models. The Stata Journal: 2004; 4(2): 154-167.

14 Snijders TAB, Bosker RJ. Multilevel analysis: an introduction to basic and advanced multilevel modeling. London: Sage Publications Ltd; 1999.

15 Wennberg K, Pathak S, Autio E. How culture moulds the effects of self-efficacy and fear of failure on entrepreneurship. Entrep Reg Dev.2013; 25(9-10): 756-780.

16 Kibler E. Formation of entrepreneurial intentions in a regional context. Entrep Reg Dev. 2013; 25(3-4): 293–323.

17 Stuetzer M, Obschonka M, Brixy U, Sternberg R, Cantner U. Regional characteristics, opportunity perception and entrepreneurial activities. Small Bus Econ. 2014; 42(2): 221–244.

**Table A1. Descriptive statistics and correlation matrix in US MSAs**

| Variable | | Mean | SD | (1) | (2) | (3) | (4) | (5) | (6) | (7) | (8) | (9) | (10) | (11) | (12) | (13) | (14) | (15) | (16) | (17) | (18) | (19) |
| --- | --- | --- | --- | --- | --- | --- | --- | --- | --- | --- | --- | --- | --- | --- | --- | --- | --- | --- | --- | --- | --- | --- |
| (1) | Entrepreneurship rate 2010 | 4.19 | 1.17 | 1.00 |  |  |  |  |  |  |  |  |  |  |  |  |  |  |  |  |  |  |
| (2) | Entrepreneurial culture | -20.29 | 0.38 | 0.36 | 1.00 |  |  |  |  |  |  |  |  |  |  |  |  |  |  |  |  |  |
| (3) | Agreeableness | 3.65 | 0.05 | -0.11 | -0.13 | 1.00 |  |  |  |  |  |  |  |  |  |  |  |  |  |  |  |  |
| (4) | Conscientiousness | 3.52 | 0.05 | -0.04 | 0.14 | 0.64 | 1.00 |  |  |  |  |  |  |  |  |  |  |  |  |  |  |  |
| (5) | Extraversion | 3.32 | 0.05 | -0.00 | 0.41 | 0.36 | 0.22 | 1.00 |  |  |  |  |  |  |  |  |  |  |  |  |  |  |
| (6) | Openness | 3.67 | 0.06 | 0.39 | 0.25 | -0.26 | -0.23 | -0.40 | 1.00 |  |  |  |  |  |  |  |  |  |  |  |  |  |
| (7) | Neuroticism | 3.00 | 0.05 | -0.20 | -0.61 | -0.49 | -0.36 | -0.33 | -0.00 | 1.00 |  |  |  |  |  |  |  |  |  |  |  |  |
| (8) | Human capital (2006-2010) | 25.36 | 7.81 | 0.28 | 0.36 | 0.02 | -0.07 | -0.08 | 0.34 | -0.34 | 1.00 |  |  |  |  |  |  |  |  |  |  |  |
| (9) | Industry diversity (2006-2010) | 7.23 | 0.81 | 0.41 | 0.13 | -0.05 | 0.05 | 0.01 | 0.17 | -0.05 | -0.11 | 1.00 |  |  |  |  |  |  |  |  |  |  |
| (10) | Unemployment rate (mean 2006-2010) | 6.75 | 2.01 | -0.08 | -0.06 | -0.04 | -0.03 | -0.05 | 0.02 | 0.06 | -0.45 | 0.05 | 1.00 |  |  |  |  |  |  |  |  |  |
| (11) | Unemployment rate (delta 2006-2010) | 108.64 | 52.20 | 0.54 | 0.24 | -0.06 | 0.03 | 0.06 | 0.20 | -0.11 | 0.01 | 0.27 | 0.11 | 1.00 |  |  |  |  |  |  |  |  |
| (12) | Per capita income (mean 2006-2010) | 35454.58 | 6538.06 | 0.36 | 0.23 | -0.19 | -0.20 | -0.13 | 0.34 | -0.11 | 0.61 | 0.28 | -0.28 | 0.09 | 1.00 |  |  |  |  |  |  |  |
| (13) | Per capita income (delta 2006-2010) | 7.49 | 5.96 | -0.42 | -0.28 | 0.04 | 0.04 | 0.03 | -0.33 | 0.21 | -0.26 | -0.30 | -0.17 | -0.53 | -0.28 | 1.00 |  |  |  |  |  |  |
| (14) | Migration (2006-2010) | 0.57 | 0.45 | 0.10 | 0.25 | -0.10 | 0.03 | -0.11 | 0.18 | -0.15 | 0.30 | -0.12 | -0.06 | -0.01 | 0.16 | 0.13 | 1.00 |  |  |  |  |  |
| (15) | Age group 25-44 (2006-2010) | 25.96 | 2.13 | 0.02 | 0.17 | 0.02 | 0.04 | -0.01 | 0.16 | -0.15 | 0.24 | 0.29 | -0.08 | -0.14 | 0.24 | -0.13 | 0.29 | 1.00 |  |  |  |  |
| (16) | Population density (2010) | 288.83 | 326.82 | 0.15 | 0.13 | -0.10 | -0.17 | 0.01 | 0.25 | -0.01 | 0.31 | 0.20 | 0.05 | 0.12 | 0.51 | -0.22 | 0.13 | 0.26 | 1.00 |  |  |  |
| (17) | Population growth (2000-2010) | 11.35 | 10.81 | 0.51 | 0.33 | 0.01 | 0.07 | 0.07 | 0.16 | -0.26 | 0.11 | 0.25 | 0.02 | 0.42 | -0.09 | -0.25 | 0.22 | 0.23 | -0.12 | 1.00 |  |  |
| (18) | Region West | 0.22 | 0.41 | 0.29 | 0.20 | -0.09 | -0.12 | -0.28 | 0.36 | -0.23 | 0.08 | 0.23 | 0.21 | 0.19 | 0.06 | -0.27 | 0.14 | 0.09 | -0.08 | 0.28 | 1.00 |  |
| (19) | Region Midwest | 0.25 | 0.44 | -0.33 | -0.01 | 0.06 | 0.05 | 0.18 | -0.35 | -0.03 | 0.05 | -0.26 | 0.01 | -0.24 | -0.06 | 0.01 | -0.18 | -0.08 | -0.04 | -0.31 | -0.31 | 1.00 |
| (20) | Region Northeast | 0.12 | 0.33 | -0.10 | -0.15 | -0.18 | -0.27 | -0.08 | 0.02 | 0.24 | 0.15 | -0.18 | -0.09 | -0.15 | 0.23 | 0.21 | -0.03 | -0.15 | 0.28 | -0.27 | -0.20 | -0.22 |

Correlation coefficients above 0.10 are significant at the 5% level

**Table A2. Descriptive statistics and correlation matrix in GB LADs**

| Variable | | Mean | SD | (1) | (2) | (3) | (4) | (5) | (6) | (7) | (8) | (9) | (10) | (11) | (12) | (13) | (14) | (15) | (16) | (17) | (18) |
| --- | --- | --- | --- | --- | --- | --- | --- | --- | --- | --- | --- | --- | --- | --- | --- | --- | --- | --- | --- | --- | --- |
| (1) | Entrepreneurship rate 2011 | 9.82 | 2.94 | 1 |  |  |  |  |  |  |  |  |  |  |  |  |  |  |  |  |  |
| (2) | Entrepreneurial culture | -20.79 | 0.42 | 0.58 | 1 |  |  |  |  |  |  |  |  |  |  |  |  |  |  |  |  |
| (3) | Agreeableness | 3.75 | 0.03 | -0.20 | -0.16 | 1 |  |  |  |  |  |  |  |  |  |  |  |  |  |  |  |
| (4) | Conscientiousness | 3.66 | 0.05 | 0.23 | 0.38 | 0.39 | 1 |  |  |  |  |  |  |  |  |  |  |  |  |  |  |
| (5) | Extraversion | 3.23 | 0.04 | 0.49 | 0.71 | -0.06 | 0.10 | 1 |  |  |  |  |  |  |  |  |  |  |  |  |  |
| (6) | Openness | 3.65 | 0.05 | 0.19 | 0.44 | -0.08 | -0.22 | 0.22 | 1 |  |  |  |  |  |  |  |  |  |  |  |  |
| (7) | Neuroticism | 2.97 | 0.05 | -0.42 | -0.77 | -0.24 | -0.47 | -0.46 | -0.15 | 1 |  |  |  |  |  |  |  |  |  |  |  |
| (8) | Human capital (2011) | 30.48 | 8.23 | 0.45 | 0.51 | -0.03 | 0.05 | 0.40 | 0.40 | -0.40 | 1 |  |  |  |  |  |  |  |  |  |  |
| (9) | Industry diversity (2011) | 9.62 | 0.71 | 0.61 | 0.52 | -0.10 | 0.20 | 0.50 | 0.09 | -0.42 | 0.54 | 1 |  |  |  |  |  |  |  |  |  |
| (10) | Unemployment rate (mean 2007-2011) | 4.86 | 1.25 | -0.49 | -0.52 | -0.16 | -0.52 | -0.27 | -0.15 | 0.52 | -0.54 | -0.44 | 1 |  |  |  |  |  |  |  |  |
| (11) | Unemployment rate (delta 2007-2011) | 57.69 | 18.9 | -0.24 | -0.22 | 0.09 | 0.10 | -0.16 | -0.30 | 0.11 | -0.28 | -0.13 | 0.21 | 1 |  |  |  |  |  |  |  |
| (12) | Per capita income (mean 2007-2011) | 461.71 | 75.23 | 0.70 | 0.47 | -0.15 | 0.21 | 0.46 | 0.06 | -0.36 | 0.62 | 0.70 | -0.52 | -0.18 | 1 |  |  |  |  |  |  |
| (13) | Per capita income (delta 2007-2011) | 7.42 | 6.63 | 0.00 | 0.07 | 0.02 | 0.05 | 0.02 | 0.07 | -0.04 | 0.03 | 0.00 | -0.06 | -0.07 | 0.01 | 1 |  |  |  |  |  |
| (14) | Migration (2001) | 0.45 | 0.39 | 0.36 | 0.4 | -0.19 | -0.05 | 0.34 | 0.3 | -0.26 | 0.52 | 0.34 | -0.26 | -0.32 | 0.45 | 0.02 | 1 |  |  |  |  |
| (15) | Age group 25-44 (2011) | 25.23 | 2.97 | 0.01 | -0.08 | -0.34 | -0.36 | 0.07 | -0.09 | 0.18 | 0.09 | 0.13 | 0.36 | -0.03 | 0.18 | -0.07 | 0.42 | 1 |  |  |  |
| (16) | Population density (2011) | 938.42 | 1155.33 | -0.05 | -0.07 | -0.30 | -0.40 | 0.08 | 0.07 | 0.20 | -0.04 | 0.04 | 0.52 | -0.17 | -0.10 | -0.02 | 0.23 | 0.64 | 1 |  |  |
| (17) | Population growth (2001-2011) | 0.06 | 0.04 | 0.24 | 0.16 | -0.13 | 0.04 | 0.05 | 0.09 | -0.11 | 0.12 | 0.13 | -0.10 | -0.15 | 0.16 | 0.00 | 0.32 | 0.40 | 0.16 | 1 |  |
| (18) | Region England | 0.86 | 0.35 | 0.32 | 0.15 | -0.20 | 0.22 | 0.13 | -0.09 | -0.02 | -0.09 | 0.13 | -0.08 | -0.11 | 0.16 | -0.06 | 0.10 | 0.07 | 0.17 | 0.17 | 1 |
| (19) | Region Wales | 0.06 | 0.24 | -0.19 | -0.16 | 0.06 | -0.21 | -0.08 | 0.05 | 0.11 | -0.08 | -0.20 | 0.12 | -0.10 | -0.17 | -0.02 | -0.13 | -0.09 | -0.11 | -0.09 | -0.61 |

Correlation coefficients above 0.10 are significant at the 5% level

**Table A3. [Replication of Table 1 from Main paper with all control variables]: Entrepreneurship rate, human capital, industry diversity, entrepreneurial culture, and interactions**.

|  | Dependent variable: Entrepreneurship rate | | | | | | | | | | | | |
| --- | --- | --- | --- | --- | --- | --- | --- | --- | --- | --- | --- | --- | --- |
|  |  |  | US |  |  |  |  |  | GB |  |  |  |  |
| Variables | Model 1 |  | Model 2 |  | Model 3 |  | Model 4 |  | Model 5 |  | Model 6 |  |  |
| Human capital | 0.19 | * | 0.14 |  | 0.18 | * | -0.24 |  | -0.27 |  | -0.16 |  |  |
|  | (0.08) |  | (0.08) |  | (0.08) |  | (0.17) |  | (0.16) |  | (0.16) |  |  |
| Industry diversity | 0.34 | ** | 0.33 | ** | 0.21 | ** | 0.23 |  | 0.38 | ** | 0.25 |  |  |
|  | (0.06) |  | (0.06) |  | (0.06) |  | (0.14) |  | (0.14) |  | (0.13) |  |  |
| Entrepreneurial culture | 0.22 | ** | 0.20 | ** | 0.20 | ** | 0.92 | ** | 0.84 | ** | 0.90 | ** |  |
|  | (0.06) |  | (0.06) |  | (0.06) |  | (0.16) |  | (0.16) |  | (0.15) |  |  |
| Interaction: Human capital X | ---- |  | 0.11 | * | ---- |  | ---- |  | 0.53 | ** | ---- |  |  |
| Entrepreneurial culture |  |  | (0.05) |  |  |  |  |  | (0.11) |  |  |  |  |
| Interaction: Industry diversity X | ---- |  | ---- |  | 0.19 | ** | ---- |  | ---- |  | 0.54 | ** |  |
| Entrepreneurial culture |  |  |  |  | (0.05) |  |  |  |  |  | (0.10) |  |  |
| Unemployment rate (mean) | 0.02 |  | 0.03 |  | 0.02 |  | -0.04 |  | -0.17 |  | -0.14 |  |  |
|  | (0.07) |  | (0.07) |  | (0.06) |  | (0.17) |  | (0.17) |  | (0.16) |  |  |
| Unemployment rate (change) | 0.28 | ** | 0.28 | ** | 0.25 | ** | 0.14 |  | 0.12 |  | 0.18 |  |  |
|  | (0.05) |  | (0.05) |  | (0.05) |  | (0.11) |  | (0.11) |  | (0.11) |  |  |
| Per capita income (mean) | 0.09 |  | 0.11 |  | 0.10 |  | 2.12 | ** | 1.80 | ** | 1.81 | ** |  |
|  | (0.06) |  | (0.06) |  | (0.06) |  | (0.14) |  | (0.15) |  | (0.15) |  |  |
| Per capita income (change) | -0.12 |  | -0.13 |  | -0.12 |  | -0.14 |  | -0.12 |  | -0.08 |  |  |
|  | (0.07) |  | (0.07) |  | (0.07) |  | (0.11) |  | (0.11) |  | (0.11) |  |  |
| Migration | 0.14 | * | 0.14 | * | 0.12 |  | -0.01 |  | -0.13 |  | -0.05 |  |  |
|  | (0.06) |  | (0.06) |  | (0.06) |  | (0.15) |  | (0.15) |  | (0.14) |  |  |
| Age group 25-44 | -0.22 | ** | -0.23 | ** | -0.25 | ** | -0.65 | ** | -0.60 | ** | -0.61 | ** |  |
|  | (0.06) |  | (0.06) |  | (0.06) |  | (0.17) |  | (0.16) |  | (0.16) |  |  |
| Population density | 0.13 | ** | 0.14 | ** | 0.14 | ** | 0.91 | ** | 0.87 | ** | 0.93 | ** |  |
|  | (0.02) |  | (0.02) |  | (0.02) |  | (0.12) |  | (0.12) |  | (0.11) |  |  |
| Population growth | 0.23 | ** | 0.25 | ** | 0.26 | ** | 0.45 | ** | 0.52 | ** | 0.49 | ** |  |
|  | (0.06) |  | (0.06) |  | (0.06) |  | (0.11) |  | (0.11) |  | (0.10) |  |  |
| Region West | -0.22 | * | -0.23 | * | -0.24 | * | ---- |  | ---- |  | ---- |  |  |
|  | (0.10) |  | (0.10) |  | (0.10) |  |  |  |  |  |  |  |  |
| Region Midwest | -0.38 | ** | -0.36 | ** | -0.41 | ** | ---- |  | ---- |  | ---- |  |  |
|  | (0.11) |  | (0.11) |  | (0.11) |  |  |  |  |  |  |  |  |
| Region Northeast | 0.01 |  | 0.04 |  | -0.07 |  | ---- |  | ---- |  | ---- |  |  |
|  | (0.14) |  | (0.14) |  | (0.14) |  |  |  |  |  |  |  |  |
| Region England | ---- |  | ---- |  | ---- |  | 0.58 |  | 0.42 |  | 0.58 |  |  |
|  |  |  |  |  |  |  | (0.39) |  | (0.38) |  | (0.37) |  |  |
| Region Wales | ---- |  | ---- |  | ---- |  | 0.24 |  | 0.08 |  | 0.11 |  |  |
|  |  |  |  |  |  |  | (0.57) |  | (0.56) |  | (0.55) |  |  |
| Constant | 4.19 | ** | 4.17 | ** | 4.22 | ** | 9.15 | ** | 9.06 | ** | 8.94 | ** |  |
|  | (0.07) |  | (0.07) |  | (0.07) |  | (0.37) |  | (0.36) |  | (0.36) |  |  |
|  |  |  |  |  |  |  |  |  |  |  |  |  |  |
| Observations | 366 |  | 366 |  | 366 |  | 375 |  | 375 |  | 375 |  |  |
| Adjusted R^2^ | 0.630 |  | 0.634 |  | 0.646 |  | 0.846 |  | 0.855 |  | 0.858 |  |  |
| F test | 45.42 | ** | 43.08 | ** | 45.49 | ** | 159.2 | ** | 159.1 | ** | 162.6 | ** |  |
| AIC | 731.5 |  | 729 |  | 716 |  | 1477 |  | 1455 |  | 1448 |  |  |

The independent variables are industry diversity, human capital, and entrepreneurial culture based on current residence. The OLS regressions are weighted by the number of observations per region in the personality data file giving more weight to regions with more observations. Displaying unstandardized coefficients and standard errors in parentheses. **, * = 1%, 5% significance level.

**Table A4**: **Entrepreneurship rate, human capital, industry diversity, entrepreneurial culture, and interactions – unweighted OLS results**

|  | Dependent variable: Entrepreneurship rate | | | | | | | | | | | | |
| --- | --- | --- | --- | --- | --- | --- | --- | --- | --- | --- | --- | --- | --- |
|  |  |  | US |  |  |  |  |  | GB |  |  |  |  |
| Variables | Model 1 |  | Model 2 |  | Model 3 |  | Model 4 |  | Model 5 |  | Model 6 |  |  |
| Human capital | 0.15 | * | 0.15 | * | 0.15 | * | -0.16 |  | -0.15 |  | -0.07 |  |  |
|  | (0.07) |  | (0.07) |  | (0.07) |  | (0.15) |  | (0.14) |  | (0.14) |  |  |
| Industry diversity | 0.22 | ** | 0.22 | ** | 0.19 | ** | 0.38 | ** | 0.51 | ** | 0.53 | ** |  |
|  | (0.06) |  | (0.06) |  | (0.05) |  | (0.14) |  | (0.14) |  | (0.13) |  |  |
| Entrepreneurial culture | 0.09 | * | 0.10 | * | 0.12 | ** | 0.68 | ** | 0.73 | ** | 0.83 | ** |  |
|  | (0.05) |  | (0.05) |  | (0.05) |  | (0.13) |  | (0.12) |  | (0.12) |  |  |
| Interaction: Human capital X |  |  | 0.03 |  |  |  |  |  | 0.39 | ** |  |  |  |
| Entrepreneurial culture |  |  | (0.04) |  |  |  |  |  | (0.09) |  |  |  |  |
| Interaction: Industry diversity X |  |  |  |  | 0.17 | ** |  |  |  |  | 0.48 | ** |  |
| Entrepreneurial culture |  |  |  |  | (0.04) |  |  |  |  |  | (0.07) |  |  |
| Unemployment rate (mean) | -0.03 |  | -0.03 |  | -0.03 |  | -0.06 |  | -0.16 |  | -0.14 |  |  |
|  | (0.05) |  | (0.05) |  | (0.05) |  | (0.17) |  | (0.16) |  | (0.16) |  |  |
| Unemployment rate (change) | 0.21 | ** | 0.21 | ** | 0.19 | ** | -0.09 |  | -0.10 |  | -0.04 |  |  |
|  | (0.06) |  | (0.06) |  | (0.06) |  | (0.11) |  | (0.11) |  | (0.10) |  |  |
| Per capita income (mean) | 0.30 | ** | 0.30 | ** | 0.28 | ** | 1.53 | ** | 1.27 | ** | 1.15 | ** |  |
|  | (0.06) |  | (0.06) |  | (0.06) |  | (0.16) |  | (0.16) |  | (0.16) |  |  |
| Per capita income (change) | -0.07 |  | -0.07 |  | -0.07 |  | -0.08 |  | -0.04 |  | -0.02 |  |  |
|  | (0.06) |  | (0.06) |  | (0.06) |  | (0.09) |  | (0.09) |  | (0.09) |  |  |
| Migration | -0.02 |  | -0.02 |  | -0.03 |  | 0.04 |  | -0.06 |  | -0.05 |  |  |
|  | (0.05) |  | (0.05) |  | (0.05) |  | (0.13) |  | (0.13) |  | (0.12) |  |  |
| Age group 25-44 | -0.27 | ** | -0.27 | ** | -0.28 | ** | -0.57 | ** | -0.50 | ** | -0.51 | ** |  |
|  | (0.05) |  | (0.05) |  | (0.05) |  | (0.16) |  | (0.16) |  | (0.15) |  |  |
| Population density | 0.02 |  | 0.02 |  | 0.03 |  | 0.26 |  | 0.24 |  | 0.28 |  |  |
|  | (0.05) |  | (0.05) |  | (0.05) |  | (0.15) |  | (0.15) |  | (0.14) |  |  |
| Population growth | 0.40 | ** | 0.41 | ** | 0.40 | ** | 0.41 | ** | 0.43 | ** | 0.40 | ** |  |
|  | (0.06) |  | (0.06) |  | (0.05) |  | (0.11) |  | (0.11) |  | (0.10) |  |  |
| Region West | 0.07 |  | 0.06 |  | 0.02 |  | ---- |  | ---- |  | ---- |  |  |
|  | (0.12) |  | (0.12) |  | (0.11) |  |  |  |  |  |  |  |  |
| Region Midwest | -0.41 | ** | -0.40 | ** | -0.45 | ** | ---- |  | ---- |  | ---- |  |  |
|  | (0.12) |  | (0.12) |  | (0.11) |  |  |  |  |  |  |  |  |
| Region Northeast | -0.27 |  | -0.25 |  | -0.33 | * | ---- |  | ---- |  | ---- |  |  |
|  | (0.16) |  | (0.16) |  | (0.15) |  |  |  |  |  |  |  |  |
| Region England | ---- |  | ---- |  | ---- |  | 1.50 | ** | 1.40 | ** | 1.48 | ** |  |
|  |  |  |  |  |  |  | (0.37) |  | (0.37) |  | (0.35) |  |  |
| Region Wales | ---- |  | ---- |  | ---- |  | 0.79 |  | 0.69 |  | 0.65 |  |  |
|  |  |  |  |  |  |  | (0.53) |  | (0.51) |  | (0.50) |  |  |
| Constant | 4.31 | ** | 4.30 | ** | 4.32 | ** | 8.49 | ** | 8.38 | ** | 8.27 | ** |  |
|  | (0.07) |  | (0.07) |  | (0.07) |  | (0.35) |  | (0.34) |  | (0.33) |  |  |
|  |  |  |  |  |  |  |  |  |  |  |  |  |  |
| Observations | 366 |  | 366 |  | 366 |  | 375 |  | 375 |  | 375 |  |  |
| Adjusted R^2^ | 0.581 |  | 0.580 |  | 0.598 |  | 0.635 |  | 0.654 |  | 0.675 |  |  |
| F test | 37.12 | ** | 34.61 | ** | 37.25 | ** | 51.02 | ** | 51.47 | ** | 56.51 | ** |  |
| AIC | 850.7 |  | 852.3 |  | 836 |  | 1508 |  | 1489 |  | 1466 |  |  |

The independent variables are industry diversity, human capital, and entrepreneurial culture based on current residence. Unweighted OLS regressions. Displaying unstandardized coefficients and standard errors in parentheses. **, * = 1%, 5% significance level.

**Table A5. Entrepreneurship, human capital, industry diversity, alternative entrepreneurial culture measure, and interactions.**

|  | Dependent variable: Entrepreneurship rate | | | | | | | | | | | | |
| --- | --- | --- | --- | --- | --- | --- | --- | --- | --- | --- | --- | --- | --- |
|  | |  |  | US |  |  |  |  |  | GB |  |  |  |
| Variables | | Model 1 |  | Model 2 |  | Model 3 |  | Model 4 |  | Model 5 |  | Model 6 |  |
| Human capital | | 0.17 | * | 0.13 |  | 0.15 |  | -0.25 |  | -0.30 |  | -0.19 |  |
|  | | (0.08) |  | (0.08) |  | (0.08) |  | (0.17) |  | (0.16) |  | (0.16) |  |
| Industry diversity | | 0.33 | ** | 0.32 | ** | 0.22 | ** | 0.28 | * | 0.44 | ** | 0.31 | * |
|  | | (0.06) |  | (0.06) |  | (0.06) |  | (0.14) |  | (0.14) |  | (0.13) |  |
| Entrepreneurial culture | | 0.25 | ** | 0.21 | ** | 0.23 | ** | 0.86 | ** | 0.77 | ** | 0.84 | ** |
|  | | (0.06) |  | (0.06) |  | (0.06) |  | (0.16) |  | (0.16) |  | (0.15) |  |
| Interaction: Human capital X | | ---- |  | 0.09 |  | ---- |  | ---- |  | 0.48 | ** | ---- |  |
| Entrepreneurial culture | |  |  | (0.05) |  |  |  |  |  | (0.11) |  |  |  |
| Interaction: Industry diversity X | | ---- |  | ---- |  | 0.15 | ** | ---- |  | ---- |  | 0.56 | ** |
| Entrepreneurial culture | |  |  |  |  | (0.04) |  |  |  |  |  | (0.09) |  |
| Unemployment rate (mean) | | 0.02 |  | 0.02 |  | 0.01 |  | -0.04 |  | -0.18 |  | -0.18 |  |
|  | | (0.07) |  | (0.06) |  | (0.06) |  | (0.17) |  | (0.17) |  | (0.17) |  |
| Unemployment rate (change) | | 0.28 | ** | 0.29 | ** | 0.26 | ** | 0.13 |  | 0.12 |  | 0.17 |  |
|  | | (0.05) |  | (0.05) |  | (0.05) |  | (0.11) |  | (0.11) |  | (0.11) |  |
| Per capita income (mean) | | 0.11 |  | 0.13 | * | 0.13 | * | 2.10 | ** | 1.83 | ** | 1.77 | ** |
|  | | (0.06) |  | (0.06) |  | (0.06) |  | (0.14) |  | (0.15) |  | (0.15) |  |
| Per capita income (change) | | -0.11 |  | -0.11 |  | -0.10 |  | -0.15 |  | -0.14 |  | -0.08 |  |
|  | | (0.07) |  | (0.07) |  | (0.07) |  | (0.11) |  | (0.11) |  | (0.11) |  |
| Migration | | 0.14 | * | 0.15 | * | 0.12 | * | -0.00 |  | -0.10 |  | -0.02 |  |
|  | | (0.06) |  | (0.06) |  | (0.06) |  | (0.15) |  | (0.15) |  | (0.14) |  |
| Age group 25-44 | | -0.23 | ** | -0.23 | ** | -0.24 | ** | -0.60 | ** | -0.57 | ** | -0.54 | ** |
|  | | (0.06) |  | (0.06) |  | (0.06) |  | (0.17) |  | (0.17) |  | (0.16) |  |
| Population density | | 0.13 | ** | 0.13 | ** | 0.14 | ** | 0.92 | ** | 0.92 | ** | 0.93 | ** |
|  | | (0.02) |  | (0.02) |  | (0.02) |  | (0.12) |  | (0.12) |  | (0.11) |  |
| Population growth | | 0.25 | ** | 0.26 | ** | 0.27 | ** | 0.44 | ** | 0.51 | ** | 0.47 | ** |
|  | | (0.06) |  | (0.06) |  | (0.06) |  | (0.11) |  | (0.11) |  | (0.10) |  |
| Region West | | -0.16 |  | -0.16 |  | -0.14 |  | ---- |  | ---- |  | ---- |  |
|  | | (0.10) |  | (0.10) |  | (0.10) |  |  |  |  |  |  |  |
| Region Midwest | | -0.29 | ** | -0.28 | * | -0.29 | ** | ---- |  | ---- |  | ---- |  |
|  | | (0.11) |  | (0.11) |  | (0.11) |  |  |  |  |  |  |  |
| Region Northeast | | 0.08 |  | 0.11 |  | 0.03 |  | ---- |  | ---- |  | ---- |  |
|  | | (0.14) |  | (0.14) |  | (0.14) |  |  |  |  |  |  |  |
| Region England | | ---- |  | ---- |  | ---- |  | 0.55 |  | 0.36 |  | 0.55 |  |
|  | |  |  |  |  |  |  | (0.39) |  | (0.39) |  | (0.38) |  |
| Region Wales | | ---- |  | ---- |  | ---- |  | 0.23 |  | 0.10 |  | 0.12 |  |
|  | |  |  |  |  |  |  | (0.58) |  | (0.56) |  | (0.55) |  |
| Constant | | 4.15 | ** | 4.13 | ** | 4.14 | ** | 9.17 | ** | 9.14 | ** | 8.97 | ** |
|  | | (0.07) |  | (0.07) |  | (0.07) |  | (0.37) |  | (0.37) |  | (0.36) |  |
|  | |  |  |  |  |  |  |  |  |  |  |  |  |
| Observations | | 366 |  | 366 |  | 366 |  | 375 |  | 375 |  | 375 |  |
| Adjusted R^2^ | | 0.636 |  | 0.638 |  | 0.649 |  | 0.845 |  | 0.852 |  | 0.859 |  |
| F test | | 46.51 | ** | 43.96 | ** | 45.93 | ** | 157.6 | ** | 154.7 | ** | 163.2 | ** |
| AIC | | 725.9 |  | 724.2 |  | 713.6 |  | 1481 |  | 1464 |  | 1447 |  |

The independent variables are industry diversity, human capital, and entrepreneurial culture based on current residence. The entrepreneurial culture measure is based on a linear composite by adding means of openness, conscientiousness, and extraversion, and then substracting means scores of neuroticism and agreeableness. The OLS regressions are weighted by the number of observations per region in the personality data file giving more weight to regions with more observations. Displaying unstandardized coefficients and standard errors in parentheses. **, * = 1%, 5% significance level.

**Table A6. Entrepreneurship, human capital, industry diversity, neutral profile 1, and interactions.**

|  | Dependent variable: Entrepreneurship rate | | | | | | | | | | | | |
| --- | --- | --- | --- | --- | --- | --- | --- | --- | --- | --- | --- | --- | --- |
|  | |  |  | US |  |  |  |  |  | GB |  |  |  |
| Variables | | Model 1 |  | Model 2 |  | Model 3 |  | Model 4 |  | Model 5 |  | Model 6 |  |
| Human capital | | 0.25 | ** | 0.25 | ** | 0.25 | ** | -0.08 |  | -0.10 |  | -0.13 |  |
|  | | (0.08) |  | (0.08) |  | (0.08) |  | (0.17) |  | (0.17) |  | (0.16) |  |
| Industry diversity | | 0.38 | ** | 0.38 | ** | 0.38 | ** | 0.43 | ** | 0.46 | ** | 0.47 | ** |
|  | | (0.06) |  | (0.06) |  | (0.06) |  | (0.14) |  | (0.15) |  | (0.14) |  |
| Neutral profile 1 | | -0.07 |  | -0.06 |  | -0.07 |  | -0.18 |  | -0.19 |  | -0.21 |  |
|  | | (0.05) |  | (0.06) |  | (0.05) |  | (0.13) |  | (0.13) |  | (0.13) |  |
| Interaction: Human capital X | | ---- |  | -0.05 |  | ---- |  | ---- |  | -0.08 |  | ---- |  |
| Neutral profile 1 | |  |  | (0.06) |  |  |  |  |  | (0.12) |  |  |  |
| Interaction: Industry diversity X | | ---- |  | ---- |  | -0.00 |  | ---- |  |  |  | -0.56 | ** |
| Neutral profile 1 | |  |  |  |  | (0.04) |  |  |  |  |  | (0.11) |  |
| Unemployment rate (mean) | | 0.01 |  | 0.01 |  | 0.01 |  | -0.12 |  | -0.15 |  | -0.28 |  |
|  | | (0.07) |  | (0.07) |  | (0.07) |  | (0.18) |  | (0.18) |  | (0.18) |  |
| Unemployment rate (change) | | 0.31 | ** | 0.31 | ** | 0.31 | ** | 0.33 |  | 0.34 |  | 0.32 |  |
|  | | (0.05) |  | (0.05) |  | (0.05) |  | (0.21) |  | (0.21) |  | (0.21) |  |
| Per capita income (mean) | | 0.07 |  | 0.07 |  | 0.07 |  | 2.17 | ** | 2.15 | ** | 1.98 | ** |
|  | | (0.06) |  | (0.06) |  | (0.06) |  | (0.14) |  | (0.15) |  | (0.14) |  |
| Per capita income (change) | | -0.13 |  | -0.12 |  | -0.13 |  | -0.14 |  | -0.14 |  | -0.10 |  |
|  | | (0.07) |  | (0.07) |  | (0.07) |  | (0.11) |  | (0.11) |  | (0.11) |  |
| Migration | | 0.19 | ** | 0.19 | ** | 0.19 | ** | 0.14 |  | 0.14 |  | 0.22 |  |
|  | | (0.06) |  | (0.06) |  | (0.06) |  | (0.15) |  | (0.15) |  | (0.14) |  |
| Age group 25-44 | | -0.26 | ** | -0.25 | ** | -0.26 | ** | -0.83 | ** | -0.82 | ** | -0.69 | ** |
|  | | (0.06) |  | (0.06) |  | (0.06) |  | (0.18) |  | (0.18) |  | (0.17) |  |
| Population density | | 0.15 | ** | 0.14 | ** | 0.15 | ** | 1.06 | ** | 1.07 | ** | 1.05 | ** |
|  | | (0.02) |  | (0.03) |  | (0.02) |  | (0.12) |  | (0.12) |  | (0.12) |  |
| Population growth | | 0.29 | ** | 0.28 | ** | 0.29 | ** | 0.51 | ** | 0.51 | ** | 0.49 | ** |
|  | | (0.06) |  | (0.06) |  | (0.06) |  | (0.11) |  | (0.11) |  | (0.11) |  |
| Region West | | -0.17 |  | -0.16 |  | -0.17 |  | ---- |  | ---- |  | ---- |  |
|  | | (0.10) |  | (0.10) |  | (0.11) |  |  |  |  |  |  |  |
| Region Midwest | | -0.27 | * | -0.26 | * | -0.27 | * | ---- |  | ---- |  | ---- |  |
|  | | (0.12) |  | (0.12) |  | (0.12) |  |  |  |  |  |  |  |
| Region Northeast | | -0.01 |  | -0.01 |  | -0.01 |  | ---- |  | ---- |  | ---- |  |
|  | | (0.14) |  | (0.14) |  | (0.14) |  |  |  |  |  |  |  |
| Region England | | ---- |  | ---- |  | ---- |  | 0.92 | * | 0.86 | * | 0.85 | * |
|  | |  |  |  |  |  |  | (0.40) |  | (0.41) |  | (0.39) |  |
| Region Wales | | ---- |  | ---- |  | ---- |  | 0.53 |  | 0.51 |  | 0.52 |  |
|  | |  |  |  |  |  |  | (0.60) |  | (0.60) |  | (0.58) |  |
| Constant | | 4.18 | ** | 4.18 | ** | 4.18 | ** | 8.77 | ** | 8.81 | ** | 8.78 | ** |
|  | | (0.07) |  | (0.07) |  | (0.08) |  | (0.38) |  | (0.39) |  | (0.37) |  |
|  | |  |  |  |  |  |  |  |  |  |  |  |  |
| Observations | | 366 |  | 366 |  | 366 |  | 375 |  | 375 |  | 375 |  |
| Adjusted R^2^ | | 0.619 |  | 0.619 |  | 0.618 |  | 0.833 |  | 0.833 |  | 0.844 |  |
| F test | | 43.29 | ** | 40.45 | ** | 40.29 | ** | 144.6 | ** | 134.1 | ** | 145.7 | ** |
| AIC | | 742.7 |  | 743.8 |  | 744.7 |  | 1508 |  | 1509 |  | 1483 |  |

The independent variables are industry diversity, human capital, and entrepreneurial culture based on current residence. Instead of the entrepreneurial constellation of the Big Five traits, a neutral profile is computed using each individual’s deviations from the middle points in the Big Five Likert scales. The OLS regressions are weighted by the number of observations per region in the personality data file giving more weight to regions with more observations. Displaying unstandardized coefficients and standard errors in parentheses. **, * = 1%, 5% significance level.

**Table A7. Entrepreneurship, human capital, industry diversity, neutral profile 2, and interactions.**

|  | Dependent variable: Entrepreneurship rate | | | | | | | | | | | | |
| --- | --- | --- | --- | --- | --- | --- | --- | --- | --- | --- | --- | --- | --- |
|  | |  |  | US |  |  |  |  |  | GB |  |  |  |
| Variables | | Model 1 |  | Model 2 |  | Model 3 |  | Model 4 |  | Model 5 |  | Model 6 |  |
| Human capital | | 0.26 | ** | 0.27 | ** | 0.27 | ** | -0.05 |  | -0.01 |  | -0.07 |  |
|  | | (0.08) |  | (0.08) |  | (0.08) |  | (0.17) |  | (0.17) |  | (0.16) |  |
| Industry diversity | | 0.39 | ** | 0.39 | ** | 0.36 | ** | 0.41 | ** | 0.39 | ** | 0.40 | ** |
|  | | (0.06) |  | (0.06) |  | (0.06) |  | (0.14) |  | (0.14) |  | (0.14) |  |
| Neutral profile 2 | | -0.03 |  | -0.02 |  | -0.01 |  | 0.20 |  | 0.21 |  | 0.18 |  |
|  | | (0.06) |  | (0.06) |  | (0.06) |  | (0.13) |  | (0.13) |  | (0.13) |  |
| Interaction: Human capital X | | ---- |  | -0.03 |  | ---- |  | ---- |  | 0.22 |  | ---- |  |
| Neutral profile 2 | |  |  | (0.06) |  |  |  |  |  | (0.13) |  |  |  |
| Interaction: Industry diversity X | | ---- |  | ---- |  | 0.10 |  | ---- |  |  |  | -0.25 | * |
| Neutral profile 2 | |  |  |  |  | (0.05) |  |  |  |  |  | (0.12) |  |
| Unemployment rate (mean) | | 0.02 |  | 0.02 |  | 0.02 |  | -0.11 |  | -0.08 |  | -0.13 |  |
|  | | (0.07) |  | (0.07) |  | (0.07) |  | (0.18) |  | (0.18) |  | (0.18) |  |
| Unemployment rate (change) | | 0.31 | ** | 0.31 | ** | 0.29 | ** | 0.38 |  | 0.37 |  | 0.37 |  |
|  | | (0.05) |  | (0.05) |  | (0.06) |  | (0.21) |  | (0.21) |  | (0.21) |  |
| Per capita income (mean) | | 0.06 |  | 0.05 |  | 0.05 |  | 2.22 | ** | 2.24 | ** | 2.20 | ** |
|  | | (0.06) |  | (0.06) |  | (0.06) |  | (0.14) |  | (0.14) |  | (0.14) |  |
| Per capita income (change) | | -0.13 |  | -0.13 |  | -0.13 |  | -0.13 |  | -0.13 |  | -0.12 |  |
|  | | (0.07) |  | (0.07) |  | (0.07) |  | (0.11) |  | (0.11) |  | (0.11) |  |
| Migration | | 0.19 | ** | 0.19 | ** | 0.19 | ** | 0.17 |  | 0.15 |  | 0.18 |  |
|  | | (0.06) |  | (0.06) |  | (0.06) |  | (0.15) |  | (0.15) |  | (0.15) |  |
| Age group 25-44 | | -0.25 | ** | -0.25 | ** | -0.27 | ** | -0.91 | ** | -0.91 | ** | -0.88 | ** |
|  | | (0.06) |  | (0.06) |  | (0.06) |  | (0.17) |  | (0.17) |  | (0.17) |  |
| Population density | | 0.15 | ** | 0.15 | ** | 0.15 | ** | 1.06 | ** | 1.06 | ** | 1.06 | ** |
|  | | (0.03) |  | (0.03) |  | (0.03) |  | (0.12) |  | (0.12) |  | (0.12) |  |
| Population growth | | 0.28 | ** | 0.28 | ** | 0.29 | ** | 0.51 | ** | 0.52 | ** | 0.50 | ** |
|  | | (0.06) |  | (0.06) |  | (0.06) |  | (0.11) |  | (0.11) |  | (0.11) |  |
| Region West | | -0.20 |  | -0.19 |  | -0.24 | * | ---- |  | ---- |  | ---- |  |
|  | | (0.10) |  | (0.10) |  | (0.10) |  |  |  |  |  |  |  |
| Region Midwest | | -0.31 | ** | -0.30 | * | -0.36 | ** | ---- |  | ---- |  | ---- |  |
|  | | (0.12) |  | (0.12) |  | (0.12) |  |  |  |  |  |  |  |
| Region Northeast | | -0.05 |  | -0.05 |  | -0.09 |  | ---- |  | ---- |  | ---- |  |
|  | | (0.14) |  | (0.14) |  | (0.14) |  |  |  |  |  |  |  |
| Region England | | ---- |  | ---- |  | ---- |  | 1.10 | ** | 1.22 | ** | 1.06 | ** |
|  | |  |  |  |  |  |  | (0.40) |  | (0.41) |  | (0.40) |  |
| Region Wales | | ---- |  | ---- |  | ---- |  | 0.66 |  | 0.74 |  | 0.64 |  |
|  | |  |  |  |  |  |  | (0.60) |  | (0.60) |  | (0.59) |  |
| Constant | | 4.20 | ** | 4.20 | ** | 4.24 | ** | 8.62 | ** | 8.49 | ** | 8.66 | ** |
|  | | (0.07) |  | (0.07) |  | (0.08) |  | (0.38) |  | (0.39) |  | (0.38) |  |
|  | |  |  |  |  |  |  |  |  |  |  |  |  |
| Observations | | 366 |  | 366 |  | 366 |  | 375 |  | 375 |  | 375 |  |
| Adjusted R^2^ | | 0.617 |  | 0.616 |  | 0.619 |  | 0.833 |  | 0.834 |  | 0.835 |  |
| F test | | 42.94 | ** | 40.02 | ** | 40.62 | ** | 144.9 | ** | 135.5 | ** | 135.9 | ** |
| AIC | | 744.6 |  | 746.3 |  | 742.9 |  | 1507 |  | 1506 |  | 1505 |  |

The independent variables are industry diversity, human capital, and entrepreneurial culture based on current residence. Instead of the entrepreneurial constellation of the Big Five traits, a neutral profile is computed using each individual’s deviations from the mean values of the Big Five traits. The OLS regressions are weighted by the number of observations per region in the personality data file giving more weight to regions with more observations. Displaying unstandardized coefficients and standard errors in parentheses. **, * = 1%, 5% significance level.

**Table A8. Entrepreneurship rate, industry diversity, single Big Five Traits, and interaction.**

|  | Dependent variable: Entrepreneurship rate | | | | | | | | | | | |
| --- | --- | --- | --- | --- | --- | --- | --- | --- | --- | --- | --- | --- |
|  |  |  | US |  |  |  |  |  | GB |  |  |  |
| Variables | Model 1 |  | Model 2 |  | Model 3 |  | Model 4 |  | Model 5 |  | Model 6 |  |
| Human capital | 0.18 | * | 0.14 |  | 0.17 | * | -0.48 | ** | -0.46 | * | -0.34 |  |
|  | (0.08) |  | (0.09) |  | (0.08) |  | (0.18) |  | (0.18) |  | (0.18) |  |
| Industry diversity | 0.33 | ** | 0.35 | ** | 0.21 | ** | 0.32 | * | 0.48 | ** | 0.26 |  |
|  | (0.06) |  | (0.06) |  | (0.07) |  | (0.15) |  | (0.15) |  | (0.14) |  |
| Agreeableness | -0.21 | ** | -0.21 | * | -0.19 | * | -0.30 | * | -0.34 | * | -0.29 | * |
|  | (0.08) |  | (0.08) |  | (0.08) |  | (0.14) |  | (0.14) |  | (0.13) |  |
| Conscientiousness | 0.15 | * | 0.14 |  | 0.16 | * | 0.19 |  | 0.21 |  | 0.25 |  |
|  | (0.07) |  | (0.07) |  | (0.07) |  | (0.17) |  | (0.16) |  | (0.16) |  |
| Extraversion | 0.14 | * | 0.13 |  | 0.12 |  | 0.34 | * | 0.25 |  | 0.20 |  |
|  | (0.06) |  | (0.06) |  | (0.06) |  | (0.14) |  | (0.14) |  | (0.14) |  |
| Openness | 0.28 | ** | 0.26 | ** | 0.26 | ** | 0.65 | ** | 0.53 | ** | 0.46 | ** |
|  | (0.06) |  | (0.07) |  | (0.06) |  | (0.13) |  | (0.14) |  | (0.13) |  |
| Neuroticism | -0.06 |  | -0.07 |  | -0.03 |  | -0.17 |  | -0.15 |  | -0.20 |  |
|  | (0.07) |  | (0.08) |  | (0.07) |  | (0.17) |  | (0.17) |  | (0.17) |  |
| Int.: Bachelor X | ---- |  | -0.11 |  | ---- |  | ---- |  | -0.24 |  | ---- |  |
| Agreeableness |  |  | (0.08) |  |  |  |  |  | (0.12) |  |  |  |
| Int.: Bachelor X | ---- |  | -0.01 |  | ---- |  | ---- |  | 0.17 |  | ---- |  |
| Conscientiousness |  |  | (0.07) |  |  |  |  |  | (0.15) |  |  |  |
| Int.: Bachelor X | ---- |  | 0.03 |  | ---- |  | ---- |  | 0.34 | ** | ---- |  |
| Extraversion |  |  | (0.06) |  |  |  |  |  | (0.13) |  |  |  |
| Int.: Bachelor X | ---- |  | 0.06 |  | ---- |  | ---- |  | 0.14 |  | ---- |  |
| Openness |  |  | (0.05) |  |  |  |  |  | (0.10) |  |  |  |
| Int.: Bachelor X | ---- |  | -0.06 |  | ---- |  | ---- |  | -0.04 |  | ---- |  |
| Neuroticism |  |  | (0.06) |  |  |  |  |  | (0.16) |  |  |  |
| Int.: Industry diversity X | ---- |  | ---- |  | -0.13 |  | ---- |  | ---- |  | -0.11 |  |
| Agreeableness |  |  |  |  | (0.08) |  |  |  |  |  | (0.13) |  |
| Int.: Industry diversity X | ---- |  | ---- |  | 0.03 |  | ---- |  | ---- |  | 0.13 |  |
| Conscientiousness |  |  |  |  | (0.07) |  |  |  |  |  | (0.13) |  |
| Int.: Industry diversity X | ---- |  | ---- |  | 0.06 |  | ---- |  | ---- |  | 0.17 |  |
| Extraversion |  |  |  |  | (0.05) |  |  |  |  |  | (0.12) |  |
| Int.: Industry diversity X | ---- |  | ---- |  | 0.08 |  | ---- |  | ---- |  | 0.47 | ** |
| Openness |  |  |  |  | (0.04) |  |  |  |  |  | (0.09) |  |
| Int.: Industry diversity X | ---- |  | ---- |  | -0.15 | * | ---- |  | ---- |  | -0.04 |  |
| Neuroticism |  |  |  |  | (0.07) |  |  |  |  |  | (0.14) |  |
| Unemployment rate (mean) | 0.01 |  | -0.01 |  | -0.00 |  | -0.26 |  | -0.32 |  | -0.28 |  |
|  | (0.07) |  | (0.07) |  | (0.06) |  | (0.19) |  | (0.19) |  | (0.18) |  |
| Unemployment rate (change) | 0.28 | ** | 0.28 | ** | 0.26 | ** | 0.21 |  | 0.17 |  | 0.17 |  |
|  | (0.05) |  | (0.05) |  | (0.05) |  | (0.12) |  | (0.11) |  | (0.11) |  |
| Per capita income (mean) | 0.08 |  | 0.05 |  | 0.11 |  | 2.20 | ** | 1.86 | ** | 1.91 | ** |
|  | (0.06) |  | (0.07) |  | (0.06) |  | (0.15) |  | (0.17) |  | (0.15) |  |
| Per capita income (change) | -0.09 |  | -0.11 |  | -0.10 |  | -0.16 |  | -0.17 |  | -0.10 |  |
|  | (0.07) |  | (0.07) |  | (0.07) |  | (0.11) |  | (0.11) |  | (0.11) |  |
| Migration | 0.14 | * | 0.14 | * | 0.12 | * | -0.07 |  | -0.15 |  | 0.13 |  |
|  | (0.06) |  | (0.06) |  | (0.06) |  | (0.15) |  | (0.15) |  | (0.15) |  |
| Age group 25-44 | -0.25 | ** | -0.27 | ** | -0.27 | ** | -0.64 | ** | -0.64 | ** | -0.70 | ** |
|  | (0.06) |  | (0.06) |  | (0.06) |  | (0.18) |  | (0.18) |  | (0.17) |  |
| Population density | 0.11 | ** | 0.11 | ** | 0.12 | ** | 0.85 | ** | 0.78 | ** | 0.78 | ** |
|  | (0.03) |  | (0.03) |  | (0.03) |  | (0.12) |  | (0.12) |  | (0.11) |  |
| Population growth | 0.27 | ** | 0.29 | ** | 0.30 | ** | 0.45 | ** | 0.51 | ** | 0.46 | ** |
|  | (0.06) |  | (0.06) |  | (0.06) |  | (0.11) |  | (0.11) |  | (0.10) |  |
| Region West | -0.19 |  | -0.22 | * | -0.21 |  | ---- |  | ---- |  | ---- |  |
|  | (0.11) |  | (0.11) |  | (0.12) |  |  |  |  |  |  |  |
| Region Midwest | -0.19 |  | -0.12 |  | -0.20 |  | ---- |  | ---- |  | ---- |  |
|  | (0.12) |  | (0.12) |  | (0.12) |  |  |  |  |  |  |  |
| Region Northeast | 0.04 |  | 0.11 |  | 0.01 |  | ---- |  | ---- |  | ---- |  |
|  | (0.14) |  | (0.15) |  | (0.15) |  |  |  |  |  |  |  |
| Region England | ---- |  | ---- |  | ---- |  | 0.59 |  | 0.39 |  | 0.81 | * |
|  |  |  |  |  |  |  | (0.40) |  | (0.40) |  | (0.38) |  |
| Region Wales | ---- |  | ---- |  | ---- |  | 0.12 |  | -0.06 |  | 0.30 |  |
|  |  |  |  |  |  |  | (0.57) |  | (0.56) |  | (0.54) |  |
| Constant | 4.17 | ** | 4.13 | ** | 4.17 | ** | 9.14 | ** | 9.14 | ** | 8.84 | ** |
|  | (0.07) |  | (0.08) |  | (0.07) |  | (0.38) |  | (0.38) |  | (0.36) |  |
|  |  |  |  |  |  |  |  |  |  |  |  |  |
| Observations | 366 |  | 366 |  | 366 |  | 375 |  | 375 |  | 375 |  |
| Adjusted R^2^ | 0.644 |  | 0.645 |  | 0.653 |  | 0.849 |  | 0.856 |  | 0.867 |  |
| F | 37.69 | ** | 29.89 | ** | 30.81 | ** | 124.9 | ** | 102.4 | ** | 112.0 | ** |
| AIC | 721.3 |  | 724.5 |  | 717.1 |  | 1474 |  | 1460 |  | 1431 |  |

The independent variables are industry diversity, human capital and the single Big Five traits based on current residence. The OLS regressions are weighted by the number of observations per region in the personality data file giving more weight to regions with more observations. Displaying unstandardized coefficients and standard errors in parentheses. **, * = 1%, 5% significance level.

**Table A9. Entrepreneurship rate, human capital, industry diversity, entrepreneurial culture, and interactions for 56 English Cities**.

|  | Dependent variable: Entrepreneurship rate | | | | | |
| --- | --- | --- | --- | --- | --- | --- |
|  |  |  | GB |  |  |  |
| Variables | Model 1 |  | Model 2 |  | Model 3 |  |
| Human capital | -0.84 |  | -1.23 | ** | -0.64 |  |
|  | (0.50) |  | (0.38) |  | (0.34) |  |
| Industry diversity | 1.06 | * | 0.86 | * | 0.53 |  |
|  | (0.46) |  | (0.35) |  | (0.31) |  |
| Entrepreneurial culture | 2.72 | ** | 1.55 | ** | 1.08 | * |
|  | (0.53) |  | (0.44) |  | (0.41) |  |
| Interaction: Human capital X |  |  | 1.02 | ** |  |  |
| Entrepreneurial culture |  |  | (0.16) |  |  |  |
| Interaction: Industry diversity X |  |  |  |  | 1.02 | ** |
| Entrepreneurial culture |  |  |  |  | (0.13) |  |
| Constant | 9.27 | ** | 8.62 | ** | 8.77 | ** |
|  | (0.31) |  | (0.25) |  | (0.22) |  |
|  |  |  |  |  |  |  |
| Observations | 56 |  | 56 |  | 56 |  |
| Adjusted R^2^ | 0.840 |  | 0.909 |  | 0.928 |  |
| F test | 97.21 | ** | 137.6 | ** | 178.0 | ** |
| AIC | 230.9 |  | 200.5 |  | 187.1 |  |

The independent variables are industry diversity, human capital, and entrepreneurial culture based on current residence. The OLS regressions in Models 1-3 are weighted by the number of observations per region in the personality data file giving more weight to regions with more observations. Displaying unstandardized coefficients and standard errors in parentheses. **, * = 1%, 5% significance level.

**Table A10. Entrepreneurship rate, human capital, industry diversity, entrepreneurial culture based on youth residence, and interactions.**

|  | Dependent variable: Entrepreneurship rate | | | | | | | | | | | | |
| --- | --- | --- | --- | --- | --- | --- | --- | --- | --- | --- | --- | --- | --- |
|  |  |  | US |  |  |  |  |  | GB |  |  |  |  |
| Variables | Model 1 |  | Model 2 |  | Model 3 |  | Model 4 |  | Model 5 |  | Model 6 |  |  |
| Human capital | 0.22 | ** | 0.20 | * | 0.20 | * | -0.18 |  | -0.22 |  | -0.15 |  |  |
|  | (0.08) |  | (0.08) |  | (0.08) |  | (0.17) |  | (0.17) |  | (0.16) |  |  |
| Industry diversity | 0.30 | ** | 0.26 | ** | 0.20 | ** | 0.36 | * | 0.44 | ** | 0.36 | ** |  |
|  | (0.06) |  | (0.06) |  | (0.06) |  | (0.14) |  | (0.14) |  | (0.14) |  |  |
| Entrepreneurial culture | 0.26 | ** | 0.25 | ** | 0.21 | ** | 0.60 | ** | 0.56 | ** | 0.57 | ** |  |
|  | (0.06) |  | (0.06) |  | (0.06) |  | (0.13) |  | (0.13) |  | (0.13) |  |  |
| Interaction: Human capital X | ---- |  | 0.18 | ** | ---- |  | ---- |  | 0.37 | ** | ---- |  |  |
| Entrepreneurial culture |  |  | (0.06) |  |  |  |  |  | (0.11) |  |  |  |  |
| Interaction: Industry diversity X | ---- |  | ---- |  | 0.18 | ** | ---- |  | ---- |  | 0.46 | ** |  |
| Entrepreneurial culture |  |  |  |  | (0.05) |  |  |  |  |  | (0.11) |  |  |
| Unemployment rate (mean) | -0.00 |  | 0.00 |  | 0.00 |  | -0.16 |  | -0.24 |  | -0.25 |  |  |
|  | (0.06) |  | (0.06) |  | (0.06) |  | (0.17) |  | (0.17) |  | (0.17) |  |  |
| Unemployment rate (change) | 0.26 | ** | 0.26 | ** | 0.24 | ** | 0.19 |  | 0.19 |  | 0.21 |  |  |
|  | (0.05) |  | (0.05) |  | (0.05) |  | (0.12) |  | (0.11) |  | (0.11) |  |  |
| Per capita income (mean) | 0.04 |  | 0.05 |  | 0.06 |  | 2.16 | ** | 1.99 | ** | 1.96 | ** |  |
|  | (0.06) |  | (0.06) |  | (0.06) |  | (0.14) |  | (0.15) |  | (0.15) |  |  |
| Per capita income (change) | -0.14 | * | -0.15 | * | -0.15 | * | -0.12 |  | -0.10 |  | -0.07 |  |  |
|  | (0.07) |  | (0.07) |  | (0.07) |  | (0.11) |  | (0.11) |  | (0.11) |  |  |
| Migration | 0.20 | ** | 0.20 | ** | 0.18 | ** | 0.09 |  | 0.03 |  | 0.07 |  |  |
|  | (0.06) |  | (0.06) |  | (0.06) |  | (0.15) |  | (0.15) |  | (0.15) |  |  |
| Age group 25-44 | -0.23 | ** | -0.25 | ** | -0.24 | ** | -0.78 | ** | -0.75 | ** | -0.74 | ** |  |
|  | (0.06) |  | (0.06) |  | (0.06) |  | (0.17) |  | (0.17) |  | (0.16) |  |  |
| Population density | 0.15 | ** | 0.15 | ** | 0.15 | ** | 1.00 | ** | 0.97 | ** | 1.00 | ** |  |
|  | (0.02) |  | (0.02) |  | (0.02) |  | (0.12) |  | (0.12) |  | (0.12) |  |  |
| Population growth | 0.20 | ** | 0.21 | ** | 0.22 | ** | 0.50 | ** | 0.54 | ** | 0.52 | ** |  |
|  | (0.06) |  | (0.06) |  | (0.06) |  | (0.11) |  | (0.11) |  | (0.11) |  |  |
| Region West | -0.27 | ** | -0.31 | ** | -0.28 | ** | ---- |  | ---- |  | ---- |  |  |
|  | (0.10) |  | (0.10) |  | (0.10) |  |  |  |  |  |  |  |  |
| Region Midwest | -0.45 | ** | -0.50 | ** | -0.48 | ** | ---- |  | ---- |  | ---- |  |  |
|  | (0.11) |  | (0.11) |  | (0.11) |  |  |  |  |  |  |  |  |
| Region Northeast | -0.06 |  | -0.07 |  | -0.10 |  | ---- |  | ---- |  | ---- |  |  |
|  | (0.13) |  | (0.13) |  | (0.13) |  |  |  |  |  |  |  |  |
| Region England | ---- |  | ---- |  | ---- |  | 0.79 | * | 0.71 |  | 0.77 | * |  |
|  |  |  |  |  |  |  | (0.39) |  | (0.39) |  | (0.38) |  |  |
| Region Wales | ---- |  | ---- |  | ---- |  | 0.36 |  | 0.27 |  | 0.24 |  |  |
|  |  |  |  |  |  |  | (0.58) |  | (0.58) |  | (0.57) |  |  |
| Constant | 4.25 | ** | 4.25 | ** | 4.26 | ** | 8.96 | ** | 8.92 | ** | 8.87 | ** |  |
|  | (0.07) |  | (0.07) |  | (0.07) |  | (0.37) |  | (0.37) |  | (0.37) |  |  |
|  |  |  |  |  |  |  |  |  |  |  |  |  |  |
| Observations | 366 |  | 366 |  | 366 |  | 375 |  | 375 |  | 375 |  |  |
| Adjusted R^2^ | 0.640 |  | 0.649 |  | 0.652 |  | 0.841 |  | 0.845 |  | 0.848 |  |  |
| F test | 47.40 | ** | 46.08 | ** | 46.62 | ** | 153.2 | ** | 146.7 | ** | 149.5 | ** |  |
| AIC | 713.6 |  | 705.1 |  | 702.2 |  | 1490 |  | 1481 |  | 1475 |  |  |

The independent variables are industry diversity, human capital and entrepreneurial culture based on youth residence. The OLS regressions are weighted by the number of observations per region in the personality data file giving more weight to regions with more observations. Displaying unstandardized coefficients and standard errors in parentheses. **, * = 1%, 5% significance level.

**Table A11. Entrepreneurship rate, industry diversity, entrepreneurial culture (excluding all entrepreneurs in the personality data), and interactions in GB**.

|  | Dependent variable: Entrepreneurship rate | | | | | |
| --- | --- | --- | --- | --- | --- | --- |
|  |  |  | GB |  |  |  |
| Variables | Model 1 |  | Model 2 |  | Model 3 |  |
| Human capital | -0.22 |  | -0.27 |  | -0.13 |  |
|  | (0.17) |  | (0.16) |  | (0.16) |  |
| Industry diversity | 0.26 |  | 0.43 | ** | 0.27 | * |
|  | (0.14) |  | (0.14) |  | (0.13) |  |
| Entrepreneurial culture | 0.80 | ** | 0.76 | ** | 0.83 | ** |
|  | (0.16) |  | (0.15) |  | (0.15) |  |
| Interaction: Human capital X | ---- |  | 0.58 | ** | ---- |  |
| Entrepreneurial culture |  |  | (0.11) |  |  |  |
| Interaction: Industry diversity X | ---- |  | ---- |  | 0.55 | ** |
| Entrepreneurial culture |  |  |  |  | (0.10) |  |
| Unemployment rate (mean) | -0.06 |  | -0.19 |  | -0.14 |  |
|  | (0.17) |  | (0.17) |  | (0.17) |  |
| Unemployment rate (change) | 0.15 |  | 0.12 |  | 0.17 |  |
|  | (0.11) |  | (0.11) |  | (0.11) |  |
| Per capita income (mean) | 2.16 | ** | 1.84 | ** | 1.86 | ** |
|  | (0.14) |  | (0.15) |  | (0.15) |  |
| Per capita income (change) | -0.15 |  | -0.14 |  | -0.09 |  |
|  | (0.11) |  | (0.11) |  | (0.11) |  |
| Migration | 0.01 |  | -0.13 |  | -0.03 |  |
|  | (0.15) |  | (0.15) |  | (0.14) |  |
| Age group 25-44 | -0.69 | ** | -0.62 | ** | -0.66 | ** |
|  | (0.17) |  | (0.16) |  | (0.16) |  |
| Population density | 0.93 | ** | 0.88 | ** | 0.93 | ** |
|  | (0.12) |  | (0.12) |  | (0.11) |  |
| Population growth | 0.46 | ** | 0.53 | ** | 0.49 | ** |
|  | (0.11) |  | (0.11) |  | (0.11) |  |
| Region England | 0.64 |  | 0.48 |  | 0.65 |  |
|  | (0.39) |  | (0.38) |  | (0.38) |  |
| Region Wales | 0.30 |  | 0.15 |  | 0.17 |  |
|  | (0.58) |  | (0.56) |  | (0.56) |  |
| Constant | 9.09 | ** | 9.00 | ** | 8.88 | ** |
|  | (0.37) |  | (0.36) |  | (0.36) |  |
|  |  |  |  |  |  |  |
| Observations | 375 |  | 375 |  | 375 |  |
| Adjusted R^2^ | 0.843 |  | 0.854 |  | 0.856 |  |
| F test | 155.7 | ** | 157.0 | ** | 159.4 | ** |
| AIC | 1484 |  | 1459 |  | 1454 |  |

The independent variables are industry diversity, human capital and entrepreneurial culture based on current residence. The OLS regressions are weighted by the number of observations per region in the personality data file giving more weight to regions with more observations. Displaying unstandardized coefficients and standard errors in parentheses. **, * = 1%, 5% significance level.

**Table A12: Entrepreneurship rate, human capital, and different versions of the creative class**

|  | Dependent variable: US Entrepreneurship rate | | | | | | | | | | | | | |
| --- | --- | --- | --- | --- | --- | --- | --- | --- | --- | --- | --- | --- | --- | --- |
| Variables | Model 1 |  | Model 2 |  | Model 3 |  | Model 4 |  | Model 5 |  | Model 6 |  | Model 7 |  |
| Super creative core |  |  | -0.27 | ** |  |  |  |  |  |  |  |  |  |  |
| (Florida) |  |  | (0.06) |  |  |  |  |  |  |  |  |  |  |  |
| Creative professionals |  |  |  |  | -0.02 |  |  |  |  |  |  |  |  |  |
| (Florida) |  |  |  |  | (0.07) |  |  |  |  |  |  |  |  |  |
| Creative class |  |  |  |  |  |  | -0.20 | ** |  |  |  |  |  |  |
| (Florida) |  |  |  |  |  |  | (0.07) |  |  |  |  |  |  |  |
| Super creative core |  |  |  |  |  |  |  |  | -0.14 | * |  |  |  |  |
| (McGranahan) |  |  |  |  |  |  |  |  | (0.06) |  |  |  |  |  |
| Creative professionals |  |  |  |  |  |  |  |  |  |  | -0.02 |  |  |  |
| (McGranahan) |  |  |  |  |  |  |  |  |  |  | (0.07) |  |  |  |
| Creative class |  |  |  |  |  |  |  |  |  |  |  |  | -0.14 | + |
| (McGranahan) |  |  |  |  |  |  |  |  |  |  |  |  | (0.07) |  |
| Human capital | 0.19 | * | 0.39 | ** | 0.20 | * | 0.32 | ** | 0.30 | ** | 0.20 | * | 0.29 | ** |
|  | (0.08) |  | (0.09) |  | (0.08) |  | (0.09) |  | (0.09) |  | (0.08) |  | (0.09) |  |
| Industry diversity | 0.34 | ** | 0.37 | ** | 0.34 | ** | 0.38 | ** | 0.35 | ** | 0.35 | ** | 0.36 | ** |
|  | (0.06) |  | (0.06) |  | (0.06) |  | (0.06) |  | (0.06) |  | (0.06) |  | (0.06) |  |
| Entrepreneurial culture | 0.22 | ** | 0.19 | ** | 0.22 | ** | 0.21 | ** | 0.21 | ** | 0.22 | ** | 0.21 | ** |
|  | (0.06) |  | (0.06) |  | (0.06) |  | (0.06) |  | (0.06) |  | (0.06) |  | (0.06) |  |
| Unemployment rate (mean) | 0.02 |  | 0.10 |  | 0.03 |  | 0.06 |  | 0.04 |  | 0.03 |  | 0.04 |  |
|  | (0.07) |  | (0.07) |  | (0.07) |  | (0.07) |  | (0.07) |  | (0.07) |  | (0.07) |  |
| Unemployment rate (change) | 0.28 | ** | 0.27 | ** | 0.28 | ** | 0.28 | ** | 0.29 | ** | 0.28 | ** | 0.29 | ** |
|  | (0.05) |  | (0.05) |  | (0.05) |  | (0.05) |  | (0.05) |  | (0.05) |  | (0.05) |  |
| Per capita income (mean) | 0.09 |  | 0.11 | + | 0.09 |  | 0.12 | * | 0.11 | + | 0.09 |  | 0.11 | + |
|  | (0.06) |  | (0.06) |  | (0.06) |  | (0.06) |  | (0.06) |  | (0.06) |  | (0.06) |  |
| Per capita income (change) | -0.12 | + | -0.06 |  | -0.12 | + | -0.10 |  | -0.10 |  | -0.13 | + | -0.12 | + |
|  | (0.07) |  | (0.07) |  | (0.07) |  | (0.07) |  | (0.07) |  | (0.07) |  | (0.07) |  |
| Migration | 0.14 | * | 0.14 | * | 0.14 | * | 0.12 | + | 0.14 | * | 0.14 | * | 0.13 | * |
|  | (0.06) |  | (0.06) |  | (0.06) |  | (0.06) |  | (0.06) |  | (0.06) |  | (0.06) |  |
| Age group 25-44 | -0.22 | ** | -0.15 | * | -0.22 | ** | -0.17 | * | -0.18 | ** | -0.22 | ** | -0.18 | ** |
|  | (0.06) |  | (0.06) |  | (0.07) |  | (0.07) |  | (0.07) |  | (0.07) |  | (0.07) |  |
| Population density | 0.13 | ** | 0.13 | ** | 0.13 | ** | 0.13 | ** | 0.13 | ** | 0.13 | ** | 0.13 | ** |
|  | (0.02) |  | (0.02) |  | (0.02) |  | (0.02) |  | (0.02) |  | (0.02) |  | (0.02) |  |
| Population growth | 0.23 | ** | 0.22 | ** | 0.23 | ** | 0.21 | ** | 0.22 | ** | 0.23 | ** | 0.22 | ** |
|  | (0.06) |  | (0.06) |  | (0.06) |  | (0.06) |  | (0.06) |  | (0.06) |  | (0.06) |  |
| Region West | -0.22 | * | -0.17 | + | -0.23 | * | -0.26 | ** | -0.21 | * | -0.23 | * | -0.22 | * |
|  | (0.10) |  | (0.10) |  | (0.11) |  | (0.10) |  | (0.10) |  | (0.10) |  | (0.10) |  |
| Region Midwest | -0.38 | ** | -0.42 | ** | -0.38 | ** | -0.41 | ** | -0.40 | ** | -0.38 | ** | -0.40 | ** |
|  | (0.11) |  | (0.11) |  | (0.11) |  | (0.11) |  | (0.11) |  | (0.11) |  | (0.11) |  |
| Region Northeast | 0.01 |  | 0.03 |  | 0.01 |  | 0.02 |  | -0.02 |  | 0.01 |  | 0.00 |  |
|  | (0.14) |  | (0.14) |  | (0.14) |  | (0.14) |  | (0.14) |  | (0.14) |  | (0.14) |  |
| Constant | 4.19 | ** | 4.26 | ** | 4.20 | ** | 4.26 | ** | 4.23 | ** | 4.20 | ** | 4.23 | ** |
|  | (0.07) |  | (0.07) |  | (0.08) |  | (0.08) |  | (0.07) |  | (0.07) |  | (0.07) |  |
|  |  |  |  |  |  |  |  |  |  |  |  |  |  |  |
| Observations | 366 |  | 366 |  | 366 |  | 366 |  | 366 |  | 366 |  | 366 |  |
| Adjusted R2 | 0.630 |  | 0.647 |  | 0.629 |  | 0.637 |  | 0.634 |  | 0.629 |  | 0.633 |  |
| F test | 45.42 |  | 45.67 |  | 42.28 |  | 43.69 |  | 43.18 |  | 42.29 |  | 42.92 |  |
| AIC | 731.5 |  | 715 |  | 733.5 |  | 725.7 |  | 728.5 |  | 733.4 |  | 729.9 |  |

The OLS regressions are weighted by the number of observations per region in the personality data file giving more weight to regions with more observations. Displaying unstandardized coefficients and standard errors in parentheses. **, *, + = 1%, 5%, 10% significance level.

**Table A13: Entrepreneurship rate, human capital, interactions, and different versions of the creative class**

|  | Dependent variable: US Entrepreneurship rate | | | | | | | | | | | | | |
| --- | --- | --- | --- | --- | --- | --- | --- | --- | --- | --- | --- | --- | --- | --- |
| Variables | Model 1 |  | Model 2 |  | Model 3 |  | Model 4 |  | Model 5 |  | Model 6 |  | Model 7 |  |
| Super creative core |  |  | -0.26 | ** |  |  |  |  |  |  |  |  |  |  |
| (Florida) |  |  | (0.06) |  |  |  |  |  |  |  |  |  |  |  |
| Creative professionals |  |  |  |  | 0.01 |  |  |  |  |  |  |  |  |  |
| (Florida) |  |  |  |  | (0.07) |  |  |  |  |  |  |  |  |  |
| Creative class |  |  |  |  |  |  | -0.18 | * |  |  |  |  |  |  |
| (Florida) |  |  |  |  |  |  | (0.07) |  |  |  |  |  |  |  |
| Super creative core |  |  |  |  |  |  |  |  | -0.13 | * |  |  |  |  |
| (McGranahan) |  |  |  |  |  |  |  |  | (0.06) |  |  |  |  |  |
| Creative professionals |  |  |  |  |  |  |  |  |  |  | -0.00 |  |  |  |
| (McGranahan) |  |  |  |  |  |  |  |  |  |  | (0.07) |  |  |  |
| Creative class |  |  |  |  |  |  |  |  |  |  |  |  | -0.12 | + |
| (McGranahan) |  |  |  |  |  |  |  |  |  |  |  |  | (0.07) |  |
| Human capital | 0.14 | + | 0.34 | ** | 0.14 |  | 0.26 | ** | 0.25 | * | 0.14 |  | 0.23 | * |
|  | (0.08) |  | (0.09) |  | (0.09) |  | (0.09) |  | (0.10) |  | (0.09) |  | (0.10) |  |
| Industry diversity | 0.33 | ** | 0.37 | ** | 0.33 | ** | 0.37 | ** | 0.35 | ** | 0.33 | ** | 0.36 | ** |
|  | (0.06) |  | (0.06) |  | (0.06) |  | (0.06) |  | (0.06) |  | (0.06) |  | (0.06) |  |
| Entrepreneurial culture | 0.20 | ** | 0.17 | ** | 0.19 | ** | 0.19 | ** | 0.19 | ** | 0.19 | ** | 0.19 | ** |
|  | (0.06) |  | (0.06) |  | (0.06) |  | (0.06) |  | (0.06) |  | (0.06) |  | (0.06) |  |
| Interaction: Human capital X | 0.11 | * | 0.10 | + | 0.11 | * | 0.10 | + | 0.11 | + | 0.11 | * | 0.10 | + |
| Entrepreneurial culture | (0.05) |  | (0.05) |  | (0.06) |  | (0.05) |  | (0.05) |  | (0.06) |  | (0.06) |  |
| Unemployment rate (mean) | 0.03 |  | 0.10 |  | 0.03 |  | 0.06 |  | 0.04 |  | 0.03 |  | 0.04 |  |
|  | (0.07) |  | (0.07) |  | (0.07) |  | (0.07) |  | (0.07) |  | (0.07) |  | (0.07) |  |
| Unemployment rate (change) | 0.28 | ** | 0.27 | ** | 0.28 | ** | 0.28 | ** | 0.29 | ** | 0.28 | ** | 0.29 | ** |
|  | (0.05) |  | (0.05) |  | (0.05) |  | (0.05) |  | (0.05) |  | (0.05) |  | (0.05) |  |
| Per capita income (mean) | 0.11 | + | 0.13 | * | 0.10 |  | 0.14 | * | 0.13 | * | 0.11 | + | 0.13 | * |
|  | (0.06) |  | (0.06) |  | (0.06) |  | (0.06) |  | (0.06) |  | (0.06) |  | (0.06) |  |
| Per capita income (change) | -0.13 | + | -0.06 |  | -0.13 | + | -0.11 |  | -0.11 |  | -0.13 | + | -0.12 | + |
|  | (0.07) |  | (0.07) |  | (0.07) |  | (0.07) |  | (0.07) |  | (0.07) |  | (0.07) |  |
| Migration | 0.14 | * | 0.14 | * | 0.14 | * | 0.12 | * | 0.14 | * | 0.14 | * | 0.14 | * |
|  | (0.06) |  | (0.06) |  | (0.06) |  | (0.06) |  | (0.06) |  | (0.06) |  | (0.06) |  |
| Age group 25-44 | -0.23 | ** | -0.16 | * | -0.23 | ** | -0.18 | ** | -0.19 | ** | -0.23 | ** | -0.19 | ** |
|  | (0.06) |  | (0.06) |  | (0.07) |  | (0.07) |  | (0.07) |  | (0.07) |  | (0.07) |  |
| Population density | 0.14 | ** | 0.13 | ** | 0.14 | ** | 0.13 | ** | 0.13 | ** | 0.14 | ** | 0.13 | ** |
|  | (0.02) |  | (0.02) |  | (0.02) |  | (0.02) |  | (0.02) |  | (0.02) |  | (0.02) |  |
| Population growth | 0.25 | ** | 0.23 | ** | 0.25 | ** | 0.22 | ** | 0.23 | ** | 0.25 | ** | 0.23 | ** |
|  | (0.06) |  | (0.06) |  | (0.06) |  | (0.06) |  | (0.06) |  | (0.06) |  | (0.06) |  |
| Region West | -0.23 | * | -0.18 | + | -0.23 | * | -0.26 | ** | -0.22 | * | -0.23 | * | -0.23 | * |
|  | (0.10) |  | (0.10) |  | (0.11) |  | (0.10) |  | (0.10) |  | (0.10) |  | (0.10) |  |
| Region Midwest | -0.36 | ** | -0.41 | ** | -0.36 | ** | -0.40 | ** | -0.39 | ** | -0.37 | ** | -0.39 | ** |
|  | (0.11) |  | (0.11) |  | (0.11) |  | (0.11) |  | (0.11) |  | (0.11) |  | (0.11) |  |
| Region Northeast | 0.04 |  | 0.06 |  | 0.04 |  | 0.05 |  | 0.02 |  | 0.04 |  | 0.04 |  |
|  | (0.14) |  | (0.14) |  | (0.14) |  | (0.14) |  | (0.14) |  | (0.14) |  | (0.14) |  |
| Constant | 4.17 | ** | 4.23 | ** | 4.17 | ** | 4.24 | ** | 4.21 | ** | 4.17 | ** | 4.20 | ** |
|  | (0.07) |  | (0.07) |  | (0.08) |  | (0.08) |  | (0.07) |  | (0.07) |  | (0.07) |  |
|  |  |  |  |  |  |  |  |  |  |  |  |  |  |  |
| Observations | 366 |  | 366 |  | 366 |  | 366 |  | 366 |  | 366 |  | 366 |  |
| Adjusted R2 | 0.634 |  | 0.650 |  | 0.633 |  | 0.639 |  | 0.637 |  | 0.633 |  | 0.635 |  |
| F test | 43.08 |  | 43.31 |  | 40.28 |  | 41.40 |  | 41.04 |  | 40.28 |  | 40.76 |  |
| AIC | 729 |  | 713.6 |  | 731 |  | 724.4 |  | 726.5 |  | 731 |  | 728.2 |  |

The OLS regressions are weighted by the number of observations per region in the personality data file giving more weight to regions with more observations. Displaying unstandardized coefficients and standard errors in parentheses. **, *, + = 1%, 5%, 10% significance level.

**Table A14: Probability of being an entrepreneur in 22 occupational groups in GB**

| Occupation | Probability of being an entrepreneur | Difference of the average personality profile of self-employed and employed |
| --- | --- | --- |
| Accounting / finance | .075 | 1.53 |
| Administration | .022 | 1.11 |
| Business development | .148 | 1.53 |
| Consultancy | .370 | 1.08 |
| Customer service | .036 | 1.51 |
| Education / training | .050 | 0.91 |
| Engineering / R and D | .071 | 1.65 |
| Executive / senior management | .119 | 0.51 |
| Healthcare | .063 | 1.49 |
| IT | .108 | 1.58 |
| General management | .058 | 0.70 |
| Government / military | .003 | 2.49 |
| Homemaker | .220 | 1.71 |
| Legal | .112 | 1.40 |
| Manufacturing / operations | .075 | 1.79 |
| Media | .280 | 0.56 |
| Medical / science | .061 | 0.57 |
| Personnel | .044 | 1.50 |
| Professional | .124 | 0.85 |
| Purchasing | .044 | 2.15 |
| Sales / marketing / advertising | .118 | 0.98 |
| Skilled labour | .376 | 1.13 |
| Other | .256 | 1.57 |

**Table A15: Individual entrepreneurship, occupation and regional characteristics**

|  | GB: Dependent variable: 1=being an entrepreneur, 0=paid employee | | | | | | | | |
| --- | --- | --- | --- | --- | --- | --- | --- | --- | --- |
|  | Model 1 - Base | | | Model 2 - Occupation | | | Model 3 - Region | | |
|  | Coef | Sig | AME | Coef | Sig | AME | Coef | Sig | AME |
| *Individual level variables* |  |  |  |  |  |  |  |  |  |
| Age (in years) | 1.107 | ** | 0.101 | 1.319 | ** | 0.109 | 1.328 | ** | 0.109 |
|  | (0.037) |  | (0.003) | (0.039) |  | (0.003) | (0.040) |  | (0.003) |
| Age squared | -0.607 | ** | -0.056 | -0.746 | ** | -0.061 | -0.762 | ** | -0.063 |
|  | (0.035) |  | (0.003) | (0.037) |  | (0.003) | (0.038) |  | (0.003) |
| Gender (1=male; 0=female) | 0.595 | ** | 0.055 | 0.301 | ** | 0.025 | 0.310 | ** | 0.025 |
|  | (0.014) |  | (0.001) | (0.015) |  | (0.001) | (0.015) |  | (0.001) |
| Income (7 categories) | -0.030 | ** | -0.003 | 0.012 |  | 0.001 | 0.002 |  | 0.000 |
|  | (0.007) |  | (0.001) | (0.008) |  | (0.001) | (0.008) |  | (0.001) |
| White | -0.102 | ** | -0.009 | -0.076 | + | -0.006 | -0.075 | + | -0.006 |
|  | (0.039) |  | (0.004) | (0.042) |  | (0.003) | (0.042) |  | (0.003) |
| Asian | 0.040 |  | 0.004 | 0.141 | * | 0.012 | 0.151 | * | 0.012 |
|  | (0.056) |  | (0.005) | (0.059) |  | (0.005) | (0.059) |  | (0.005) |
| Mixed | 0.053 |  | 0.005 | 0.038 |  | 0.003 | 0.038 |  | 0.003 |
|  | (0.063) |  | (0.006) | (0.066) |  | (0.005) | (0.066) |  | (0.005) |
| Black | -0.143 | + | -0.013 | -0.080 |  | -0.007 | -0.080 |  | -0.007 |
|  | (0.079) |  | (0.007) | (0.083) |  | (0.007) | (0.083) |  | (0.007) |
| Education | -0.027 | + | -0.002 | 0.032 | * | 0.003 | 0.022 |  | 0.002 |
| (1=at least bachelor degree) | (0.014) |  | (0.001) | (0.016) |  | (0.001) | (0.016) |  | (0.001) |
| Entrepreneurial personality | 0.205 | ** | 0.019 | 0.170 | ** | 0.014 | 0.167 | ** | 0.014 |
| profile | (0.007) |  | (0.001) | (0.008) |  | (0.001) | (0.008) |  | (0.001) |
| *Individual level occupations* |  |  |  |  |  |  |  |  |  |
| Accounting/finance |  |  |  | -1.542 | ** | -0.127 | -1.543 | ** | -0.127 |
|  |  |  |  | (0.037) |  | (0.003) | (0.037) |  | (0.003) |
| Administration |  |  |  | -2.765 | ** | -0.228 | -2.762 | ** | -0.227 |
|  |  |  |  | (0.047) |  | (0.004) | (0.047) |  | (0.004) |
| Business development |  |  |  | -0.869 | ** | -0.072 | -0.867 | ** | -0.071 |
|  |  |  |  | (0.058) |  | (0.005) | (0.059) |  | (0.005) |
| Consultancy |  |  |  | 0.216 | ** | 0.018 | 0.207 | ** | 0.017 |
|  |  |  |  | (0.034) |  | (0.003) | (0.034) |  | (0.003) |
| Customer service |  |  |  | -2.065 | ** | -0.170 | -2.056 | ** | -0.169 |
|  |  |  |  | (0.053) |  | (0.004) | (0.053) |  | (0.004) |
| Education / training |  |  |  | -2.032 | ** | -0.167 | -2.019 | ** | -0.166 |
|  |  |  |  | (0.031) |  | (0.003) | (0.031) |  | (0.002) |
| Engineering / R and D |  |  |  | -1.756 | ** | -0.145 | -1.742 | ** | -0.143 |
|  |  |  |  | (0.050) |  | (0.004) | (0.051) |  | (0.004) |
| Executive / senior management |  |  |  | -1.523 | ** | -0.126 | -1.529 | ** | -0.126 |
|  |  |  |  | (0.047) |  | (0.004) | (0.048) |  | (0.004) |
| Healthcare |  |  |  | -1.730 | ** | -0.143 | -1.717 | ** | -0.141 |
|  |  |  |  | (0.036) |  | (0.003) | (0.036) |  | (0.003) |
| IT |  |  |  | -1.275 | ** | -0.105 | -1.269 | ** | -0.104 |
|  |  |  |  | (0.033) |  | (0.003) | (0.033) |  | (0.003) |
| General management |  |  |  | -2.046 | ** | -0.169 | -2.042 | ** | -0.168 |
|  |  |  |  | (0.056) |  | (0.005) | (0.056) |  | (0.005) |
| Government / military |  |  |  | -4.903 | ** | -0.404 | -4.900 | ** | -0.402 |
|  |  |  |  | (0.190) |  | (0.016) | (0.190) |  | (0.016) |
| Homemaker |  |  |  | -0.309 | * | -0.025 | -0.324 | * | -0.027 |
|  |  |  |  | (0.136) |  | (0.011) | (0.137) |  | (0.011) |
| Legal |  |  |  | -1.020 | ** | -0.084 | -1.016 | ** | -0.083 |
|  |  |  |  | (0.052) |  | (0.004) | (0.052) |  | (0.004) |
| Manufacturing/operations |  |  |  | -1.745 | ** | -0.144 | -1.708 | ** | -0.140 |
|  |  |  |  | (0.070) |  | (0.006) | (0.070) |  | (0.006) |
| Media |  |  |  | 0.228 | ** | 0.019 | 0.205 | ** | 0.017 |
|  |  |  |  | (0.032) |  | (0.003) | (0.033) |  | (0.003) |
| Medical/science |  |  |  | -1.703 | ** | -0.140 | -1.697 | ** | -0.139 |
|  |  |  |  | (0.046) |  | (0.004) | (0.046) |  | (0.004) |
| Personnel |  |  |  | -2.045 | ** | -0.168 | -2.040 | ** | -0.168 |
|  |  |  |  | (0.101) |  | (0.008) | (0.101) |  | (0.008) |
| Professional |  |  |  | -1.105 | ** | -0.091 | -1.092 | ** | -0.090 |
|  |  |  |  | (0.028) |  | (0.002) | (0.028) |  | (0.002) |
| Purchasing |  |  |  | -2.138 | ** | -0.176 | -2.119 | ** | -0.174 |
|  |  |  |  | (0.146) |  | (0.012) | (0.146) |  | (0.012) |
| Sales / marketing / advertising |  |  |  | -0.947 | ** | -0.078 | -0.951 | ** | -0.078 |
|  |  |  |  | (0.031) |  | (0.003) | (0.031) |  | (0.003) |
| Skilled labour |  |  |  | 0.436 | ** | 0.036 | 0.437 | ** | 0.036 |
|  |  |  |  | (0.033) |  | (0.003) | (0.033) |  | (0.003) |
| *Regional level variables* |  |  |  |  |  |  |  |  |  |
| Entrepreneurial culture |  |  |  |  |  |  | 0.071 | ** | 0.006 |
|  |  |  |  |  |  |  | (0.012) |  | (0.001) |
| Human capital |  |  |  |  |  |  | 0.023 |  | 0.002 |
|  |  |  |  |  |  |  | (0.014) |  | (0.001) |
| Industry diversity |  |  |  |  |  |  | 0.022 | * | 0.002 |
|  |  |  |  |  |  |  | (0.010) |  | (0.001) |
| Unemployment rate (mean) |  |  |  |  |  |  | -0.056 | ** | -0.005 |
|  |  |  |  |  |  |  | (0.014) |  | (0.001) |
| Unemployment rate (change) |  |  |  |  |  |  | -0.015 | + | -0.001 |
|  |  |  |  |  |  |  | (0.009) |  | (0.001) |
| Per capita income (mean) |  |  |  |  |  |  | -0.009 |  | -0.001 |
|  |  |  |  |  |  |  | (0.014) |  | (0.001) |
| Per capita income (change) |  |  |  |  |  |  | -0.018 | * | -0.001 |
|  |  |  |  |  |  |  | (0.007) |  | (0.001) |
| Migration |  |  |  |  |  |  | 0.037 | * | 0.003 |
|  |  |  |  |  |  |  | (0.017) |  | (0.001) |
| Age group 25-44 |  |  |  |  |  |  | -0.203 | ** | -0.017 |
|  |  |  |  |  |  |  | (0.020) |  | (0.002) |
| Population density |  |  |  |  |  |  | 0.113 | ** | 0.009 |
|  |  |  |  |  |  |  | (0.019) |  | (0.002) |
| Population growth |  |  |  |  |  |  | 0.041 | ** | 0.003 |
|  |  |  |  |  |  |  | (0.010) |  | (0.001) |
| Region England |  |  |  |  |  |  | 0.016 |  | 0.001 |
|  |  |  |  |  |  |  | (0.032) |  | (0.003) |
| Region Wales |  |  |  |  |  |  | 0.092 | + | 0.008 |
|  |  |  |  |  |  |  | (0.047) |  | (0.004) |
| Constant | -2.401 |  |  | -1.175 |  |  | -1.207 |  |  |
|  | (0.040) |  |  | (0.045) |  |  | (0.054) |  |  |
| Observations | 247,232 |  |  | 247,232 |  |  | 247,232 |  |  |
| Pseudo R^2^ | 0.0536 |  |  | 0.165 |  |  | 0.168 |  |  |
| Wald Chi2 | 9001 |  |  | 27655 |  |  | 28162 |  |  |

**Table A15 (cont.): Individual entrepreneurship, occupation and regional characteristics**

|  | GB Dependent variable: 1=Being an entrepreneur, 0=paid employee | | | | | | | | | | | |
| --- | --- | --- | --- | --- | --- | --- | --- | --- | --- | --- | --- | --- |
|  | Model 4 -Interaction Human Capital | | | Model 5 - Interaction Industry Diversity | | | Model 6 -Multilevel Human Capital | | | Model 7 - Multilevel Industry Diversity | | |
|  | Coef |  | AME | Coef |  | AME | Coef |  | AME | Coef |  | AME |
| *Individual level variables* |  |  |  |  |  |  |  |  |  |  |  |  |
| Age (in years) | 1.327 | ** | 0.109 | 1.328 | ** | 0.109 | 1.327 | ** | 0.105 | 1.327 | ** | 0.105 |
|  | (0.040) |  | (0.003) | (0.040) |  | (0.003) | (0.040) |  | (0.003) | (0.040) |  | (0.003) |
| Age squared | -0.762 | ** | -0.063 | -0.762 | ** | -0.063 | -0.763 | ** | -0.060 | -0.763 | ** | -0.060 |
|  | (0.038) |  | (0.003) | (0.038) |  | (0.003) | (0.038) |  | (0.003) | (0.038) |  | (0.003) |
| Gender (1=male; 0=female) | 0.310 | ** | 0.025 | 0.310 | ** | 0.025 | 0.313 | ** | 0.025 | 0.313 | ** | 0.025 |
|  | (0.015) |  | (0.001) | (0.015) |  | (0.001) | (0.015) |  | (0.001) | (0.015) |  | (0.001) |
| Income (7 categories) | 0.002 |  | 0.000 | 0.002 |  | 0.000 | 0.004 |  | 0.000 | 0.004 |  | 0.000 |
|  | (0.008) |  | (0.001) | (0.008) |  | (0.001) | (0.008) |  | (0.001) | (0.008) |  | (0.001) |
| White | -0.075 | + | -0.006 | -0.075 | + | -0.006 | -0.074 | + | -0.006 | -0.074 | + | -0.006 |
|  | (0.042) |  | (0.003) | (0.042) |  | (0.003) | (0.042) |  | (0.003) | (0.042) |  | (0.003) |
| Asian | 0.151 | * | 0.012 | 0.151 | * | 0.012 | 0.157 | ** | 0.012 | 0.157 | ** | 0.012 |
|  | (0.059) |  | (0.005) | (0.059) |  | (0.005) | (0.059) |  | (0.005) | (0.059) |  | (0.005) |
| Mixed | 0.038 |  | 0.003 | 0.038 |  | 0.003 | 0.037 |  | 0.003 | 0.037 |  | 0.003 |
|  | (0.066) |  | (0.005) | (0.066) |  | (0.005) | (0.066) |  | (0.005) | (0.066) |  | (0.005) |
| Black | -0.079 |  | -0.006 | -0.079 |  | -0.007 | -0.083 |  | -0.007 | -0.083 |  | -0.007 |
|  | (0.083) |  | (0.007) | (0.083) |  | (0.007) | (0.083) |  | (0.007) | (0.083) |  | (0.007) |
| Education | 0.022 |  | 0.002 | 0.022 |  | 0.002 | 0.020 |  | 0.002 | 0.020 |  | 0.002 |
| (1=at least bachelor degree) | (0.016) |  | (0.001) | (0.016) |  | (0.001) | (0.016) |  | (0.001) | (0.016) |  | (0.001) |
| Entrepreneurial personality | 0.167 | ** | 0.014 | 0.167 | ** | 0.014 | 0.168 | ** | 0.013 | 0.168 | ** | 0.013 |
| profile | (0.008) |  | (0.001) | (0.008) |  | (0.001) | (0.008) |  | (0.001) | (0.008) |  | (0.001) |
| *Individual level occupations* |  |  |  |  |  |  |  |  |  |  |  |  |
| Accounting/finance | -1.542 | ** | -0.127 | -1.542 | ** | -0.127 | -1.541 | ** | -0.122 | -1.541 | ** | -0.122 |
|  | (0.037) |  | (0.003) | (0.037) |  | (0.003) | (0.037) |  | (0.003) | (0.037) |  | (0.003) |
| Administration | -2.761 | ** | -0.227 | -2.761 | ** | -0.227 | -2.756 | ** | -0.218 | -2.756 | ** | -0.218 |
|  | (0.047) |  | (0.004) | (0.047) |  | (0.004) | (0.047) |  | (0.004) | (0.047) |  | (0.004) |
| Business development | -0.867 | ** | -0.071 | -0.867 | ** | -0.071 | -0.864 | ** | -0.068 | -0.864 | ** | -0.068 |
|  | (0.059) |  | (0.005) | (0.059) |  | (0.005) | (0.059) |  | (0.005) | (0.059) |  | (0.005) |
| Consultancy | 0.207 | ** | 0.017 | 0.207 | ** | 0.017 | 0.208 | ** | 0.016 | 0.209 | ** | 0.016 |
|  | (0.034) |  | (0.003) | (0.034) |  | (0.003) | (0.034) |  | (0.003) | (0.034) |  | (0.003) |
| Customer service | -2.056 | ** | -0.169 | -2.056 | ** | -0.169 | -2.054 | ** | -0.162 | -2.054 | ** | -0.162 |
|  | (0.053) |  | (0.004) | (0.053) |  | (0.004) | (0.053) |  | (0.004) | (0.053) |  | (0.004) |
| Education / training | -2.019 | ** | -0.166 | -2.019 | ** | -0.166 | -2.017 | ** | -0.160 | -2.017 | ** | -0.159 |
|  | (0.031) |  | (0.002) | (0.031) |  | (0.002) | (0.031) |  | (0.003) | (0.031) |  | (0.003) |
| Engineering / R and D | -1.741 | ** | -0.143 | -1.741 | ** | -0.143 | -1.732 | ** | -0.137 | -1.732 | ** | -0.137 |
|  | (0.051) |  | (0.004) | (0.051) |  | (0.004) | (0.051) |  | (0.004) | (0.051) |  | (0.004) |
| Executive / senior management | -1.529 | ** | -0.126 | -1.529 | ** | -0.126 | -1.527 | ** | -0.121 | -1.527 | ** | -0.121 |
|  | (0.048) |  | (0.004) | (0.048) |  | (0.004) | (0.048) |  | (0.004) | (0.048) |  | (0.004) |
| Healthcare | -1.716 | ** | -0.141 | -1.716 | ** | -0.141 | -1.714 | ** | -0.136 | -1.714 | ** | -0.135 |
|  | (0.036) |  | (0.003) | (0.036) |  | (0.003) | (0.036) |  | (0.003) | (0.036) |  | (0.003) |
| IT | -1.269 | ** | -0.104 | -1.269 | ** | -0.104 | -1.264 | ** | -0.100 | -1.263 | ** | -0.100 |
|  | (0.033) |  | (0.003) | (0.033) |  | (0.003) | (0.033) |  | (0.003) | (0.033) |  | (0.003) |
| General management | -2.041 | ** | -0.168 | -2.041 | ** | -0.168 | -2.041 | ** | -0.161 | -2.041 | ** | -0.161 |
|  | (0.056) |  | (0.005) | (0.056) |  | (0.005) | (0.056) |  | (0.005) | (0.056) |  | (0.005) |
| Government / military | -4.900 | ** | -0.402 | -4.900 | ** | -0.402 | -4.903 | ** | -0.388 | -4.903 | ** | -0.387 |
|  | (0.190) |  | (0.016) | (0.190) |  | (0.016) | (0.190) |  | (0.015) | (0.190) |  | (0.015) |
| Homemaker | -0.321 | * | -0.026 | -0.323 | * | -0.027 | -0.313 | * | -0.025 | -0.313 | * | -0.025 |
|  | (0.137) |  | (0.011) | (0.137) |  | (0.011) | (0.137) |  | (0.011) | (0.137) |  | (0.011) |
| Legal | -1.016 | ** | -0.083 | -1.016 | ** | -0.083 | -1.020 | ** | -0.081 | -1.020 | ** | -0.081 |
|  | (0.052) |  | (0.004) | (0.052) |  | (0.004) | (0.053) |  | (0.004) | (0.053) |  | (0.004) |
| Manufacturing/operations | -1.708 | ** | -0.140 | -1.708 | ** | -0.140 | -1.707 | ** | -0.135 | -1.707 | ** | -0.135 |
|  | (0.070) |  | (0.006) | (0.070) |  | (0.006) | (0.071) |  | (0.006) | (0.071) |  | (0.006) |
| Media | 0.205 | ** | 0.017 | 0.205 | ** | 0.017 | 0.195 | ** | 0.015 | 0.195 | ** | 0.015 |
|  | (0.033) |  | (0.003) | (0.033) |  | (0.003) | (0.033) |  | (0.003) | (0.033) |  | (0.003) |
| Medical/science | -1.695 | ** | -0.139 | -1.696 | ** | -0.139 | -1.688 | ** | -0.134 | -1.688 | ** | -0.133 |
|  | (0.046) |  | (0.004) | (0.046) |  | (0.004) | (0.046) |  | (0.004) | (0.046) |  | (0.004) |
| Personnel | -2.039 | ** | -0.167 | -2.039 | ** | -0.167 | -2.028 | ** | -0.160 | -2.028 | ** | -0.160 |
|  | (0.101) |  | (0.008) | (0.101) |  | (0.008) | (0.101) |  | (0.008) | (0.101) |  | (0.008) |
| Professional | -1.091 | ** | -0.090 | -1.091 | ** | -0.090 | -1.089 | ** | -0.086 | -1.089 | ** | -0.086 |
|  | (0.028) |  | (0.002) | (0.028) |  | (0.002) | (0.029) |  | (0.002) | (0.029) |  | (0.002) |
| Purchasing | -2.118 | ** | -0.174 | -2.119 | ** | -0.174 | -2.119 | ** | -0.168 | -2.120 | ** | -0.167 |
|  | (0.146) |  | (0.012) | (0.146) |  | (0.012) | (0.146) |  | (0.012) | (0.146) |  | (0.012) |
| Sales / marketing / advertising | -0.951 | ** | -0.078 | -0.951 | ** | -0.078 | -0.951 | ** | -0.075 | -0.951 | ** | -0.075 |
|  | (0.031) |  | (0.003) | (0.031) |  | (0.003) | (0.031) |  | (0.003) | (0.031) |  | (0.003) |
| Skilled labour | 0.436 | ** | 0.036 | 0.437 | ** | 0.036 | 0.437 | ** | 0.035 | 0.437 | ** | 0.035 |
|  | (0.033) |  | (0.003) | (0.033) |  | (0.003) | (0.033) |  | (0.003) | (0.033) |  | (0.003) |
| *Regional level variables* |  |  |  |  |  |  |  |  |  |  |  |  |
| Entrepreneurial culture | 0.077 | ** | 0.007 | 0.074 | ** | 0.006 | 0.062 | ** | 0.005 | 0.059 | ** | 0.005 |
|  | (0.012) |  | (0.001) | (0.012) |  | (0.001) | (0.016) |  | (0.001) | (0.016) |  | (0.001) |
| Human capital | 0.030 | * | 0.003 | 0.026 | + | 0.002 | 0.036 | + | 0.003 | 0.032 |  | 0.003 |
|  | (0.014) |  | (0.001) | (0.014) |  | (0.001) | (0.020) |  | (0.002) | (0.019) |  | (0.002) |
| Industry diversity | 0.030 | ** | 0.002 | 0.026 | * | 0.002 | 0.046 | ** | 0.004 | 0.044 | ** | 0.004 |
|  | (0.011) |  | (0.001) | (0.011) |  | (0.001) | (0.015) |  | (0.001) | (0.016) |  | (0.001) |
| Interaction: Human capital X | 0.033 | ** |  |  |  |  | 0.024 | * |  |  |  |  |
| Entrepreneurial culture | (0.009) |  |  |  |  |  | (0.012) |  |  |  |  |  |
| Interaction: Industry diversity X |  |  |  | 0.013 | + |  |  |  |  | 0.011 |  |  |
| Entrepreneurial culture |  |  |  | (0.007) |  |  |  |  |  | (0.009) |  |  |
| Unemployment rate (mean) | -0.067 | ** | -0.005 | -0.060 | ** | -0.005 | -0.063 | ** | -0.005 | -0.058 | ** | -0.005 |
|  | (0.015) |  | (0.001) | (0.014) |  | (0.001) | (0.020) |  | (0.002) | (0.020) |  | (0.002) |
| Unemployment rate (change) | -0.015 | + | -0.001 | -0.014 |  | -0.001 | -0.024 | + | -0.002 | -0.023 | + | -0.002 |
|  | (0.009) |  | (0.001) | (0.009) |  | (0.001) | (0.012) |  | (0.001) | (0.012) |  | (0.001) |
| Per capita income (mean) | -0.032 | * | -0.003 | -0.019 |  | -0.002 | -0.077 | ** | -0.006 | -0.070 | ** | -0.006 |
|  | (0.015) |  | (0.001) | (0.015) |  | (0.001) | (0.024) |  | (0.002) | (0.024) |  | (0.002) |
| Per capita income (change) | -0.017 | * | -0.001 | -0.016 | * | -0.001 | -0.015 | + | -0.001 | -0.015 |  | -0.001 |
|  | (0.007) |  | (0.001) | (0.007) |  | (0.001) | (0.009) |  | (0.001) | (0.009) |  | (0.001) |
| Migration | 0.025 |  | 0.002 | 0.034 | * | 0.003 | 0.031 |  | 0.002 | 0.037 |  | 0.003 |
|  | (0.017) |  | (0.001) | (0.017) |  | (0.001) | (0.023) |  | (0.002) | (0.023) |  | (0.002) |
| Age group 25-44 | -0.197 | ** | -0.016 | -0.201 | ** | -0.017 | -0.197 | ** | -0.016 | -0.201 | ** | -0.016 |
|  | (0.020) |  | (0.002) | (0.020) |  | (0.002) | (0.028) |  | (0.002) | (0.028) |  | (0.002) |
| Population density | 0.111 | ** | 0.009 | 0.116 | ** | 0.009 | 0.013 |  | 0.001 | 0.015 |  | 0.001 |
|  | (0.019) |  | (0.002) | (0.019) |  | (0.002) | (0.034) |  | (0.003) | (0.034) |  | (0.003) |
| Population growth | 0.046 | ** | 0.004 | 0.043 | ** | 0.004 | 0.048 | ** | 0.004 | 0.045 | ** | 0.004 |
|  | (0.010) |  | (0.001) | (0.010) |  | (0.001) | (0.014) |  | (0.001) | (0.014) |  | (0.001) |
| Region England | 0.007 |  | 0.001 | 0.016 |  | 0.001 | 0.059 |  | 0.005 | 0.066 |  | 0.005 |
|  | (0.032) |  | (0.003) | (0.032) |  | (0.003) | (0.045) |  | (0.004) | (0.045) |  | (0.004) |
| Region Wales | 0.085 | + | 0.007 | 0.089 | + | 0.007 | 0.110 | + | 0.009 | 0.112 | + | 0.009 |
|  | (0.048) |  | (0.004) | (0.048) |  | (0.004) | (0.064) |  | (0.005) | (0.064) |  | (0.005) |
| Constant | -1.222 |  |  | -1.215 |  |  | -1.325 |  |  | -1.323 |  |  |
|  | (0.054) |  |  | (0.054) |  |  | (0.063) |  |  | (0.063) |  |  |
| Observations | 247,232 |  |  | 247,232 |  |  | 247,232 |  |  | 247,232 |  |  |
| Pseudo R^2^ | 0.168 |  |  | 0.168 |  |  | 0.334 |  |  | 0.334 |  |  |
| Wald Chi2 | 28175 |  |  | 28165 |  |  | 21755 |  |  | 21748 |  |  |
| Number of groups | -- |  |  | -- |  |  | 375 |  |  | 375 |  |  |

Notes: Models 1-5 are standard logistic regressions; Models 6-7 random effects multilevel model. All non-binary variables are zstandardized. Displaying regression coefficients, average marginal effects (AME) and standard errors in parentheses. In order to compute the Pseudo R² in a multilevel setting, we follow recommendations of Snijders and Bosker (1999). According to them Pseudo R² is calculated by dividing the variance of the predicted residuals of the estimated model by the sum of (1) the variance of predicted residuals of the estimated model, (2) the level-2 variance and (3) the level-1 variance, which is equal to p2/3 in a logistic model. **, *, + = 1%, 5%, 10% significance level.

**Table A16: 95% confidence intervals of the of the predicted probabilities to be an entrepreneur**

|  |  | Entrepreneurial culture | |
| --- | --- | --- | --- |
|  |  | 1 SD below mean | 1 SD above mean |
| Human capital | 1 SD below mean | .096 - .101 | .101 - .110 |
|  | 1 SD above mean | .094 - .102 | .113 - .119 |
| Industry diversity | 1 SD below mean | .096 - .101 | .105 - .112 |
|  | 1 SD above mean | .097 - .104 | .112 - .118 |

**Table A17. Entrepreneurship rate, human capital, industry diversity, entrepreneurial culture weighted by age and gender at the regional level, and interactions.**

|  | Dependent variable: Entrepreneurship rate | | | | | | | | | | | | |
| --- | --- | --- | --- | --- | --- | --- | --- | --- | --- | --- | --- | --- | --- |
|  |  |  | US |  |  |  |  |  | GB |  |  |  |  |
| Variables | Model 1 |  | Model 2 |  | Model 3 |  | Model 4 |  | Model 5 |  | Model 6 |  |  |
| Human capital | 0.23 | ** | 0.18 | * | 0.20 | * | -0.14 |  | -0.15 |  | -0.06 |  |  |
|  | (0.08) |  | (0.08) |  | (0.08) |  | (0.17) |  | (0.16) |  | (0.16) |  |  |
| Industry diversity | 0.35 | ** | 0.34 | ** | 0.28 | ** | 0.32 | * | 0.45 | ** | 0.32 | * |  |
|  | (0.06) |  | (0.06) |  | (0.06) |  | (0.14) |  | (0.14) |  | (0.13) |  |  |
| Entrepreneurial culture | 0.19 | ** | 0.17 | ** | 0.19 | ** | 0.58 | ** | 0.57 | ** | 0.60 | ** |  |
|  | (0.06) |  | (0.06) |  | (0.06) |  | (0.14) |  | (0.14) |  | (0.13) |  |  |
| Interaction: Human capital X | ---- |  | 0.17 | ** | ---- |  | ---- |  | 0.54 | ** | ---- |  |  |
| Entrepreneurial culture |  |  | (0.06) |  |  |  |  |  | (0.11) |  |  |  |  |
| Interaction: Industry diversity X | ---- |  | ---- |  | 0.13 | ** | ---- |  | ---- |  | 0.55 | ** |  |
| Entrepreneurial culture |  |  |  |  | (0.05) |  |  |  |  |  | (0.10) |  |  |
| Unemployment rate (mean) | 0.02 |  | 0.02 |  | 0.01 |  | -0.09 |  | -0.14 |  | -0.13 |  |  |
|  | (0.07) |  | (0.07) |  | (0.07) |  | (0.17) |  | (0.17) |  | (0.17) |  |  |
| Unemployment rate (change) | 0.29 | ** | 0.29 | ** | 0.28 | ** | 0.16 |  | 0.16 |  | 0.20 |  |  |
|  | (0.05) |  | (0.05) |  | (0.05) |  | (0.12) |  | (0.11) |  | (0.11) |  |  |
| Per capita income (mean) | 0.06 |  | 0.06 |  | 0.09 |  | 2.16 | ** | 1.90 | ** | 1.93 | ** |  |
|  | (0.06) |  | (0.06) |  | (0.06) |  | (0.14) |  | (0.15) |  | (0.14) |  |  |
| Per capita income (change) | -0.12 |  | -0.13 |  | -0.12 |  | -0.13 |  | -0.14 |  | -0.07 |  |  |
|  | (0.07) |  | (0.07) |  | (0.07) |  | (0.11) |  | (0.11) |  | (0.11) |  |  |
| Migration | 0.16 | ** | 0.17 | ** | 0.13 | * | 0.03 |  | -0.06 |  | -0.00 |  |  |
|  | (0.06) |  | (0.06) |  | (0.06) |  | (0.15) |  | (0.15) |  | (0.15) |  |  |
| Age group 25-44 | -0.23 | ** | -0.25 | ** | -0.22 | ** | -0.76 | ** | -0.74 | ** | -0.76 | ** |  |
|  | (0.06) |  | (0.06) |  | (0.06) |  | (0.17) |  | (0.16) |  | (0.16) |  |  |
| Population density | 0.14 | ** | 0.14 | ** | 0.13 | ** | 1.02 | ** | 0.97 | ** | 1.03 | ** |  |
|  | (0.02) |  | (0.02) |  | (0.02) |  | (0.12) |  | (0.12) |  | (0.11) |  |  |
| Population growth | 0.24 | ** | 0.26 | ** | 0.25 | ** | 0.48 | ** | 0.56 | ** | 0.52 | ** |  |
|  | (0.06) |  | (0.06) |  | (0.06) |  | (0.11) |  | (0.11) |  | (0.11) |  |  |
| Region West | -0.22 | * | -0.23 | * | -0.23 | * | ---- |  | ---- |  | ---- |  |  |
|  | (0.10) |  | (0.10) |  | (0.10) |  |  |  |  |  |  |  |  |
| Region Midwest | -0.35 | ** | -0.32 | ** | -0.36 | ** | ---- |  | ---- |  | ---- |  |  |
|  | (0.11) |  | (0.11) |  | (0.11) |  |  |  |  |  |  |  |  |
| Region Northeast | -0.01 |  | 0.05 |  | -0.03 |  | ---- |  | ---- |  | ---- |  |  |
|  | (0.14) |  | (0.14) |  | (0.14) |  |  |  |  |  |  |  |  |
| Region England | ---- |  | ---- |  | ---- |  | 0.77 |  | 0.65 |  | 0.72 |  |  |
|  |  |  |  |  |  |  | (0.39) |  | (0.38) |  | (0.38) |  |  |
| Region Wales | ---- |  | ---- |  | ---- |  | 0.32 |  | 0.22 |  | 0.21 |  |  |
|  |  |  |  |  |  |  | (0.59) |  | (0.57) |  | (0.56) |  |  |
| Constant | 4.20 | ** | 4.17 | ** | 4.19 | ** | 8.97 | ** | 8.88 | ** | 8.85 | ** |  |
|  | (0.07) |  | (0.07) |  | (0.07) |  | (0.38) |  | (0.37) |  | (0.36) |  |  |
|  |  |  |  |  |  |  |  |  |  |  |  |  |  |
| Observations | 366 |  | 366 |  | 366 |  | 375 |  | 375 |  | 375 |  |  |
| Adjusted R^2^ | 0.625 |  | 0.634 |  | 0.633 |  | 0.840 |  | 0.849 |  | 0.852 |  |  |
| F test | 44.54 | ** | 43.13 | ** | 42.90 | ** | 151.9 | ** | 151.3 | ** | 154.9 | ** |  |
| AIC | 736.1 |  | 728.7 |  | 730 |  | 1492 |  | 1471 |  | 1471 |  |  |

The independent variables are industry diversity, human capital, and entrepreneurial culture based on current residence. The OLS regressions are weighted by the number of observations per region in the personality data file giving more weight to regions with more observations. Displaying unstandardized coefficients and standard errors in parentheses. **, * = 1%, 5% significance level.

**Table A18. Number of regions with no Fortune 100 firm vs. at least one Fortune 100 firm by interaction groups (human capital and entrepreneurial culture) in the US.**

|  | Interaction groups between human capital and entrepreneurial culture | | |  |
| --- | --- | --- | --- | --- |
|  | low & low | low & high, high & low | high & high | Total |
| no Fortune 100 firm | 121 (99%) | 112 (92%) | 94 (77%) | 327 (89%) |
| at least one Fortune 100 firm | 1 (1%) | 10 (8%) | 28 (23%) | 39 (11%) |
| Total | 122 (100%) | 122 (100%) | 122 (100%) | 366 (100%) |

Displaying number of regions and percentage of regions (in brackets). Both variables, human capital and the entrepreneurial culture were splitted at the median. Regions in the “low & low” column have below median values in human capital and the entrepreneurial culture. Regions in “low & high, high & low” column are above median in either human capital or the entrepreneurial culture. Regions in the “high & high” column have above the median values in human capital and entrepreneurial culture. χ^2^= 32.5, p < 0.001.

**Table A19. Number of regions with no Fortune 100 firm vs. at least one Fortune 100 firm by interaction groups (industry diversity and entrepreneurial culture) in the US.**

|  | Interaction groups between industry diversity and entrepreneurial culture | | |  |
| --- | --- | --- | --- | --- |
|  | low & low | low & high, high & low | high & high | Total |
| no Fortune 100 firm | 105 (96%) | 137 (93%) | 85 (78%) | 327 (89%) |
| at least one Fortune 100 firm | 4 (4%) | 11 (7%) | 24 (22%) | 39 (11%) |
| Total | 109 (100%) | 148 (100%) | 109 (100%) | 366 (100%) |

Displaying number of regions and percentage of regions (in brackets). Both variables, industry diversity and the entrepreneurial culture were splitted at the median. Regions in the “low & low” column have below median values in industry diversity and the entrepreneurial culture. Regions in “low & high, high & low” column are above median in either industry diversity or the entrepreneurial culture. Regions in the “high & high” column have above the median values in industry diversity and entrepreneurial culture. χ^2^= 22.0, p < 0.001.

**Table A20. Number of regions with no Fast Track 100 firm vs. at least one Fast Track 100 firm by interaction groups (human capital and entrepreneurial culture) in GB.**

|  | Interaction groups between human capital and entrepreneurial culture | | |  |
| --- | --- | --- | --- | --- |
|  | low & low | low & high, high & low | high & high | Total |
| no Fortune 100 firm | 116 (91%) | 104 (87%) | 101 (79%) | 321 (86%) |
| at least one Fortune 100 firm | 12 (9%) | 15 (13%) | 27 (21%) | 54 (14%) |
| Total | 128 (100%) | 119 (100%) | 128 (100%) | 375 (100%) |

Displaying number of regions and percentage of regions (in brackets). Both variables, human capital and the entrepreneurial culture were splitted at the median. Regions in the “low & low” column have below median values in human capital and the entrepreneurial culture. Regions in “low & high, high & low” column are above median in either human capital or the entrepreneurial culture. Regions in the “high & high” column have above the median values in human capital and entrepreneurial culture. χ^2^= 7.6, p < 0.05.

**Table A21. Number of regions with no Fast Track 100 firm vs. at least one Fast Track 100 firm by interaction groups (industry diversity and entrepreneurial culture) in GB.**

|  | Interaction groups between industry diversity and entrepreneurial culture | | |  |
| --- | --- | --- | --- | --- |
|  | low & low | low & high, high & low | high & high | Total |
| no Fortune 100 firm | 122 (92%) | 92 (83%) | 107 (81%) | 321 (86%) |
| at least one Fortune 100 firm | 10 (8%) | 19 (17%) | 25 (19%) | 54 (14 %) |
| Total | 132 (100%) | 111 (100%) | 132 (100%) | 375 (100%) |

Displaying number of regions and percentage of regions (in brackets). Both variables, industry diversity and the entrepreneurial culture were splitted at the median. Regions in the “low & low” column have below median values in industry diversity and the entrepreneurial culture. Regions in “low & high, high & low” column are above median in either industry diversity or the entrepreneurial culture. Regions in the “high & high” column have above the median values in industry diversity and entrepreneurial culture. χ^2^= 7.9, p < 0.05.


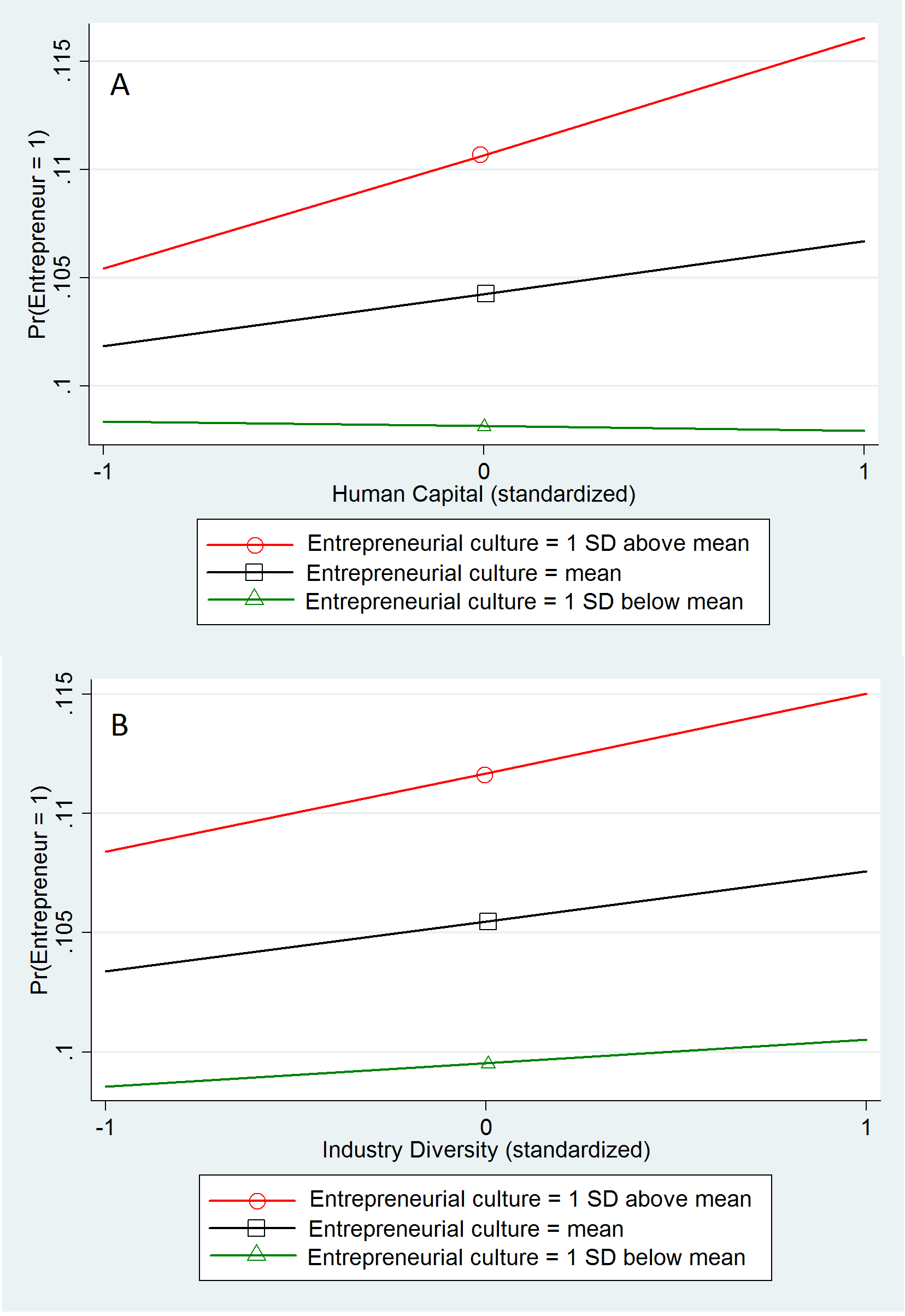


**Fig A1. Interaction plots from logistic regression (GB, N = 375).**

(A) Fig A1A (top): Human capital X Entrepreneurial culture. (B) Fig A1B (bottom): Industry diversity X Entrepreneurial culture.
